# Evolution, heterogeneity and global dispersal of cosmopolitan genotype of Dengue virus type 2

**DOI:** 10.1038/s41598-021-92783-y

**Published:** 2021-06-29

**Authors:** Surya Pavan Yenamandra, Carmen Koo, Suzanna Chiang, Han Shi Jeri Lim, Zhen Yuan Yeo, Lee Ching Ng, Hapuarachchige Chanditha Hapuarachchi

**Affiliations:** 1 Environmental Health Institute, National Environment Agency, 11, Biopolis Way, #06-05-08, Singapore, 138667 Singapore; 2 School of Biological Sciences, Nanyang Technological University, 60 Nanyang Drive, Singapore, 637551 Singapore; 3 NUS Centre for Bioimaging Sciences, National University of Singapore, Block S1A, Level 2, Lee Wee Kheng Building, Science Drive 4, Singapore, 117557 Singapore

**Keywords:** Molecular evolution, Phylogenetics, Dengue virus

## Abstract

Dengue virus type 2 (DENV-2) contributes substantially to the dengue burden and dengue-related mortality in the tropics and sub-tropics. DENV-2 includes six genotypes, among which cosmopolitan genotype is the most widespread. The present study investigated the evolution, intra-genotype heterogeneity and dispersal of cosmopolitan genotype to understand unique genetic characteristics that have shaped the molecular epidemiology and distribution of cosmopolitan lineages. The spatial analysis demonstrated a wide geo-distribution of cosmopolitan genotype through an extensive inter-continental network, anchored in Southeast Asia and Indian sub-continent. Intra-genotype analyses using 3367 envelope gene sequences revealed six distinct lineages within the cosmopolitan genotype, namely the Indian sub-continent lineage and five other lineages. Indian sub-continent lineage was the most diverged among six lineages and has almost reached the nucleotide divergence threshold of 6% within *E* gene to qualify as a separate genotype. Genome wide amino acid signatures and selection pressure analyses further suggested differences in evolutionary characteristics between the Indian sub-continent lineage and other lineages. The present study narrates a comprehensive genomic analysis of cosmopolitan genotype and presents notable genetic characteristics that occurred during its evolution and global expansion. Whether those characteristics conferred a fitness advantage to cosmopolitan genotype in different geographies warrant further investigations.

## Introduction

Dengue is the most common arthropod-borne acute febrile illness, caused by Dengue virus (DENV). Dengue is endemic in the tropics and subtropics, where approximately half of the human population lives at risk^1^. The annual dengue burden worldwide has increased drastically during the last four decades, especially after 1990s^2,3^. DENV is expanding its global footprint amidst environmental and climatic changes that promote the propagation and distribution of its vectors, *Aedes aegypti* and *Aedes albopictus*.

DENV is categorized into four serotypes (DENV-1–4) based on genetic and antigenic characteristics. The inter-serotype nucleotide variability is ~ 30%^4,5^. Each serotype is genetically sub-divided into genotypes^6,7^. Different genotypes show variable adaptability that accompanies genetic modifications in DENV, which may enhance virus dispersal and epidemic potential^8,9^ in different geographies. Among four serotypes, DENV-1 and DENV-2 are the most common types associated with known outbreaks^3^. According to empirical data, the highest pooled mortality rate has been reported during DENV-2 outbreaks^10^. Studies have also shown that DENV-2 causes more severe secondary infections than other serotypes^11,12^. These evidences indicate an important role played by DENV-2 in dengue epidemiology and global health burden.

DENV-2 includes five non-sylvatic genotypes (Asian I, Asian II, American, Asian-American and Cosmopolitan) that are dominant in different regions^13^. Asian I and Asian II genotypes are present in the Asian continent. The already-extinct American genotype was largely restricted to Central and South America. Asian-American genotype circulates in Central and South America as well as in Southeast Asia^14^. Cosmopolitan genotype circulates in the Asia–Pacific, Middle East and Africa and is the most widespread genotype of DENV-2^15–22^. It has expanded further into Uganda, Bhutan, Borneo, Brunei, Japan, Maldives, Micronesia and Tanzania in recent years^23–33^. Given its wide geographical presence, cosmopolitan genotype contributes substantially to the global DENV-2 burden.

Based on current understanding, we hypothesized that DENV-2 cosmopolitan genotype has evolved in parallel to its global dispersal to achieve a higher heterogeneity than other DENV-2 genotypes. The current study, therefore, investigated the evolutionary characteristics and global dispersal patterns of DENV-2 cosmopolitan genotype to decipher genetic heterogeneity, distinctness and geographical dominance. The findings demonstrated that cosmopolitan genotype is spread through an extensive spatial network that is characterized by the dominance of distinct intra-genotype lineages in different geographies.

## Materials and methods

### Sequence data

Three datasets were used in the analyses. The envelope (*E*) gene dataset included 4551 complete *E* gene sequences (1485 bp) of DENV-2 retrieved from the NCBI nucleotide repository. This dataset that included sequences dated from 1944 to 2019 was used to analyse the global distribution of DENV-2 genotypes. The second dataset included 1,048 sequences newly generated at the Environmental Health Institute, as part of the arbovirus surveillance programme in Singapore^34,35^.. These new sequences included those obtained from patient sera (n = 915, inclusive of *E* gene sequences extracted from 158 newly generated complete genomes) and field-caught mosquitoes (n = 133). Sequences obtained from patient sera belonged to all lineages described in the present study (Lineage 1 = 14; lineage 2 = 155; lineage 3 = 23; lineage 4 = 138; lineage 5 = 533; Indian sub-continent lineage = 45; unclassified = 7). Similarly, mosquito-derived sequences belonged to most of the lineages (Lineage 1 = 0; lineage 2 = 29; lineage 3 = 01; lineage 4 = 5; lineage 5 = 96; Indian sub-continent lineage = 02). The entire list of newly generated sequences can be found in Supplementary Table S1. Mosquito derived sequences were identical to those obtained from human sera and were detected in same areas as the latter (Supplementary Fig. S1). Among 4551 *E* gene sequences, 2319 belonged to the cosmopolitan genotype. These 2319 sequences and 1048 newly generated *E* gene sequences were used to select sequences for the phylogeography and lineage analyses. The third dataset included complete genomes (n = 1548) of DENV-2 retrieved from the NCBI nucleotide repository, and 158 newly generated complete genomes of cosmopolitan genotype. This dataset was used to select sequences for the complete genome-based Bayesian phylogeny and mutation profiling analyses. Sequences of each dataset were aligned by using the MAFFT software^36^.

### Accordance to relevant guidelines

All DENV-positive patient sera used for the generation of new *E* gene sequences (n = 915) were collected after obtaining the written informed consent from respective patients. Informed consent was obtained from the parents/legally authorized representatives of subjects that are under 21. All sera were utilized in accordance with the guidelines approved by the Institutional Review Board of National Environmental Agency, Singapore (IRB003.1).

### Ethical approval

The study was approved by the Institutional Review Board of National Environmental Agency, Singapore (IRB003.1).

### Sequencing of DENV-2 envelope gene and complete genomes

Envelope gene sequences (n = 1048) were generated directly from patient sera and mosquito homogenates. Complete genomes (n = 158) were generated from isolates obtained from patient sera. DENV was isolated from sera using the *Ae. albopictus* C6/36 mosquito cell line (ATCC CRL-1660) as described in detail elsewhere^37^. Viral RNA was extracted from sera using the QIAGEN QIAamp viral RNA mini kit (QIAGEN, Hilden, Germany) according to manufacturer’s recommendations. Viral RNA from mosquitoes was extracted as described previously^38^. DENV-2 typing, synthesis of complementary DNA (cDNA) and *E* gene and complete genome sequencing were done as described elsewhere^34,37^. Amplification and sequencing primers used are given in Supplementary Table S2. Raw nucleotide sequences were assembled using the Lasergene package version 15.0 (DNASTAR Inc., Madison, WI, USA).

### Spatial distribution of DENV-2 genotypes

Information pertaining to the country of origin (location) and year of isolation (time) of each sequence was extracted from NCBI flat files using a python script. Sylvatic genotype was excluded from the analyses because of extremely low numbers reported and its negligible contribution to global dengue burden. All *E* gene sequences (n = 4551) were categorized into known genotypes based on a neighbor-joining phylogenetic tree constructed by using the maximum-likelihood composite model in MEGA7 software suite^39^. A database (.csv) was prepared for each genotype to include the corresponding country of origin as well as latitude and longitude information of the capital city of each country. To determine the extent of global presence, countries that have reported each genotype, except the American genotype, were ranked into > 90th, 75–90th, 60–74th, 30–59th and < 30th percentile categories based on the number of sequences reported. Due to low sample size, countries that have reported the American genotype were categorized into > 90th, > 89–80th, > 79–70th, > 69–60th and < 60th percentiles for better visualization. The data was plotted onto the global map by using a custom made python v3.6 script built on cartopy.mpl.gridliner map builder module that was run on Spyder 4.1.5 software integrated into the open source Anaconda Navigator (https://docs.anaconda.com/anaconda/install/windows/).

### Bayesian phylogeny of DENV-2 complete genomes

Bayesian phylogeny analysis was conducted to determine the evolutionary characteristics of DENV-2 genotypes using 419 complete genome sequences. The analysis included all cosmopolitan genotype sequences (n = 380) and subsets of Asian I (n = 10), Asian II (n = 10), Asian-American (n = 8), American (n = 8) and sylvatic (n = 3) genotypes. The sequences of “non-cosmopolitan” genotypes were selected to represent the time and diversity span of each genotype determined based on a neighbor-joining tree constructed in MEGA7 software suite^39^. Each sequence was time tagged based on the year of isolation information. The Bayesian phylogeny was constructed by using general time reversible substitution model with gamma distribution and invariant sites (GTR + Г5 + I), codon partition (1 + 2) and 3, uncorrelated log-normal relaxed molecular clock model and Bayesian skyline prior (stepwise, 10 bins) in BEAST v1.10.4 software suite^40,41^. The best fitting nucleotide model for the analyzed dataset was determined by using jModeltest 2.1.5^42^. The BEAST run included 400 million Markov Chain Monte Carlo (MCMC) chains that were sampled at every 40,000 generations to ensure convergence at a minimum effective sample size (ESS) of > 200. The MCMC output was analysed using Tracer v.1.6 software (available at http://beast.bio.ed.ac.uk/Tracer). The maximum clade credibility (MCC) tree was constructed using TreeAnnotator v.1.10.4 software and visualized in FigTree v.1.4.4 software (available at http://tree.bio.ed.ac.uk/software/figtree/). Evolutionary parameters such as evolutionary rates and time to most recent common ancestor (tMRCA) were calculated based on the MCC tree. The uncertainty in parameter estimates was expressed as 95% highest posterior density (HPD).

### Discrete phylogeography analysis

Dispersal pattern of DENV-2 cosmopolitan genotype was determined by using the Bayesian Stochastic Search Variable studies (BSSVS) analysis^43^. Each country was treated as a discrete trait in the analysis. To minimize the redundancy and computational resources required, identical sequences reported from each country in a particular year were removed from the original *E* gene alignment. The final alignment included 929 *E* gene sequences, reported from 27 countries. BSSVS analysis was conducted by using GTR + Г5 + I as the best-fitting substitution model based on jModelTest output^42^, Bayesian skyline demographic prior with default 10 bins and a relaxed uncorrelated lognormal clock^44^ in a MCMC run of 100 million generations, sampling every 10,000 states. Phylogeographic reconstructions and Bayes factor (BF) calculations were done in SPREAD 1.0.4 software^45^ by comparing the posterior and prior probability of individual rates to test the significant linkage between locations. Links with BF values > 3 and posterior probability ≥ 0.75 were considered well-supported. Well-supported links were categorized into very strong (BF > 30) and decisive (BF > 100)^43,46^.

### Envelope gene phylogeny of cosmopolitan genotype sequences

The neighbour joining tree was constructed by using 3367 complete *E* gene sequences. The alignment included 2319 sequences retrieved from NCBI database and 1048 sequences generated during the present study. The analysis was done by using the kimura-2 parameter substitution model with gamma parameter and invariant sites (Г5 + I).

### Mutation profiling of cosmopolitan genotype sequences

Unique mutations of the cosmopolitan genotype were identified by using an alignment of 1548 complete coding sequences of DENV-2 retrieved from NCBI nucleotide repository. The analyses were done in Jalview 2.11.1.3 software^47^. Each sequence was categorized into a known genotype based on phylogenetic clustering. Firstly, residues that were conserved in > 96% of cosmopolitan genotype sequences, but different in other genotypes of DENV-2 were identified as unique substitutions of the cosmopolitan genotype. Secondly, the analysis zoomed into cosmopolitan genotypes sequences to identify unique substitutions of the Indian sub-continent lineage as compared to other lineages in the same manner. Each mutation signature comprised the combination of all such unique substitutions.

### Genetic distance analyses of DENV-2 sequences

The overall nucleotide similarity and pairwise nucleotide diversity (π index) were determined between the cosmopolitan genotype (Indian sub-continent and other lineages) and other genotypes of DENV-2 using the complete *E* gene (1485 bp) alignment. The overall nucleotide similarity among different categories/genotypes were calculated in BioEdit version 7.2.5^48^. The π index is defined as the number of nucleotide differences between two sequences per base pair^49^. The π index was determined in Biopython^50^ by estimating the pairwise nucleotide diversity of each pair of sequences calculated with Dendropy^51^, considering cosmopolitan (other lineage) sequences as the reference dataset.

### Selection pressure analyses

The selection pressure acting on each codon of complete coding sequences of 488 (99 Indian sub-continent lineage and 389 other lineages) were measured by the ratio of non-synonymous to synonymous rates computed in HyPhy open-source software package as implemented in Datamonkey web-server^52^. Single likelihood ancestor counting (SLAC), fixed effects likelihood (FEL), mixed effect model of evolution (MEME) and fast unbiased Bayesian approximation (FUBAR) methods were used to estimate the sites under selection. The GTR nucleotide substitution bias model and neighbor joining phylogeny were used as analytical parameters in different approaches. An integrative approach recommended previously^52^ was employed in ascertaining the positive or negative selection at each site. The sites which were found to be non-neutral and be positively or negatively selected by at least three methods at significant levels were considered to be positively/negatively selected sites. The p-values ≤ 0.1 were considered as significant for SLAC, FEL and MEME methods, whereas the Bayes factor ≥ 30 was used as the significance threshold for FUBAR method.

## Results and discussion

### Cosmopolitan genotype demonstrated inter-continental dispersal through an extensive spatial network

**Figure 1:**
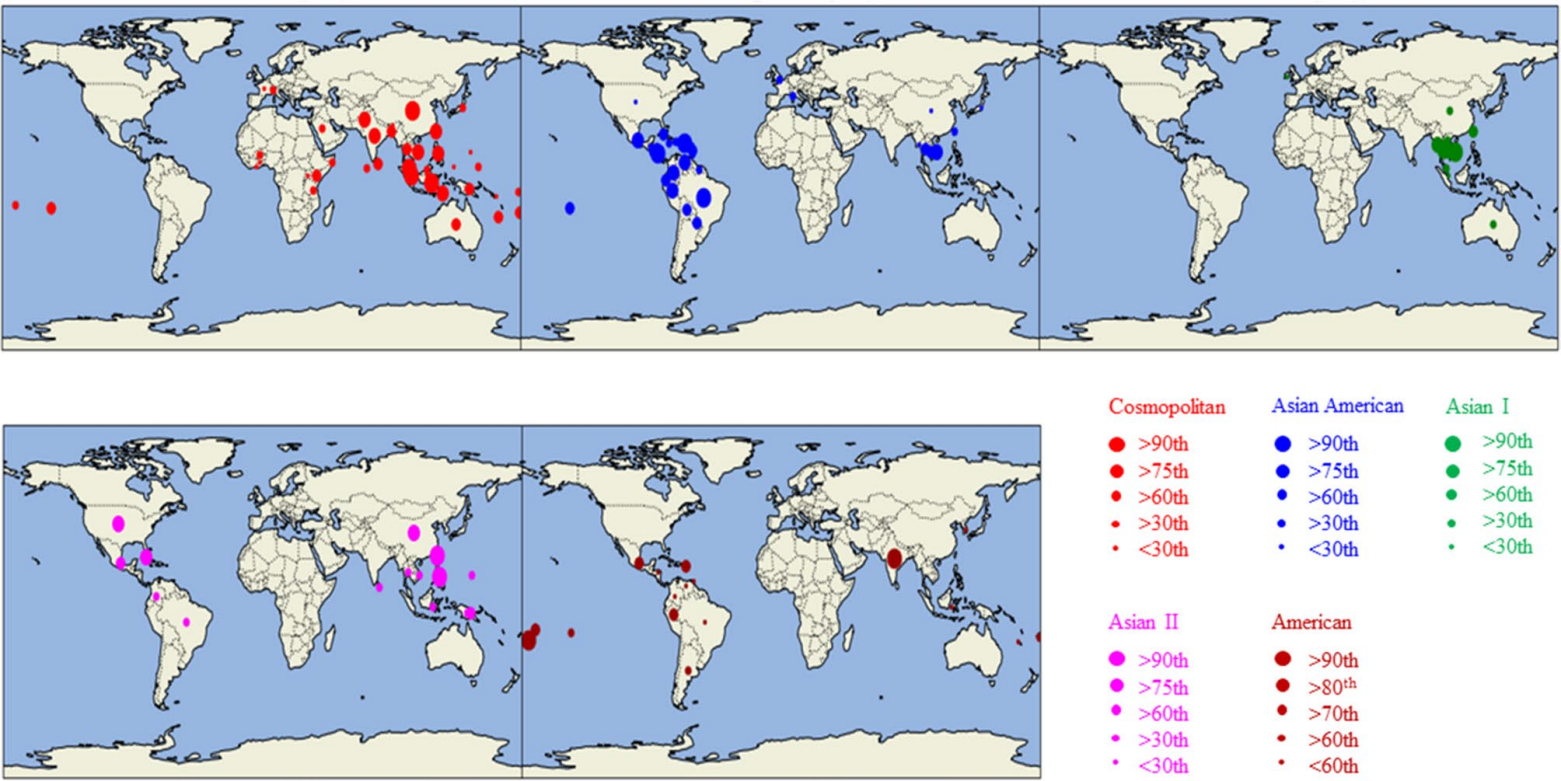
Spatial distribution of DENV-2 genotypes. Genotypes are represented by different colors as shown in the legend. The number of sequences belonging to each genotype reported by all countries was ranked into > 90th, 76–90th, 60–74th, 30–59th and < 30th percentile categories that are shown in circles of different size. Due to low sample size, the percentile ranking for American genotype was done at > 90th, > 89–80th, > 79–70th, > 69–60th and < 60th percentiles for better visualization. The data was plotted onto the global map by using a custom made python v3.6 script built on cartopy.mpl.gridliner map builder module that was run on Spyder 4.1.5 software integrated into the open source Anaconda Navigator (https://docs.anaconda.com/anaconda/install/windows/). Based on the data available in NCBI database, the period of sampling for sequencing was from 1944 to 2019.

A spatial analysis of DENV-2 sequences of all known genotypes, except the sylvatic genotype, was conducted to determine their distribution patterns. All complete *E* gene sequences (1485 bp) of DENV-2 as of July 2019 were retrieved from the NCBI database and tagged with the country of origin (location) and year of isolation (time) information available in NCBI flat files. The final alignment included 4551 sequences. Each sequence was classified into a known genotype, based on the phylogenetic relationship. Sequences of each genotype were mapped to determine the global distribution (Fig. 1). The analysis demonstrated a variable degree of geo-distribution of each genotype. Cosmopolitan genotype was the most widely distributed, though was remarkably absent in the Americas. Asian II and Asian-American genotypes were present in Southeast Asia as well as Central and South America. American genotype was the least widespread. Asian I genotype was particularly restricted to Southeast Asia. Southeast Asia showed the highest genotype heterogeneity and was the most notable global epicenter of DENV-2 transmission.

**Figure 2:**
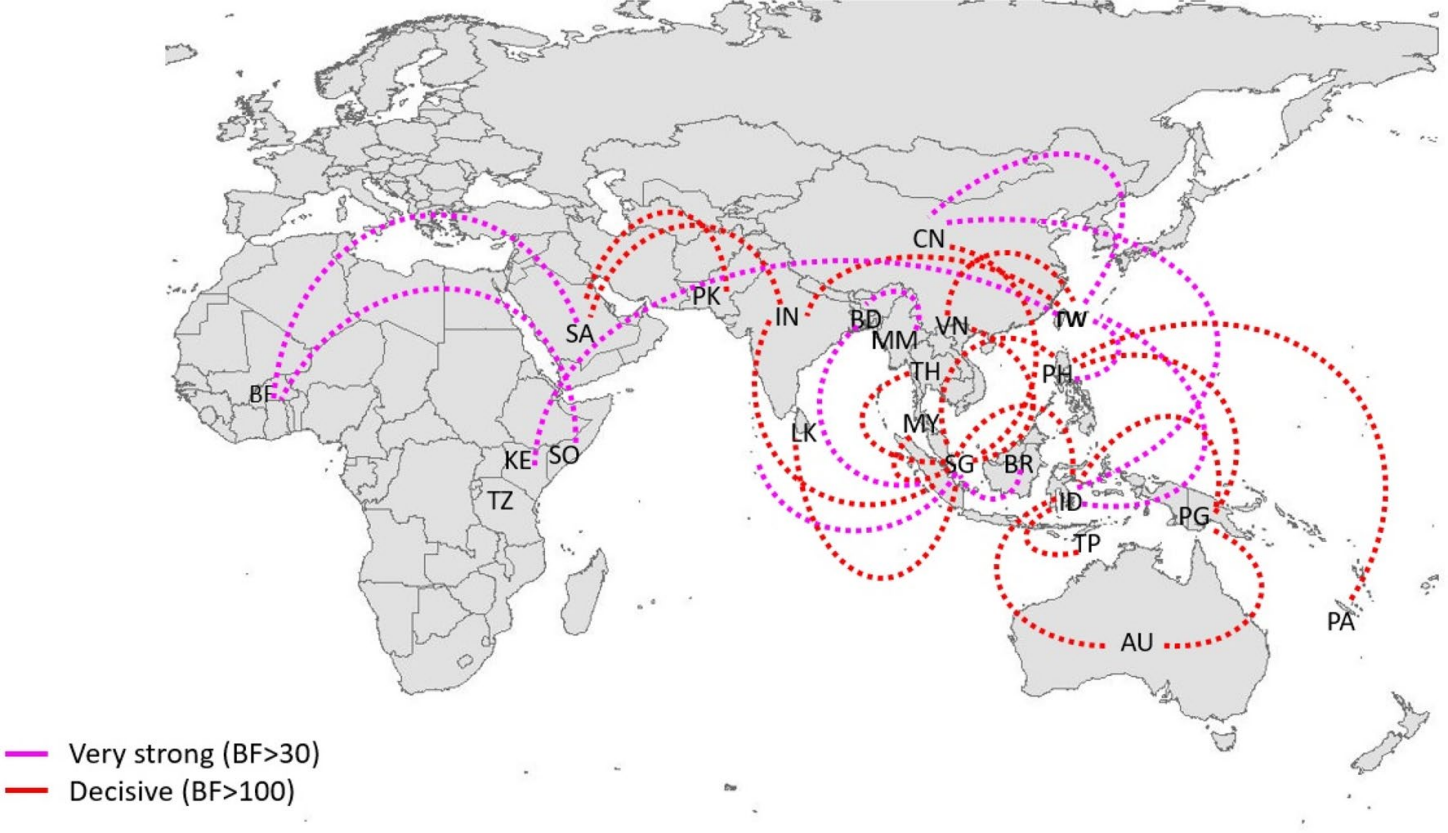
Dispersal pattern of DENV-2 cosmopolitan genotype. The spatio-temporal dispersal pattern was determined by using Bayesian Stochastic Search Variable studies (BSSVS) analyses of 929 complete *E* gene sequences. The analysis demonstrates dispersal links of cosmopolitan genotype. The map was created by using the ggplot2 package in R 3.6.3 software^53^ and the links were manually created in Microsoft PowerPoint version 2008. Only the links with Bayes Factor (BF) > 3 and posterior probability ≥ 0.75 are shown. The strength of links was categorized into very strong (BF > 30) and decisive (BF > 100) links based on the BF values. Each country was represented by a two letter code. *AU* Australia, *BD* Bangladesh, *BF* Burkina Faso, *BR* Borneo, *CN* China, *IN* India, *ID* Indonesia, *KE* Kenya, *MM* Myanmar, *MY* Malaysia, *PA* Pacific Islands, *PK* Pakistan, *PG* Papua New Guinea, *PH* Philippines, *SA* Saudi Arabia, *SG* Singapore, *LK* Sri Lanka, *TH* Thailand, *TP* East Timor, *TW* Taiwan, *TZ* Tanzania, *VN* Vietnam.

Bayesian Stochastic Search Variable studies (BSSVS)-based phylogeography analyses were conducted to determine the spatio-temporal dispersal and the most active hubs of cosmopolitan genotype (Fig. 2). The analyses showed an extensive dispersal network of short- and long-distance links. All BSSVS links achieved high statistical support (Bayes Factor > 3 and posterior probability ≥ 0.75). Inter-continental dispersal was notable among the long-distance links.

The BSSVS findings reflected the epidemiological reality of cosmopolitan genotype’s presence in a wide geographical scale^20,33,54–61^. The most intense virus activity was visible in Southeast Asia, mainly in two epicenters; first, an area covering Singapore, Malaysia and Indonesia and the second centered on Taiwan and China in the Far East. Both epicenters actively exchanged virus strains with Australasia and the Pacific regions. The Far East hub also demonstrated long-range dispersal links with East Africa. However, both hubs were not linked to the Middle East. Instead, Middle East demonstrated alternative dispersal links with the Indian sub-continent. Indian sub-continent also showed decisive links with Southeast Asian and Far-East epicenters, suggesting active virus exchange between the sub-continent and other regions. The extensive network demonstrated how the global trade and travel have facilitated the geo expansion of DENV-2 cosmopolitan genotype. The long-range dispersal of DENV is possible either through the migration of humans, infected mosquitoes or desiccated mosquito eggs. Human migration is unarguably the more likely mode of distant virus dispersal than live mosquito transfer. Virus dispersal through desiccated mosquito eggs is less plausible than other modes due to limited evidence of vertical transmission of DENV^62^. The rapid expansion of air travel in recent decades has immensely facilitated human migration and thereby DENV dispersal^63^. For instance, Singapore is one of the most connected global aviation hubs and showed numerous inbound and outbound dispersal links in our BSSVS analysis (Fig. 1). China’s economic and trade expansion resonates well with its long-distance outbound virus dispersal to the African continent. The substantial dispersal of DENV-2 from Indian sub-continent to the Middle East might be due to increased trade and pilgrimage travel^64^.

Overall the extensive dispersal of cosmopolitan genotype suggests its ability to adapt to vector and host populations in a wide geographical range. This contrasts with DENV-2 genotypes such as American and Asian I, which are largely restricted to certain geographies^5^. However, cosmopolitan genotype seemed to have struggled to establish in locations where these geographically restricted genotypes dominated. For example, of 4,551 *E* gene sequences analyses in the present study, Asian I genotype (70.3%, n = 333) was the most common among 474 sequences from Vietnam. This was in comparison to 7.6% (n = 33) of cosmopolitan genotype sequences. The same pattern was observed among 322 sequences from Thailand (91.3% Asian I; n = 294 and 5.9% cosmopolitan; n = 19). Therefore, the ability of cosmopolitan genotype to survive in different environments seemed to be variable. One of the contributory factors for this variability might be evolutionary adaptations that generate heterogeneity, confer fitness advantages, and cause selective sweeps, allowing virus lineages to dominate differentially in various geographies^65^. This warranted further analyses of evolutionary signatures, heterogeneity, sub-lineage geo-clustering and natural selection of the cosmopolitan genotype.

### Cosmopolitan genotype demonstrated high intra-genotype heterogeneity

**Figure 3:**
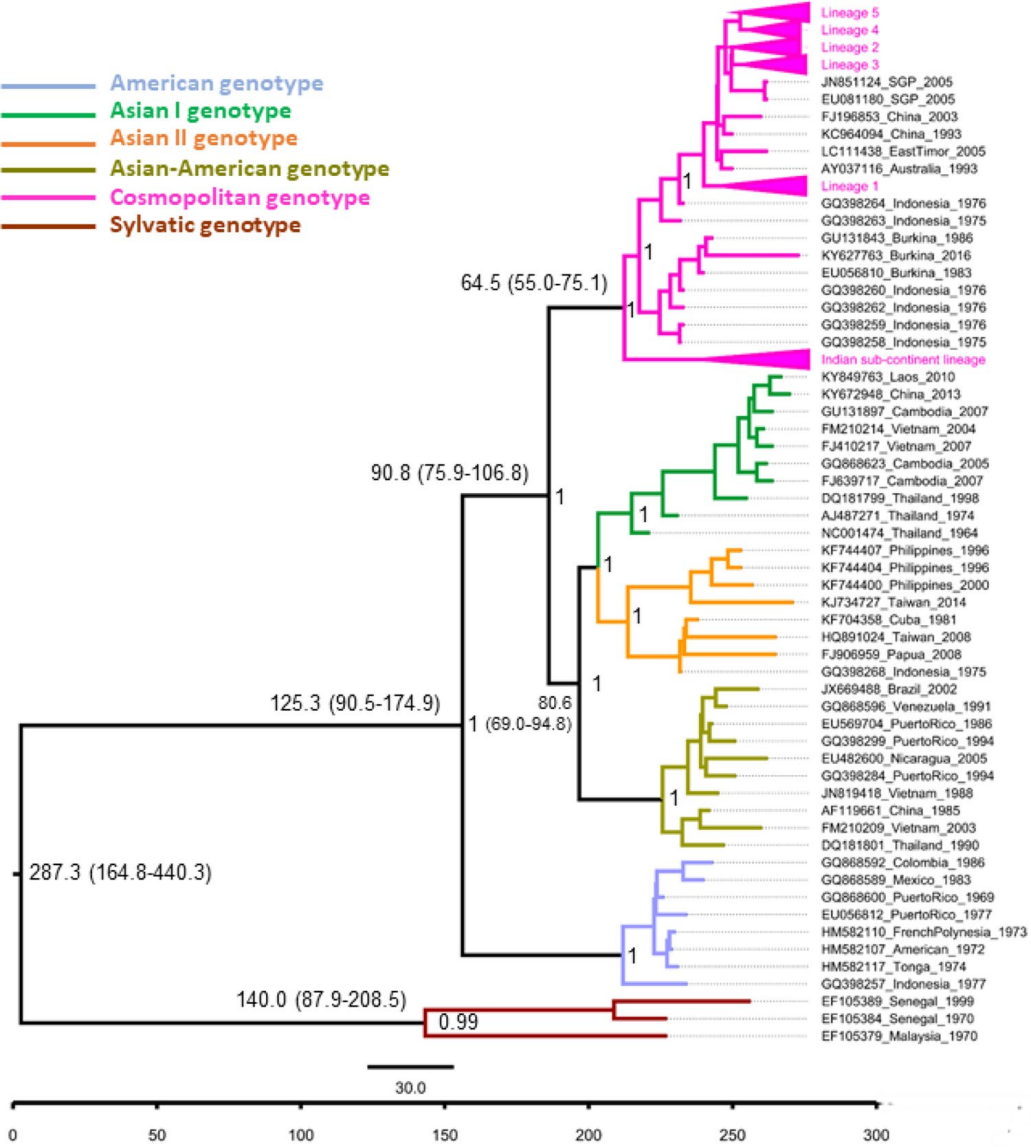
Bayesian phylogeny of DENV-2 complete coding sequences. The maximum clade credibility (MCC) tree was constructed in BEAST 1.10.4 version, using general time reversible (GTR) substitution model, relaxed random clock model and Bayesian skyline prior. The analysis included 380 complete coding sequences of the cosmopolitan genotype and a relatively small reference dataset to represent Asian I (n = 10), Asian II (n = 10), Asian-American (n = 8), American (n = 8) and sylvatic (n = 3) genotypes. Each genotype is colour coded as shown in the legend. The numbers on branches represent median node heights in years. The 95% highest posterior density values for node heights are given in brackets. The numbers at the nodes represent the posterior probability values. The time scale (in years) and scale bar (substitutions/site/year) are given at the bottom of the figure. An expanded view of the tree is given in Supplementary Fig. S2.

The Bayesian phylogeny of DENV-2 complete coding sequences revealed distinct clustering of each genotype, with strong posterior probability support (Fig. 3). The cosmopolitan genotype comprised of two distinct groups. One group included sequences belonging to the Indian sub-continent lineage, which has largely been reported in the Indian sub-continent^66,67^. The other group included the rest of cosmopolitan genotype sequences reported from other countries, especially in Southeast Asia.

The mean nucleotide substitution rate of each genotype ranged from 10^–4^ to 10^–3^ subs/site/year. This estimate agreed with the empirical data^64,66,68^, demonstrating comparable evolutionary rates among all genotypes of DENV-2, regardless of their differences in distribution. The inferred mean root age of DENV-2 was ~ 287.3 years from 2019 (95% highest posterior density (HPD)—164.8 to 440.3 years), which was comparable to previous estimates of 325.2 years^64^. The tMRCA data also suggested that cosmopolitan genotype might have emerged in parallel to Asian I and II genotypes between 1944 and 1970, based on 95% HPD values. The analysis predicted a major split in cosmopolitan genotype, with the emergence of Indian sub-continent lineage in early 1980s.

The cosmopolitan genotype was distinct from other genotypes by a 12-amino acid signature (Table 1). Among non-sylvatic genotypes of DENV-2, each residue of this signature was “fixed” and unique to the cosmopolitan genotype. The signature mutations were spread across the structural and non-structural proteins (PreM/M, Envelope, NS3, NS4B and NS5), implying a genome-wide selection of amino acid residues during the evolutionary history of cosmopolitan genotype. Whether these residues conferred a fitness advantage to cosmopolitan genotype warrant further investigations.

**Table 1:** Unique amino acid signature of cosmopolitan genotype.

Polyprotein position	Gene	Gene position	REF	Other genotypes	Cosmopolitan genotype (% of fixation^a^)
166	preM/M	52	K	K	N (100%)
196	preM/M	82	T	T	A (99%)
262	preM/M	148	H	H	Y (96%)
351	E	71	E/D	E/D	A (100%)
429	E	149	H	H	N (99%)
670	E	390	N/D	N/D	S (97%)
742	E	462	I	I	V (99%)
1635	NS3	160	A/S	A/S	T (100%)
2256	NS4B	13	L	L	F (99%)
2496	NS5	5	I/V/M	I/V/M	T (99%)
2506	NS5	15	S	S	N (100%)
2826	NS5	335	V/L	V/L	I (100%)

^a^The percentage values indicate the extent of fixation of each mutation in 445 sequences of cosmopolitan genotype as compared to 1,007 sequences of other genotypes of DENV-2. NCBI reference sequence NC_001474.2 was used as the reference (REF) genome. *M* membrane, *NS* non-structural proteins, *preM* pre-membrane.

A separate phylogenetic analysis was performed by using complete *E* gene sequences to determine the intra-genotype heterogeneity of cosmopolitan genotype. Here, *E* gene was preferred to complete genomes because of the availability of more *E* gene sequences from different geographical regions. *E* gene is also a widely used candidate for the phylogenetic classification of DENV lineages^13,69,70^. The analysis included 3,367 complete *E* gene sequences of cosmopolitan genotype reported from 41 countries (Supplementary Table S3).

**Figure 4:**
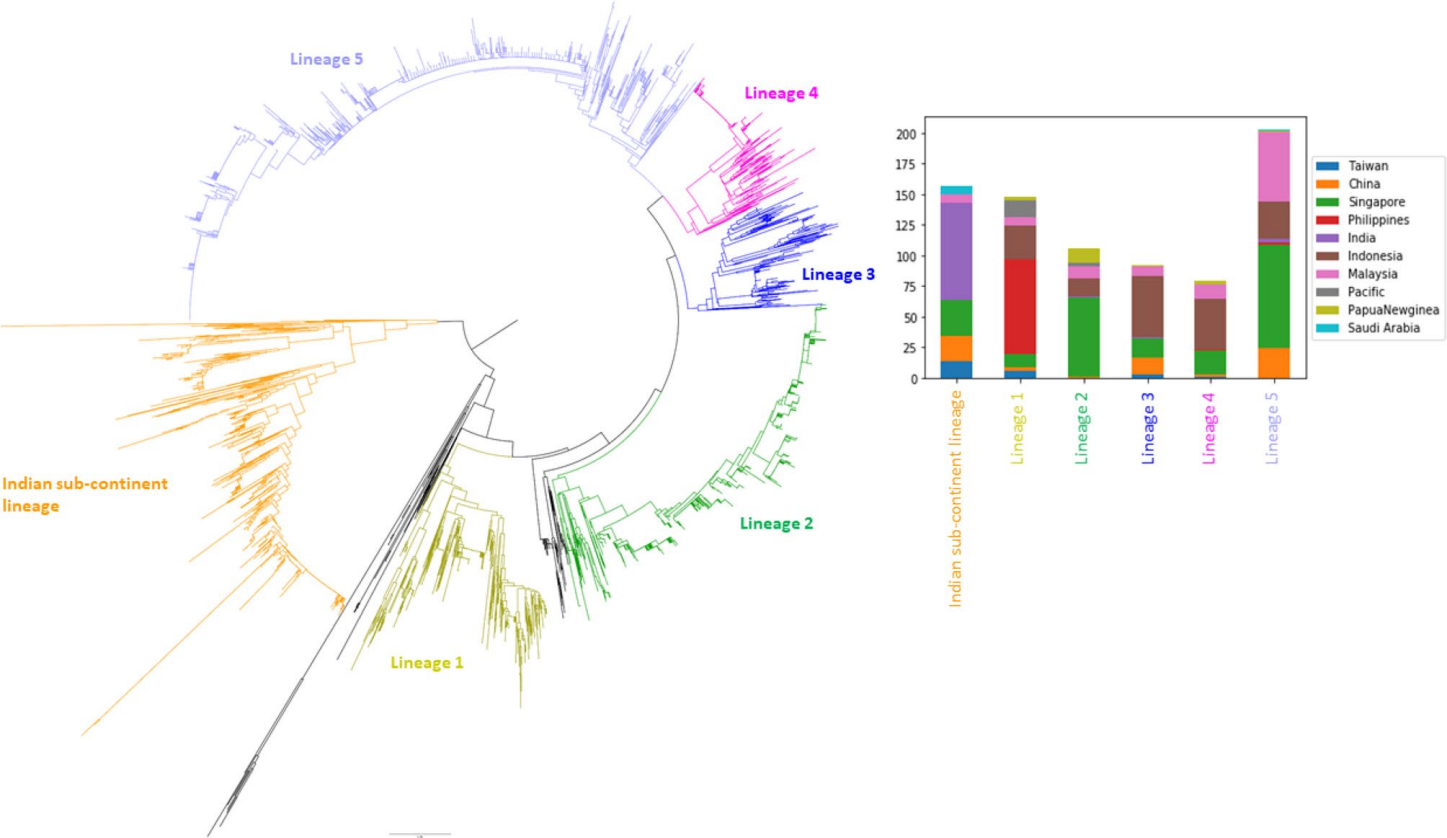
Phylogenetic analysis of *E* gene sequences of cosmopolitan genotype. The neighbour joining tree was constructed by using 3367 complete *E* gene sequences. The alignment included 2319 sequences retrieved from NCBI database and 1048 sequences generated during the present study. Distinct lineages are shown in different colours. Sequences that were not classified into any distinct lineage are shown in black branches. An expanded tree that illustrates the distribution of newly generated sequences obtained from human sera and field-caught mosquitoes is given in Supplementary Fig S3. The inset bar chart represents geographical distribution of each lineage.

Similar to the whole genome-based phylogeny, *E* gene tree also branched into two main groups at the root, separating the Indian sub-continent lineage from the rest of cosmopolitan genotype (other lineages) sequences (Fig. 4). Besides, other lineage sequences clustered into five distinct lineages, suggesting an overall high heterogeneity of the cosmopolitan genotype (Fig. 4). Based on the nucleotide and amino acid similarity matrices (Table 2), Indian sub-continent lineage was the most divergent among all lineages within the cosmopolitan genotype.

**Table 2:** *E* gene nucleotide similarity matrix between different lineages of the cosmopolitan genotype.

Lineage	Other lineages					Indian sub-continent lineage
	Lineage-1	Lineage-2	Lineage-3	Lineage-4	Lineage-5	
Lineage-1	1.00	0.97	0.97	0.97	0.97	0.94
Lineage-2	0.97	1.00	0.97	0.97	0.96	0.93
Lineage-3	0.97	0.97	1.00	0.97	0.97	0.93
Lineage-4	0.97	0.97	0.97	1.00	0.98	0.94
Lineage-5	0.97	0.96	0.97	0.98	1.00	0.94
Indian sub-continent lineage	0.94	0.93	0.93	0.94	0.94	1.00

The geographical distribution of these lineages varied within the complete dataset. Notably, sequences from Indonesia positioned at the base of all lineages, except the Indian sub-continent lineage. On the other hand, sequences from Singapore, Malaysia and China appeared in all lineages. While all lineages were spread across different countries, certain lineages dominated in a particular country or a region. For example, lineage 1 almost entirely comprised sequences from the Philippines. There were two outgroups of lineage 1 that included sequences from the Pacific Islands (founder outgroup) and Indonesia (secondary outgroup), suggesting a likelihood of the earliest ancestry of lineage 1 either in the Pacific Islands or Indonesia, and subsequent introduction into the Philippines. Interestingly, the phylogeography analysis showed a decisive direct link between the Philippines and the Pacific, and an indirect decisive link with Indonesia, through Papua New Guinea (Fig. 2). Root state posterior probability data extracted from the MCC tree suggested that the most likely common ancestor of lineage 1 originated in Indonesia. This strain might have spread to the Philippines via Papua New Guinea as shown in the phylogeography analysis (Fig. 2). A similar observation was also made in the Indian sub-continent lineage that is largely circulating in the Indian sub-continent countries. The earliest available sequence in the basal clade of this lineage (outgroup) was a sequence reported from India in 1974 (NCBI Accession FJ538920). Two sub-lineages (sub-lineage 1 and 2) emerged from this outgroup in early 1990s. One of them showed limited evidence of outbound spread from the Indian sub-continent, mainly to the Middle East and East Africa. In contrast, the other sub-lineage showed relatively wide dispersal from the Middle East to Southeast and East Asia. The available data showed records of both sub-lineages as late as 2017–2018.

### Selection pressures acted on different amino acid residues between Indian sub-continent and other lineages of cosmopolitan genotype

The spatio-temporal dominance of virus genotypes is influenced by selective sweeps that partly occur during virus-host adaptations. In arboviruses, such as DENV-2, these adaptations occur in both vertebrate and invertebrate hosts^71^. Therefore, we hypothesized that selection pressure analyses among different lineages would depict amino acid residues that altered during evolution and dispersal of the cosmopolitan genotype. To achieve this, differential rates of nonsynonymous and synonymous substitution rates per site were estimated in the complete coding sequences of cosmopolitan lineages (447 sequences of other lineages and 98 sequences of Indian sub-continent lineage) using SLAC, FEL, FUBAR and MEME methods^72–75^. The findings showed that majority of residues were neutral and purifying selection was more pronounced than the positive selection. This was in agreement with previous studies^72,76^. Among the few positively-selected sites, there was no overlap between the Indian sub-continent lineage and other lineages, suggesting differences in selective pressures acting on these lineages. NS2A-33 residue showed evidence of positive selection in the Indian sub-continent lineage. Valine was present at residue NS2A-33 in 77% of Indian sub-continent lineage sequences, whereas Isoleucine was present at the same position of other lineages. On the other hand, the positive selection was evident on C-9R, NS2A-171A, and NS3-392S residues in other lineages of the cosmopolitan genotype (Table 3).

**Table 3:** Selection pressure analysis of the complete coding region of cosmopolitan genotype.

Lineage	Gene	Gene position	SLAC	FEL	FUBAR	MEME
Lineages 1–5	**C**	**9**	**0.05**	**0.03**	**89.45**	0.67
	C	95	0.14	0.09	46.73	0.11
	E	347	0.26	0.22	29.06	0.07
	E	390	0.11	0.02	214.78	0.67
	NS1	178	0.18	0.16	48.62	0.16
	**NS2A**	**171**	**0.02**	**0.03**	**88.23**	**0.04**
	**NS3**	**392**	**0.04**	**0.03**	**134.29**	**0.02**
	NS5	648	0.03	0.23	80.97	0.14
Indian sub-continent lineage	preM	16	0.34	0.18	9.69	0.2
	preM	57	0.41	0.19	10.86	0.1
	E	52	0.28	0.14	14.86	0
	E	360	0.29	0.14	20.05	0.67
	NS1	192	0.34	0.19	9.61	0.01
	**NS2A**	**33**	**0.07**	**0.06**	**65.28**	0.67
	NS5	631	0.16	0.04	91.78	0.67

Positive selection was evident on residues in bold font, based on the selection pressure analyses. The values shown for each method are p-values, except for the FUBAR method for which the values indicate the Bayes factor. The p-values ≤ 0.1 were considered as significant for SLAC, FEL, and MEME methods, whereas the Bayes factor ≥ 30 was used for FUBAR method. *C* Capsid, *E* envelope, *NS* non-structural proteins.

### Is Indian sub-continent lineage becoming a new genotype?

Having been genetically, evolutionarily and geographically distinct from the rest of cosmopolitan lineages (Fig. 4), we investigated whether the Indian sub-continent lineage is divergent enough to be classified as a new genotype. To achieve this, we analyzed the genetic divergence of Indian sub-continent lineage from other known non-sylvatic genotypes (Asian I, Asian II, Asian-American and American genotypes). These analyses included complete *E* gene sequences of all cosmopolitan genotype sequences used in the current study, but split into two categories, namely “Indian sub-continent lineage” and “other lineages”. Other lineages category included lineages 1–5 illustrated in Fig. 4. The dataset also included sequences of Asian I, Asian II, Asian-American and American genotypes that were selected to represent the genetic and geographical variations within each genotype. First, *E* gene nucleotide and amino acid similarity scores were calculated and compared between the Indian sub-continent and other lineages of cosmopolitan genotype and other genotypes (Table 4).

**Table 4:** Comparison of *E* gene divergence matrices between the Indian sub-continent lineage and other lineages of cosmopolitan genotype and other genotypes of DENV-2.

Genotype	Nucleotide dissimilarity (Amino acid dissimilarity)	
	Cosmopolitan (other lineages)	Cosmopolitan (Indian sub-continent lineage)
Cosmopolitan (other lineages)	0% (0%)	5.6% (5.3%)
Cosmopolitan (Indian sub-continent lineage)	5.6% (5.3%)	0% (0%)
Asian II	8.8% (7.9%)	8.6% (7.4%)
Asian I	8.8% (8.8%)	8.9% (8.2%)
American	10.2% (10%)	9.4% (9%)
Asian/American	7.8% (8.5%)	7.1% (7.7%)

**Figure 5:**
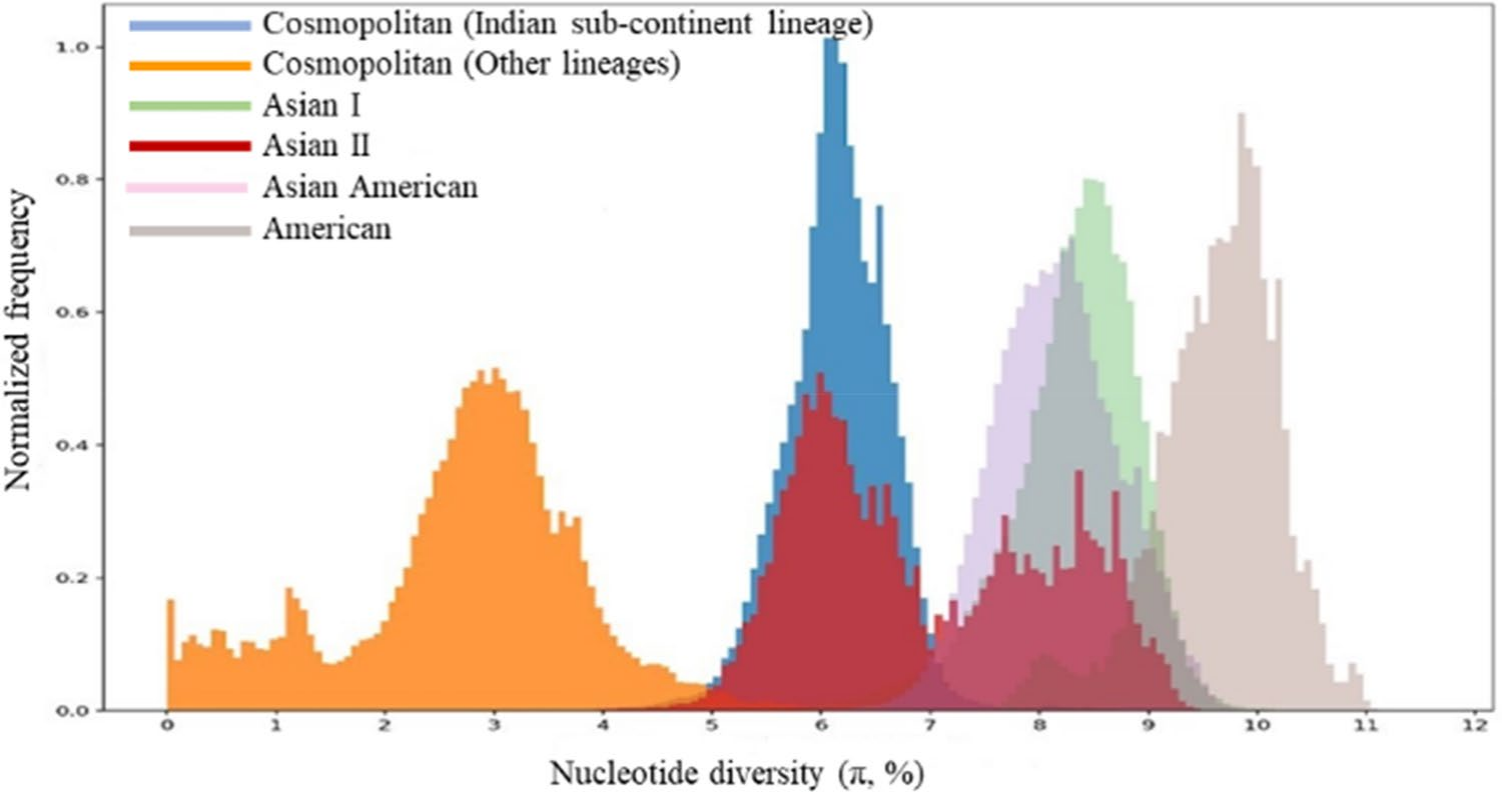
Pairwise nucleotide diversity (π index) comparison between cosmopolitan (Indian sub-continent lineage and other lineages) and other known genotypes of DENV-2. Each colour represents a different genotype of DENV-2.

The nucleotide dissimilarity between the cosmopolitan (other lineages) and Indian sub-continent lineage was 5.6% (Table 4). Respective values between the cosmopolitan (other lineages) and other genotypes ranged between 7.8% (Asian-American) and 10.2% (American). The Indian sub-continent lineage also demonstrated comparable nucleotide divergence with other genotypes of DENV-2 (divergence range: 7.1%-9.4%). Empirical data has suggested a nucleotide divergence (range 3–6%) threshold of 6% within *E* gene among different genotypes of DENV-2^7,14,77^. Our data showed that nucleotide divergence between the cosmopolitan (other lineages) and Indian sub-continent lineage (5.6%) is on the verge of this threshold. To further corroborate the genetic distinction between Indian sub-continent and other lineages of cosmopolitan genotype, we compared π nucleotide diversity index values of *E* gene sequences of cosmopolitan genotype (split into Indian sub-continent and other lineages) and other genotypes of DENV-2. As shown in Fig. 5, cosmopolitan genotype demonstrated the highest nucleotide diversity among all non-sylvatic genotypes of DENV-2. Moreover, Indian sub-continent and other lineages formed distinct subpopulations within the cosmopolitan genotype, with ~ 6% *E* gene pairwise distance between the two groups. The π index of Indian sub-continent lineage overlapped with a batch of Asian II genotype sequences, further corroborating the genetic distinction of Indian sub-continent lineage from other lineages of cosmopolitan genotype. These observations suggested that Indian sub-continent lineage has already diverged from the rest of cosmopolitan genotype lineages and is almost at the threshold of 6% nucleotide distance within *E* gene to qualify as a separate genotype.

**Figure 6:**
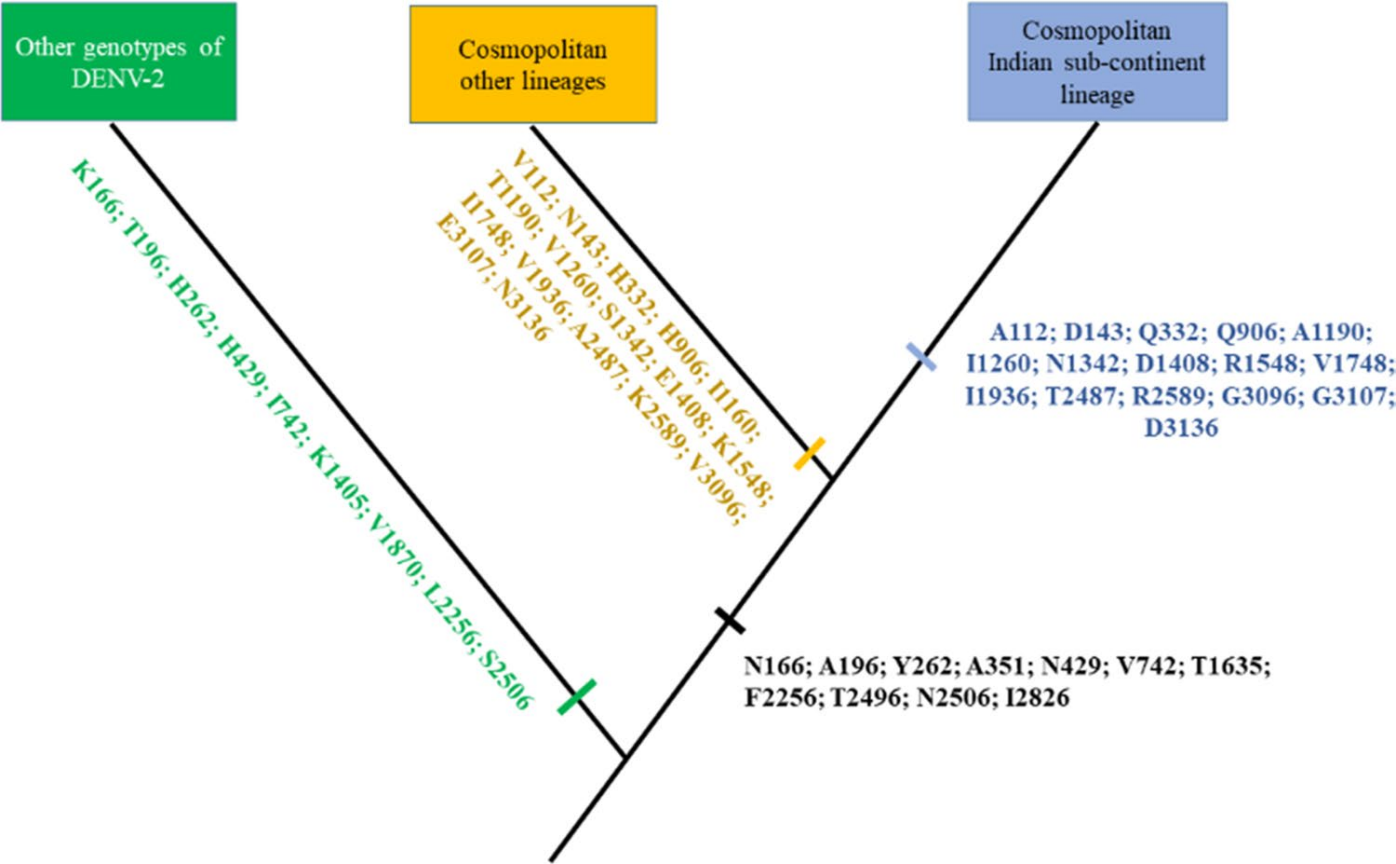
Schematic representation of unique amino acid signatures between Indian sub-continent and other lineages of cosmopolitan genotype. Amino acids are shown in the single letter code, with corresponding polyprotein position.

To further support the genetic distinction of Indian sub-continent lineage within cosmopolitan genotype, we compared complete coding sequences of Indian sub-continent and other lineages. The analysis showed a 16-amino acid signature unique to the Indian sub-continent lineage (Fig. 6, Supplementary Table S4).

Eight amino acid residues of this signature were also shared by a small subset of Indonesian sequences of the cosmopolitan genotype, isolated during 1974–1975 (Table 5). Based on NCBI data (latest by July 2019), the earliest sequence of Indian sub-continent lineage is a strain (NIV_742295) isolated in 1974 (NCBI Accession FJ538920). NCBI Accession FJ538920 is a partial genome sequence and was thus not included in the whole genome based tMRCA analysis that estimated the origin of Indian sub-continent lineage in early 1980s (Table 6). Therefore, the partial genome data suggested that Indian sub-continent lineage might have emerged even earlier than early 1980s. This was corroborated by the group of Indonesian sequences that were reported at the same time as the earliest genetic record of Indian sub-continent lineage in 1974. Sharing of amino acid residues unique to Indian sub-continent lineage by 1974–1975 Indonesian sequences also implied a common ancestry between the two groups. The earliest sequences of DENV-2 from Indonesia in 1974–1976 belong to the cosmopolitan genotype. The first reports of dengue in Indonesia was in 1968 in Jakarta and Surabaya^78^, suggesting that cosmopolitan genotype emerged during the early phase of dengue transmission in Indonesia. Based on the available literature, cosmopolitan genotype is the most common DENV-2 genotype, which has been associated with multiple outbreaks in the Indonesian archipelago^79–87^.

**Table 5:** Unique amino acid substitutions shared between the Indian sub-continent lineage and Indonesian sequences (1974–1976).

Polyprotein position	Gene	Gene position	REF	Cosmopolitan genotype		
				Indonesian (1974–1976; n = 07)	Indian sub-continent lineage (n = 94)	Other lineages (n = 344)
143	preM	29	D	D (86%), N (14%)	D (100%)	N (100%)
332	E	52	Q	Q (71%), H (29%)	Q (100%)	H (97%)
906	NS1	131	Q	Q (71%), H (29%)	Q (100%)	H (98%)
1408	NS2B	63	D	D (100%)	D (100%)	E (98%)
1748	NS3	273	V	V (100%)	V (100%)	I (98%)
1936	NS3	461	I	I (86%), V (14%)	I (98%)	V (97%)
2589	NS5	98	R	R (71%), K (29%)	R (98%)	K (98%)
3096	NS5	605	G	G (71%), V (29%)	G (100%)	V (98%)

Percentage values indicate the extent of fixation of each mutation in respective groups of sequences. NCBI reference sequence NC_001474.2 was used as the reference (REF) genome. *NS* non-structural proteins, *preM* pre-membrane.

**Table 6:** Time to most recent common ancestor (tMRCA) analysis of DENV-2 genotypes.

Description	Node height (95% HPD) in years	Estimated year of emergence^a^
Root ancestor	287.3 (164.8–440.3)	1732
Sylvatic	140 (87.9–208.5)	1879
Cosmopolitan genotype	64.5 (55–75.1)	1955
Cosmopolitan genotype (Indian sub-continent lineage)	36.3 (30.6–41.9)	1983
Asian II genotype	63.3 (49.1–77.6)	1956
Asian I genotype	62 (56.5–68.5)	1957
American genotype	65.3 (55.8–76.8)	1954
Asian-American genotype	51.1 (43.8–60.1)	1968

^a^Estimated year of emergence was calculated by subtracting the node height from 2019, which was the latest temporal data point in the analysis. The tMRCA data was obtained from the complete genome based maximum clade credibility tree. *HPD* Highest posterior density.

### Epidemiological significance of the Indian sub-continent lineage

Findings of the present study demonstrated that Indian sub-continent lineage is the most divergent among different lineages of the cosmopolitan genotype. In evolutionary terms, the extent of its divergence within the cosmopolitan genotype was remarkable and reached at levels comparable to other genotypes of DENV-2. Selection pressure analysis also corroborated the notion that Indian sub-continent lineage is likely to have evolved independently from the remaining lineages of DENV-2 cosmopolitan genotype. Moreover, findings demonstrated that the Indian sub-continent lineage is more widespread than other lineages of the cosmopolitan genotype. This indirectly indicates the adaptability of Indian sub-continent lineage to vectors and environment conditions in different geographies. This survivability suggests its association with outbreaks, whenever the ground conditions are ripe enough to establish transmission across a wide geo-scale, testifying the epidemiological success of Indian sub-continent lineage for approximately five decades. Findings of the present study suggested presence of its ancestral strains in India and Indonesia in early 1970s. However, isolates sequenced during Indonesian outbreaks after 1976 belong to lineages other than Indian sub-continent lineage, suggesting that the latter was unable to establish transmission in Indonesia, despite the sustained transmission of a diverse cosmopolitan genotype population in the archipelago.

DENV-2 was evident in India as early as 1956 based on the available sequence data^88^ and has historically been the predominant serotype associated with outbreaks in the country^89^. The earliest DENV-2 sequences from India belong to the American genotype, which was subsequently replaced by the cosmopolitan genotype in early 1970s^89^. Even the earliest cosmopolitan genotype sequences from India belong to the Indian sub-continent lineage^89^, and the same lineage dominated during subsequent outbreaks in different regions^66,90–94^, suggesting the important epidemiological role played by this lineage in India. Indian sub-continent lineage subsequently spread to other countries in Indian sub-continent, Southeast Asia, Middle East and Africa. Its expansion in the Indian sub-continent and Southeast Asia was evident during outbreaks in Bangladesh^95^, Bhutan^33^, Nepal^96,97^, Pakistan^16,98,99^, Singapore^34,100^, Malaysia^101^ and China^102,103^. Empirical evidence indicates the presence of Indian sub-continent lineage in the Middle East since early 1990s^55,104^. Though DENV-2 cosmopolitan genotype has historically been the predominant serotype in the African continent^23^, recent evidence suggests a notable presence of Indian sub-continent lineage, especially in East Africa and Indian Ocean Islands. This lineage has been associated with outbreaks in Yemen ^105^, Kenya^57^, Sudan^60^, Angola^15^ and Seychelles^17,106^ over the last decade. Dengue has been reported in Africa since the nineteenth century^107^ and is present in over 30 African countries. Sub-Saharan Africa carries an estimated 16% of the annual worldwide burden^108^. However, dengue is often under-reported and its epidemiology remains poorly characterized in Africa^109^. Rapid urbanization, economic expansion, poor environmental management and presence of vectors happening in many African countries are favorable for the sustained transmission of DENV in the continent^109^. Recent genotype evidence thus suggests an important role played by the Indian sub-continent lineage in DENV-2 epidemiology in Africa.

## Limitations

One of the limitations of present study is the difficulty in determining differences of clinical severity associated with different lineages of DENV-2 cosmopolitan genotype due to lack of data. Such information is immensely useful for outbreak risk assessment. Phenotypic differences have largely been described for individual isolates, and the population level analyses have often used the association of a lineage with outbreaks/epidemics as a proxy for fitness and virulence. Secondly, the sequencing biasness of a particular genotype/lineage could potentially affect the overall diversity estimates. This could happen either due to temporal or spatial limitations in sequencing efforts. DENVs are RNA viruses that mutate relatively fast and generate mutant spectra. Therefore, sampling has a profound effect on diversity estimates. Since DENV mutates during replication, transmission intensity tends to facilitate the diversity. Therefore, analyses on virus diversity are invariably affected by the sampling and sequence availability. Though the analysis included a large number of sequences from Singapore, they spanned over a 12-yr period from 2008 and belonged to all lineages described in this study at different proportions. All sequences were used to identify mutation signatures of different lineages with high confidence, but identical sequences were excluded from other genetic analyses to minimize the redundancy. Among over 3,000 cosmopolitan genotype *E* gene sequences obtained from different countries, nucleotide diversity showed a very weak negative and non-significant correlation with the number of sequences for each lineage (Pearson’s correlation = − 0.3; p = 0.5), suggesting that the diversity reported in the present study is unlikely to be an outcome of sequencing biasness. Moreover, the formation of lineages, as described in this study, is a long term evolutionary process that is less affected by the sampling.

## Conclusions

The current study provides deep insights into the molecular epidemiology of cosmopolitan genotype that forms an integral part of outbreak risk assessment in areas where DENV is endemic. The analyses demonstrated that cosmopolitan genotype has spread through an extensive spatial network, allowing distinct intra-genotype lineages to dominate in different geographies. High intra-genotype heterogeneity reflected intense evolutionary forces acting within the cosmopolitan genotype. Findings indicated a major split in the cosmopolitan genotype, and suggested that Indian sub-continent lineage is maturing into a separate genotype. This has important implications in the current understanding of DENV-2 genotype classifications. The notable genetic characteristics that appeared during the evolution and global expansion of cosmopolitan genotype warrant further investigations into the phenotype differences, host adaptation and survival of its lineages that affect DENV-2 epidemiology in endemic settings.

## Supplementary Information

Supplementary Information 1.

Supplementary Figure S1.

Supplementary Figure S2.

Supplementary Figure S3.

## Data Availability

Envelope gene sequences and complete genome sequences generated during the present study were deposited in GenBank database under the accession numbers MW510024–MW510156, MW510238–MW510994 and MW512341–MW512498.

## Abbreviations

BF: Bayes factor
BSSVS: Bayesian Stochastic Search Variable studies
DENV: Dengue virus
E: Envelope gene
ESS: Effective sample size
FEL: Fixed effects likelihood
FUBAR: Fast unbiased Bayesian approximation
GTR: General time reversible
HPD: Highest posterior density
M: Membrane
MCC: Maximum clade credibility
MCMC: Markov Chain Monte Carlo
MEME: Mixed effect model of evolution
NS: Non-structural proteins
preM: Pre-membrane
SLAC: Single likelihood ancestor counting
tMRCA: Time to most recent common ancestor
