# Supplementary material for: Evolution, heterogeneity and global dispersal of cosmopolitan genotype of Dengue virus type 2
Source: Sci Rep. 2021 Jun 29;11:13496. doi: 10.1038/s41598-021-92783-y (PMC8241877; doi:10.1038/s41598-021-92783-y)
Supplement: Supplementary file 1 — Supplementary Information 1. [file 41598_2021_92783_MOESM1_ESM.pdf]

Evolution, heterogeneity and global dispersal of cosmopolitan genotype of Dengue virus type 2

Surya Pavan Yenamandra[1], Carmen Koo[1], Suzanna Chiang[1], Lim Han Shi Jeri[2], Zhen Yuan Yeo[3], Lee Ching Ng[1,2], Hapuarachchige Chanditha Hapuarachchi[1]*

*Corresponding author

Supplementary Tables

Supplementary Table S1. Details of newly generated sequences from human cases and mosquitoes

NCBI accession number	Country	Year	Lineage of cosmopolitan genotype	Origin
MW510155	Singapore	2013	Indian sub-continent lineage	Mosquito
MW510957	Singapore	2012	Indian sub-continent lineage	Human
MW510958	Singapore	2013	Indian sub-continent lineage	Human
MW510959	Singapore	2013	Indian sub-continent lineage	Human
MW510960	Singapore	2013	Indian sub-continent lineage	Human
MW510961	Singapore	2013	Indian sub-continent lineage	Human
MW510962	Singapore	2013	Indian sub-continent lineage	Human
MW510963	Singapore	2013	Indian sub-continent lineage	Human
MW510964	Singapore	2013	Indian sub-continent lineage	Human
MW510965	Singapore	2013	Indian sub-continent lineage	Human
MW510966	Singapore	2013	Indian sub-continent lineage	Human

MW510967	Singapore	2013	Indian sub-continent lineage	Human
MW510968	Singapore	2013	Indian sub-continent lineage	Human
MW510969	Singapore	2013	Indian sub-continent lineage	Human
MW510970	Singapore	2013	Indian sub-continent lineage	Human
MW510971	Singapore	2013	Indian sub-continent lineage	Human
MW510972	Singapore	2014	Indian sub-continent lineage	Human
MW510973	Singapore	2014	Indian sub-continent lineage	Human
MW510974	Singapore	2014	Indian sub-continent lineage	Human
MW510975	Singapore	2014	Indian sub-continent lineage	Human
MW510976	Singapore	2014	Indian sub-continent lineage	Human
MW510977	Singapore	2014	Indian sub-continent lineage	Human
MW510978	Singapore	2014	Indian sub-continent lineage	Human
MW510979	Singapore	2014	Indian sub-continent lineage	Human
MW510980	Singapore	2014	Indian sub-continent lineage	Human
MW510981	Singapore	2014	Indian sub-continent lineage	Human
MW510982	Singapore	2014	Indian sub-continent lineage	Human
MW510983	Singapore	2014	Indian sub-continent lineage	Human
MW510984	Singapore	2014	Indian sub-continent lineage	Human
MW510985	Singapore	2014	Indian sub-continent lineage	Human
MW510986	Singapore	2015	Indian sub-continent lineage	Human
MW510987	Singapore	2015	Indian sub-continent lineage	Human
MW512362	Singapore	2011	Indian sub-continent lineage	Human
MW512370	Singapore	2012	Indian sub-continent lineage	Human

MW512371	Singapore	2012	Indian sub-continent lineage	Human
MW512372	Singapore	2012	Indian sub-continent lineage	Human
MW512391	Singapore	2013	Indian sub-continent lineage	Human
MW512410	Singapore	2014	Indian sub-continent lineage	Human
MW512411	Singapore	2014	Indian sub-continent lineage	Human
MW512412	Singapore	2014	Indian sub-continent lineage	Human
MW512413	Singapore	2014	Indian sub-continent lineage	Human
MW512449	Singapore	2016	Indian sub-continent lineage	Human
MW512468	Singapore	2017	Indian sub-continent lineage	Human
MW512469	Singapore	2017	Indian sub-continent lineage	Human
MW512470	Singapore	2017	Indian sub-continent lineage	Human
MW512490	Singapore	2019	Indian sub-continent lineage	Human
MW510156	Singapore	2014	Indian sub-continent lineage	Mosquito
MW510263	Singapore	2013	Lineage1	Human
MW510264	Singapore	2013	Lineage1	Human
MW510265	Singapore	2013	Lineage1	Human
MW510266	Singapore	2013	Lineage1	Human
MW510270	Singapore	2013	Lineage1	Human
MW512380	Singapore	2012	Lineage1	Human
MW512408	Singapore	2013	Lineage1	Human
MW512409	Singapore	2013	Lineage1	Human
MW512431	Singapore	2014	Lineage1	Human
MW512432	Singapore	2014	Lineage1	Human

MW512433	Singapore	2014	Lineage1	Human
MW512434	Singapore	2014	Lineage1	Human
MW512467	Singapore	2016	Lineage1	Human
MW512489	Singapore	2018	Lineage1	Human
MW510024	Singapore	2012	Lineage2	Mosquito
MW510125	Singapore	2012	Lineage2	Mosquito
MW510126	Singapore	2012	Lineage2	Mosquito
MW510127	Singapore	2012	Lineage2	Mosquito
MW510128	Singapore	2012	Lineage2	Mosquito
MW510129	Singapore	2012	Lineage2	Mosquito
MW510130	Singapore	2012	Lineage2	Mosquito
MW510131	Singapore	2012	Lineage2	Mosquito
MW510132	Singapore	2012	Lineage2	Mosquito
MW510133	Singapore	2012	Lineage2	Mosquito
MW510134	Singapore	2012	Lineage2	Mosquito
MW510135	Singapore	2012	Lineage2	Mosquito
MW510136	Singapore	2012	Lineage2	Mosquito
MW510137	Singapore	2012	Lineage2	Mosquito
MW510138	Singapore	2012	Lineage2	Mosquito
MW510139	Singapore	2012	Lineage2	Mosquito
MW510140	Singapore	2012	Lineage2	Mosquito
MW510141	Singapore	2012	Lineage2	Mosquito
MW510142	Singapore	2012	Lineage2	Mosquito

MW510143	Singapore	2012	Lineage2	Mosquito
MW510144	Singapore	2012	Lineage2	Mosquito
MW510145	Singapore	2012	Lineage2	Mosquito
MW510146	Singapore	2012	Lineage2	Mosquito
MW510147	Singapore	2012	Lineage2	Mosquito
MW510148	Singapore	2012	Lineage2	Mosquito
MW510149	Singapore	2012	Lineage2	Mosquito
MW510150	Singapore	2012	Lineage2	Mosquito
MW510151	Singapore	2013	Lineage2	Mosquito
MW510152	Singapore	2013	Lineage2	Mosquito
MW510238	Singapore	2008	Lineage2	Human
MW510239	Singapore	2008	Lineage2	Human
MW510240	Singapore	2008	Lineage2	Human
MW510241	Singapore	2008	Lineage2	Human
MW510242	Singapore	2010	Lineage2	Human
MW510243	Singapore	2010	Lineage2	Human
MW510245	Singapore	2011	Lineage2	Human
MW510252	Singapore	2011	Lineage2	Human
MW510256	Singapore	2012	Lineage2	Human
MW510257	Singapore	2012	Lineage2	Human
MW510267	Singapore	2013	Lineage2	Human
MW510721	Singapore	2013	Lineage2	Human
MW510722	Singapore	2013	Lineage2	Human

MW510723	Singapore	2013	Lineage2	Human
MW510724	Singapore	2013	Lineage2	Human
MW510725	Singapore	2013	Lineage2	Human
MW510726	Singapore	2013	Lineage2	Human
MW510727	Singapore	2013	Lineage2	Human
MW510728	Singapore	2013	Lineage2	Human
MW510729	Singapore	2013	Lineage2	Human
MW510730	Singapore	2013	Lineage2	Human
MW510731	Singapore	2013	Lineage2	Human
MW510732	Singapore	2013	Lineage2	Human
MW510733	Singapore	2013	Lineage2	Human
MW510734	Singapore	2013	Lineage2	Human
MW510735	Singapore	2013	Lineage2	Human
MW510736	Singapore	2013	Lineage2	Human
MW510737	Singapore	2013	Lineage2	Human
MW510738	Singapore	2013	Lineage2	Human
MW510739	Singapore	2013	Lineage2	Human
MW510740	Singapore	2013	Lineage2	Human
MW510741	Singapore	2013	Lineage2	Human
MW510742	Singapore	2011	Lineage2	Human
MW510743	Singapore	2011	Lineage2	Human
MW510744	Singapore	2011	Lineage2	Human
MW510745	Singapore	2011	Lineage2	Human

MW510746	Singapore	2011	Lineage2	Human
MW510747	Singapore	2011	Lineage2	Human
MW510748	Singapore	2011	Lineage2	Human
MW510749	Singapore	2011	Lineage2	Human
MW510750	Singapore	2011	Lineage2	Human
MW510751	Singapore	2011	Lineage2	Human
MW510752	Singapore	2011	Lineage2	Human
MW510753	Singapore	2011	Lineage2	Human
MW510754	Singapore	2011	Lineage2	Human
MW510755	Singapore	2011	Lineage2	Human
MW510756	Singapore	2011	Lineage2	Human
MW510757	Singapore	2011	Lineage2	Human
MW510758	Singapore	2011	Lineage2	Human
MW510759	Singapore	2011	Lineage2	Human
MW510760	Singapore	2011	Lineage2	Human
MW510761	Singapore	2011	Lineage2	Human
MW510762	Singapore	2011	Lineage2	Human
MW510763	Singapore	2011	Lineage2	Human
MW510764	Singapore	2011	Lineage2	Human
MW510765	Singapore	2011	Lineage2	Human
MW510766	Singapore	2011	Lineage2	Human
MW510767	Singapore	2011	Lineage2	Human
MW510768	Singapore	2011	Lineage2	Human

MW510769	Singapore	2011	Lineage2	Human
MW510770	Singapore	2011	Lineage2	Human
MW510771	Singapore	2011	Lineage2	Human
MW510772	Singapore	2011	Lineage2	Human
MW510773	Singapore	2011	Lineage2	Human
MW510774	Singapore	2011	Lineage2	Human
MW510775	Singapore	2011	Lineage2	Human
MW510776	Singapore	2011	Lineage2	Human
MW510777	Singapore	2011	Lineage2	Human
MW510778	Singapore	2011	Lineage2	Human
MW510779	Singapore	2011	Lineage2	Human
MW510780	Singapore	2012	Lineage2	Human
MW510781	Singapore	2012	Lineage2	Human
MW510782	Singapore	2012	Lineage2	Human
MW510783	Singapore	2012	Lineage2	Human
MW510784	Singapore	2012	Lineage2	Human
MW510785	Singapore	2012	Lineage2	Human
MW510786	Singapore	2012	Lineage2	Human
MW510787	Singapore	2012	Lineage2	Human
MW510788	Singapore	2012	Lineage2	Human
MW510789	Singapore	2012	Lineage2	Human
MW510790	Singapore	2012	Lineage2	Human
MW510791	Singapore	2012	Lineage2	Human

MW510792	Singapore	2012	Lineage2	Human
MW510793	Singapore	2012	Lineage2	Human
MW510794	Singapore	2012	Lineage2	Human
MW510795	Singapore	2012	Lineage2	Human
MW510796	Singapore	2012	Lineage2	Human
MW510797	Singapore	2012	Lineage2	Human
MW510798	Singapore	2012	Lineage2	Human
MW510799	Singapore	2012	Lineage2	Human
MW510800	Singapore	2012	Lineage2	Human
MW510801	Singapore	2012	Lineage2	Human
MW510802	Singapore	2012	Lineage2	Human
MW510803	Singapore	2012	Lineage2	Human
MW510804	Singapore	2012	Lineage2	Human
MW510805	Singapore	2012	Lineage2	Human
MW510806	Singapore	2012	Lineage2	Human
MW510807	Singapore	2012	Lineage2	Human
MW510808	Singapore	2012	Lineage2	Human
MW510809	Singapore	2012	Lineage2	Human
MW510810	Singapore	2012	Lineage2	Human
MW510811	Singapore	2012	Lineage2	Human
MW510812	Singapore	2012	Lineage2	Human
MW510813	Singapore	2012	Lineage2	Human
MW510814	Singapore	2012	Lineage2	Human

MW510815	Singapore	2012	Lineage2	Human
MW510816	Singapore	2012	Lineage2	Human
MW510817	Singapore	2012	Lineage2	Human
MW510818	Singapore	2012	Lineage2	Human
MW510819	Singapore	2012	Lineage2	Human
MW510820	Singapore	2012	Lineage2	Human
MW510821	Singapore	2012	Lineage2	Human
MW510822	Singapore	2012	Lineage2	Human
MW510823	Singapore	2012	Lineage2	Human
MW510824	Singapore	2013	Lineage2	Human
MW510825	Singapore	2013	Lineage2	Human
MW510826	Singapore	2013	Lineage2	Human
MW510827	Singapore	2013	Lineage2	Human
MW510828	Singapore	2013	Lineage2	Human
MW510829	Singapore	2013	Lineage2	Human
MW510830	Singapore	2013	Lineage2	Human
MW510831	Singapore	2013	Lineage2	Human
MW510832	Singapore	2013	Lineage2	Human
MW510833	Singapore	2013	Lineage2	Human
MW510834	Singapore	2013	Lineage2	Human
MW510835	Singapore	2013	Lineage2	Human
MW510836	Singapore	2013	Lineage2	Human
MW510837	Singapore	2015	Lineage2	Human

MW512342	Singapore	2007	Lineage2	Human
MW512343	Singapore	2007	Lineage2	Human
MW512344	Singapore	2007	Lineage2	Human
MW512345	Singapore	2007	Lineage2	Human
MW512346	Singapore	2007	Lineage2	Human
MW512347	Singapore	2007	Lineage2	Human
MW512348	Singapore	2007	Lineage2	Human
MW512349	Singapore	2007	Lineage2	Human
MW512350	Singapore	2008	Lineage2	Human
MW512351	Singapore	2008	Lineage2	Human
MW512352	Singapore	2008	Lineage2	Human
MW512353	Singapore	2008	Lineage2	Human
MW512354	Singapore	2009	Lineage2	Human
MW512355	Singapore	2009	Lineage2	Human
MW512356	Singapore	2009	Lineage2	Human
MW512357	Singapore	2010	Lineage2	Human
MW512358	Singapore	2010	Lineage2	Human
MW512359	Singapore	2010	Lineage2	Human
MW512360	Singapore	2010	Lineage2	Human
MW512361	Singapore	2010	Lineage2	Human
MW512363	Singapore	2011	Lineage2	Human
MW512373	Singapore	2012	Lineage2	Human
MW512374	Singapore	2012	Lineage2	Human

MW512381	Singapore	2013	Lineage2	Human
MW512382	Singapore	2013	Lineage2	Human
MW512383	Singapore	2013	Lineage2	Human
MW512414	Singapore	2014	Lineage2	Human
MW510052	Singapore	2018	Lineage3	Mosquito
MW510254	Singapore	2012	Lineage3	Human
MW510259	Singapore	2012	Lineage3	Human
MW510260	Singapore	2012	Lineage3	Human
MW510261	Singapore	2012	Lineage3	Human
MW510262	Singapore	2013	Lineage3	Human
MW510269	Singapore	2013	Lineage3	Human
MW510271	Singapore	2013	Lineage3	Human
MW510272	Singapore	2013	Lineage3	Human
MW510273	Singapore	2013	Lineage3	Human
MW510277	Singapore	2015	Lineage3	Human
MW510279	Singapore	2016	Lineage3	Human
MW510280	Singapore	2016	Lineage3	Human
MW510281	Singapore	2016	Lineage3	Human
MW512364	Singapore	2011	Lineage3	Human
MW512375	Singapore	2012	Lineage3	Human
MW512376	Singapore	2012	Lineage3	Human
MW512384	Singapore	2013	Lineage3	Human
MW512385	Singapore	2013	Lineage3	Human

MW512386	Singapore	2013	Lineage3	Human
MW512415	Singapore	2014	Lineage3	Human
MW512450	Singapore	2016	Lineage3	Human
MW512479	Singapore	2018	Lineage3	Human
MW512480	Singapore	2018	Lineage3	Human
MW510025	Singapore	2013	Lineage4	Mosquito
MW510026	Singapore	2013	Lineage4	Mosquito
MW510027	Singapore	2016	Lineage4	Mosquito
MW510153	Singapore	2014	Lineage4	Mosquito
MW510154	Singapore	2014	Lineage4	Mosquito
MW510255	Singapore	2012	Lineage4	Human
MW510258	Singapore	2012	Lineage4	Human
MW510274	Singapore	2013	Lineage4	Human
MW510275	Singapore	2013	Lineage4	Human
MW510838	Singapore	2013	Lineage4	Human
MW510839	Singapore	2013	Lineage4	Human
MW510840	Singapore	2013	Lineage4	Human
MW510841	Singapore	2013	Lineage4	Human
MW510842	Singapore	2013	Lineage4	Human
MW510843	Singapore	2013	Lineage4	Human
MW510844	Singapore	2013	Lineage4	Human
MW510845	Singapore	2013	Lineage4	Human
MW510846	Singapore	2013	Lineage4	Human

MW510847	Singapore	2013	Lineage4	Human
MW510848	Singapore	2013	Lineage4	Human
MW510849	Singapore	2013	Lineage4	Human
MW510850	Singapore	2013	Lineage4	Human
MW510851	Singapore	2013	Lineage4	Human
MW510852	Singapore	2013	Lineage4	Human
MW510853	Singapore	2013	Lineage4	Human
MW510854	Singapore	2013	Lineage4	Human
MW510855	Singapore	2013	Lineage4	Human
MW510856	Singapore	2013	Lineage4	Human
MW510857	Singapore	2013	Lineage4	Human
MW510858	Singapore	2013	Lineage4	Human
MW510859	Singapore	2013	Lineage4	Human
MW510860	Singapore	2013	Lineage4	Human
MW510861	Singapore	2012	Lineage4	Human
MW510862	Singapore	2012	Lineage4	Human
MW510863	Singapore	2012	Lineage4	Human
MW510864	Singapore	2012	Lineage4	Human
MW510865	Singapore	2012	Lineage4	Human
MW510866	Singapore	2012	Lineage4	Human
MW510867	Singapore	2012	Lineage4	Human
MW510868	Singapore	2012	Lineage4	Human
MW510869	Singapore	2012	Lineage4	Human

MW510870	Singapore	2012	Lineage4	Human
MW510871	Singapore	2012	Lineage4	Human
MW510872	Singapore	2012	Lineage4	Human
MW510873	Singapore	2012	Lineage4	Human
MW510874	Singapore	2012	Lineage4	Human
MW510875	Singapore	2012	Lineage4	Human
MW510876	Singapore	2012	Lineage4	Human
MW510877	Singapore	2012	Lineage4	Human
MW510878	Singapore	2012	Lineage4	Human
MW510879	Singapore	2012	Lineage4	Human
MW510880	Singapore	2012	Lineage4	Human
MW510881	Singapore	2012	Lineage4	Human
MW510882	Singapore	2012	Lineage4	Human
MW510883	Singapore	2012	Lineage4	Human
MW510884	Singapore	2012	Lineage4	Human
MW510885	Singapore	2013	Lineage4	Human
MW510886	Singapore	2013	Lineage4	Human
MW510887	Singapore	2013	Lineage4	Human
MW510888	Singapore	2013	Lineage4	Human
MW510889	Singapore	2013	Lineage4	Human
MW510890	Singapore	2013	Lineage4	Human
MW510891	Singapore	2013	Lineage4	Human
MW510892	Singapore	2013	Lineage4	Human

MW510893	Singapore	2013	Lineage4	Human
MW510894	Singapore	2013	Lineage4	Human
MW510895	Singapore	2013	Lineage4	Human
MW510896	Singapore	2013	Lineage4	Human
MW510897	Singapore	2013	Lineage4	Human
MW510898	Singapore	2013	Lineage4	Human
MW510899	Singapore	2013	Lineage4	Human
MW510900	Singapore	2013	Lineage4	Human
MW510901	Singapore	2013	Lineage4	Human
MW510902	Singapore	2013	Lineage4	Human
MW510903	Singapore	2013	Lineage4	Human
MW510904	Singapore	2013	Lineage4	Human
MW510905	Singapore	2013	Lineage4	Human
MW510906	Singapore	2013	Lineage4	Human
MW510907	Singapore	2013	Lineage4	Human
MW510908	Singapore	2013	Lineage4	Human
MW510909	Singapore	2013	Lineage4	Human
MW510910	Singapore	2013	Lineage4	Human
MW510911	Singapore	2013	Lineage4	Human
MW510912	Singapore	2013	Lineage4	Human
MW510913	Singapore	2013	Lineage4	Human
MW510914	Singapore	2013	Lineage4	Human
MW510915	Singapore	2013	Lineage4	Human

MW510916	Singapore	2013	Lineage4	Human
MW510917	Singapore	2013	Lineage4	Human
MW510918	Singapore	2013	Lineage4	Human
MW510919	Singapore	2013	Lineage4	Human
MW510920	Singapore	2013	Lineage4	Human
MW510921	Singapore	2013	Lineage4	Human
MW510922	Singapore	2013	Lineage4	Human
MW510923	Singapore	2013	Lineage4	Human
MW510924	Singapore	2013	Lineage4	Human
MW510925	Singapore	2013	Lineage4	Human
MW510926	Singapore	2013	Lineage4	Human
MW510927	Singapore	2013	Lineage4	Human
MW510928	Singapore	2013	Lineage4	Human
MW510929	Singapore	2013	Lineage4	Human
MW510930	Singapore	2013	Lineage4	Human
MW510931	Singapore	2013	Lineage4	Human
MW510932	Singapore	2013	Lineage4	Human
MW510933	Singapore	2013	Lineage4	Human
MW510934	Singapore	2013	Lineage4	Human
MW510935	Singapore	2013	Lineage4	Human
MW510936	Singapore	2013	Lineage4	Human
MW510937	Singapore	2013	Lineage4	Human
MW510938	Singapore	2013	Lineage4	Human

MW510939	Singapore	2013	Lineage4	Human
MW510940	Singapore	2013	Lineage4	Human
MW510941	Singapore	2013	Lineage4	Human
MW510942	Singapore	2013	Lineage4	Human
MW510943	Singapore	2013	Lineage4	Human
MW510944	Singapore	2013	Lineage4	Human
MW510945	Singapore	2013	Lineage4	Human
MW510946	Singapore	2013	Lineage4	Human
MW510947	Singapore	2013	Lineage4	Human
MW510948	Singapore	2013	Lineage4	Human
MW510949	Singapore	2013	Lineage4	Human
MW510950	Singapore	2013	Lineage4	Human
MW510951	Singapore	2013	Lineage4	Human
MW510952	Singapore	2014	Lineage4	Human
MW510953	Singapore	2014	Lineage4	Human
MW510954	Singapore	2014	Lineage4	Human
MW510955	Singapore	2014	Lineage4	Human
MW510956	Singapore	2014	Lineage4	Human
MW512369	Singapore	2011	Lineage4	Human
MW512377	Singapore	2012	Lineage4	Human
MW512378	Singapore	2012	Lineage4	Human
MW512379	Singapore	2012	Lineage4	Human
MW512404	Singapore	2013	Lineage4	Human

MW512405	Singapore	2013	Lineage4	Human
MW512406	Singapore	2013	Lineage4	Human
MW512407	Singapore	2013	Lineage4	Human
MW512428	Singapore	2014	Lineage4	Human
MW512429	Singapore	2014	Lineage4	Human
MW512430	Singapore	2014	Lineage4	Human
MW512448	Singapore	2015	Lineage4	Human
MW512464	Singapore	2016	Lineage4	Human
MW512465	Singapore	2016	Lineage4	Human
MW512466	Singapore	2016	Lineage4	Human
MW510028	Singapore	2018	Lineage5	Mosquito
MW510029	Singapore	2018	Lineage5	Mosquito
MW510030	Singapore	2018	Lineage5	Mosquito
MW510031	Singapore	2018	Lineage5	Mosquito
MW510032	Singapore	2018	Lineage5	Mosquito
MW510033	Singapore	2018	Lineage5	Mosquito
MW510034	Singapore	2018	Lineage5	Mosquito
MW510035	Singapore	2018	Lineage5	Mosquito
MW510036	Singapore	2018	Lineage5	Mosquito
MW510039	Singapore	2018	Lineage5	Mosquito
MW510040	Singapore	2018	Lineage5	Mosquito
MW510041	Singapore	2018	Lineage5	Mosquito
MW510042	Singapore	2018	Lineage5	Mosquito

MW510043	Singapore	2018	Lineage5	Mosquito
MW510044	Singapore	2018	Lineage5	Mosquito
MW510045	Singapore	2018	Lineage5	Mosquito
MW510046	Singapore	2018	Lineage5	Mosquito
MW510047	Singapore	2018	Lineage5	Mosquito
MW510048	Singapore	2018	Lineage5	Mosquito
MW510049	Singapore	2018	Lineage5	Mosquito
MW510050	Singapore	2018	Lineage5	Mosquito
MW510051	Singapore	2018	Lineage5	Mosquito
MW510053	Singapore	2018	Lineage5	Mosquito
MW510054	Singapore	2019	Lineage5	Mosquito
MW510055	Singapore	2019	Lineage5	Mosquito
MW510056	Singapore	2019	Lineage5	Mosquito
MW510057	Singapore	2019	Lineage5	Mosquito
MW510058	Singapore	2019	Lineage5	Mosquito
MW510059	Singapore	2019	Lineage5	Mosquito
MW510060	Singapore	2019	Lineage5	Mosquito
MW510061	Singapore	2019	Lineage5	Mosquito
MW510062	Singapore	2019	Lineage5	Mosquito
MW510063	Singapore	2019	Lineage5	Mosquito
MW510064	Singapore	2019	Lineage5	Mosquito
MW510065	Singapore	2019	Lineage5	Mosquito
MW510066	Singapore	2019	Lineage5	Mosquito

MW510067	Singapore	2019	Lineage5	Mosquito
MW510068	Singapore	2019	Lineage5	Mosquito
MW510069	Singapore	2019	Lineage5	Mosquito
MW510070	Singapore	2019	Lineage5	Mosquito
MW510071	Singapore	2019	Lineage5	Mosquito
MW510072	Singapore	2019	Lineage5	Mosquito
MW510073	Singapore	2019	Lineage5	Mosquito
MW510074	Singapore	2019	Lineage5	Mosquito
MW510075	Singapore	2019	Lineage5	Mosquito
MW510076	Singapore	2019	Lineage5	Mosquito
MW510077	Singapore	2019	Lineage5	Mosquito
MW510078	Singapore	2019	Lineage5	Mosquito
MW510079	Singapore	2019	Lineage5	Mosquito
MW510080	Singapore	2019	Lineage5	Mosquito
MW510081	Singapore	2014	Lineage5	Mosquito
MW510082	Singapore	2015	Lineage5	Mosquito
MW510083	Singapore	2015	Lineage5	Mosquito
MW510084	Singapore	2015	Lineage5	Mosquito
MW510085	Singapore	2015	Lineage5	Mosquito
MW510086	Singapore	2015	Lineage5	Mosquito
MW510087	Singapore	2015	Lineage5	Mosquito
MW510088	Singapore	2015	Lineage5	Mosquito
MW510089	Singapore	2015	Lineage5	Mosquito

MW510090	Singapore	2015	Lineage5	Mosquito
MW510091	Singapore	2015	Lineage5	Mosquito
MW510092	Singapore	2015	Lineage5	Mosquito
MW510093	Singapore	2015	Lineage5	Mosquito
MW510094	Singapore	2015	Lineage5	Mosquito
MW510095	Singapore	2015	Lineage5	Mosquito
MW510096	Singapore	2015	Lineage5	Mosquito
MW510097	Singapore	2016	Lineage5	Mosquito
MW510098	Singapore	2016	Lineage5	Mosquito
MW510099	Singapore	2016	Lineage5	Mosquito
MW510100	Singapore	2016	Lineage5	Mosquito
MW510101	Singapore	2016	Lineage5	Mosquito
MW510102	Singapore	2016	Lineage5	Mosquito
MW510103	Singapore	2016	Lineage5	Mosquito
MW510104	Singapore	2016	Lineage5	Mosquito
MW510105	Singapore	2016	Lineage5	Mosquito
MW510106	Singapore	2016	Lineage5	Mosquito
MW510107	Singapore	2016	Lineage5	Mosquito
MW510108	Singapore	2016	Lineage5	Mosquito
MW510109	Singapore	2016	Lineage5	Mosquito
MW510110	Singapore	2016	Lineage5	Mosquito
MW510111	Singapore	2016	Lineage5	Mosquito
MW510112	Singapore	2016	Lineage5	Mosquito

MW510113	Singapore	2016	Lineage5	Mosquito
MW510114	Singapore	2016	Lineage5	Mosquito
MW510115	Singapore	2016	Lineage5	Mosquito
MW510116	Singapore	2016	Lineage5	Mosquito
MW510119	Singapore	2017	Lineage5	Mosquito
MW510121	Singapore	2016	Lineage5	Mosquito
MW510122	Singapore	2016	Lineage5	Mosquito
MW510123	Singapore	2016	Lineage5	Mosquito
MW510124	Singapore	2016	Lineage5	Mosquito
MW510244	Singapore	2011	Lineage5	Human
MW510246	Singapore	2011	Lineage5	Human
MW510247	Singapore	2011	Lineage5	Human
MW510248	Singapore	2011	Lineage5	Human
MW510249	Singapore	2011	Lineage5	Human
MW510250	Singapore	2011	Lineage5	Human
MW510251	Singapore	2011	Lineage5	Human
MW510253	Singapore	2011	Lineage5	Human
MW510268	Singapore	2013	Lineage5	Human
MW510276	Singapore	2014	Lineage5	Human
MW510278	Singapore	2015	Lineage5	Human
MW510282	Singapore	2018	Lineage5	Human
MW510283	Singapore	2018	Lineage5	Human
MW510284	Singapore	2018	Lineage5	Human

MW510285	Singapore	2018	Lineage5	Human
MW510286	Singapore	2018	Lineage5	Human
MW510287	Singapore	2018	Lineage5	Human
MW510288	Singapore	2018	Lineage5	Human
MW510289	Singapore	2018	Lineage5	Human
MW510290	Singapore	2018	Lineage5	Human
MW510291	Singapore	2018	Lineage5	Human
MW510292	Singapore	2018	Lineage5	Human
MW510293	Singapore	2018	Lineage5	Human
MW510294	Singapore	2018	Lineage5	Human
MW510295	Singapore	2018	Lineage5	Human
MW510296	Singapore	2018	Lineage5	Human
MW510297	Singapore	2018	Lineage5	Human
MW510298	Singapore	2018	Lineage5	Human
MW510299	Singapore	2018	Lineage5	Human
MW510300	Singapore	2018	Lineage5	Human
MW510301	Singapore	2018	Lineage5	Human
MW510302	Singapore	2018	Lineage5	Human
MW510303	Singapore	2018	Lineage5	Human
MW510304	Singapore	2018	Lineage5	Human
MW510305	Singapore	2018	Lineage5	Human
MW510306	Singapore	2019	Lineage5	Human
MW510307	Singapore	2019	Lineage5	Human

MW510308	Singapore	2019	Lineage5	Human
MW510309	Singapore	2019	Lineage5	Human
MW510310	Singapore	2019	Lineage5	Human
MW510311	Singapore	2019	Lineage5	Human
MW510312	Singapore	2019	Lineage5	Human
MW510313	Singapore	2019	Lineage5	Human
MW510314	Singapore	2019	Lineage5	Human
MW510315	Singapore	2019	Lineage5	Human
MW510316	Singapore	2019	Lineage5	Human
MW510317	Singapore	2019	Lineage5	Human
MW510318	Singapore	2019	Lineage5	Human
MW510319	Singapore	2019	Lineage5	Human
MW510320	Singapore	2019	Lineage5	Human
MW510321	Singapore	2019	Lineage5	Human
MW510322	Singapore	2019	Lineage5	Human
MW510323	Singapore	2019	Lineage5	Human
MW510324	Singapore	2019	Lineage5	Human
MW510325	Singapore	2019	Lineage5	Human
MW510326	Singapore	2019	Lineage5	Human
MW510327	Singapore	2019	Lineage5	Human
MW510328	Singapore	2019	Lineage5	Human
MW510329	Singapore	2019	Lineage5	Human
MW510330	Singapore	2019	Lineage5	Human

MW510331	Singapore	2019	Lineage5	Human
MW510332	Singapore	2019	Lineage5	Human
MW510333	Singapore	2019	Lineage5	Human
MW510334	Singapore	2019	Lineage5	Human
MW510335	Singapore	2019	Lineage5	Human
MW510336	Singapore	2019	Lineage5	Human
MW510337	Singapore	2019	Lineage5	Human
MW510338	Singapore	2019	Lineage5	Human
MW510339	Singapore	2019	Lineage5	Human
MW510340	Singapore	2019	Lineage5	Human
MW510341	Singapore	2019	Lineage5	Human
MW510342	Singapore	2019	Lineage5	Human
MW510343	Singapore	2019	Lineage5	Human
MW510344	Singapore	2013	Lineage5	Human
MW510345	Singapore	2013	Lineage5	Human
MW510346	Singapore	2013	Lineage5	Human
MW510347	Singapore	2013	Lineage5	Human
MW510348	Singapore	2013	Lineage5	Human
MW510349	Singapore	2013	Lineage5	Human
MW510350	Singapore	2013	Lineage5	Human
MW510351	Singapore	2013	Lineage5	Human
MW510352	Singapore	2014	Lineage5	Human
MW510353	Singapore	2014	Lineage5	Human

MW510354	Singapore	2014	Lineage5	Human
MW510355	Singapore	2014	Lineage5	Human
MW510356	Singapore	2014	Lineage5	Human
MW510357	Singapore	2014	Lineage5	Human
MW510358	Singapore	2014	Lineage5	Human
MW510359	Singapore	2014	Lineage5	Human
MW510360	Singapore	2014	Lineage5	Human
MW510361	Singapore	2014	Lineage5	Human
MW510362	Singapore	2014	Lineage5	Human
MW510363	Singapore	2014	Lineage5	Human
MW510364	Singapore	2014	Lineage5	Human
MW510365	Singapore	2014	Lineage5	Human
MW510366	Singapore	2014	Lineage5	Human
MW510367	Singapore	2014	Lineage5	Human
MW510368	Singapore	2014	Lineage5	Human
MW510369	Singapore	2014	Lineage5	Human
MW510370	Singapore	2014	Lineage5	Human
MW510371	Singapore	2014	Lineage5	Human
MW510372	Singapore	2014	Lineage5	Human
MW510373	Singapore	2014	Lineage5	Human
MW510374	Singapore	2014	Lineage5	Human
MW510375	Singapore	2014	Lineage5	Human
MW510376	Singapore	2014	Lineage5	Human

MW510377	Singapore	2014	Lineage5	Human
MW510378	Singapore	2014	Lineage5	Human
MW510379	Singapore	2014	Lineage5	Human
MW510380	Singapore	2014	Lineage5	Human
MW510381	Singapore	2014	Lineage5	Human
MW510382	Singapore	2014	Lineage5	Human
MW510383	Singapore	2014	Lineage5	Human
MW510384	Singapore	2014	Lineage5	Human
MW510385	Singapore	2014	Lineage5	Human
MW510386	Singapore	2014	Lineage5	Human
MW510387	Singapore	2014	Lineage5	Human
MW510388	Singapore	2014	Lineage5	Human
MW510389	Singapore	2014	Lineage5	Human
MW510390	Singapore	2014	Lineage5	Human
MW510391	Singapore	2014	Lineage5	Human
MW510392	Singapore	2014	Lineage5	Human
MW510393	Singapore	2014	Lineage5	Human
MW510394	Singapore	2014	Lineage5	Human
MW510395	Singapore	2014	Lineage5	Human
MW510396	Singapore	2014	Lineage5	Human
MW510397	Singapore	2014	Lineage5	Human
MW510398	Singapore	2014	Lineage5	Human
MW510399	Singapore	2014	Lineage5	Human

MW510400	Singapore	2014	Lineage5	Human
MW510401	Singapore	2014	Lineage5	Human
MW510402	Singapore	2014	Lineage5	Human
MW510403	Singapore	2014	Lineage5	Human
MW510404	Singapore	2014	Lineage5	Human
MW510405	Singapore	2014	Lineage5	Human
MW510406	Singapore	2014	Lineage5	Human
MW510407	Singapore	2014	Lineage5	Human
MW510408	Singapore	2014	Lineage5	Human
MW510409	Singapore	2014	Lineage5	Human
MW510410	Singapore	2014	Lineage5	Human
MW510411	Singapore	2014	Lineage5	Human
MW510412	Singapore	2014	Lineage5	Human
MW510413	Singapore	2014	Lineage5	Human
MW510414	Singapore	2014	Lineage5	Human
MW510415	Singapore	2014	Lineage5	Human
MW510416	Singapore	2014	Lineage5	Human
MW510417	Singapore	2014	Lineage5	Human
MW510418	Singapore	2014	Lineage5	Human
MW510419	Singapore	2014	Lineage5	Human
MW510420	Singapore	2014	Lineage5	Human
MW510421	Singapore	2014	Lineage5	Human
MW510422	Singapore	2014	Lineage5	Human

MW510423	Singapore	2014	Lineage5	Human
MW510424	Singapore	2014	Lineage5	Human
MW510425	Singapore	2014	Lineage5	Human
MW510426	Singapore	2014	Lineage5	Human
MW510427	Singapore	2014	Lineage5	Human
MW510428	Singapore	2014	Lineage5	Human
MW510429	Singapore	2014	Lineage5	Human
MW510430	Singapore	2014	Lineage5	Human
MW510431	Singapore	2014	Lineage5	Human
MW510432	Singapore	2014	Lineage5	Human
MW510433	Singapore	2014	Lineage5	Human
MW510434	Singapore	2014	Lineage5	Human
MW510435	Singapore	2014	Lineage5	Human
MW510436	Singapore	2014	Lineage5	Human
MW510437	Singapore	2014	Lineage5	Human
MW510438	Singapore	2014	Lineage5	Human
MW510439	Singapore	2015	Lineage5	Human
MW510440	Singapore	2015	Lineage5	Human
MW510441	Singapore	2015	Lineage5	Human
MW510442	Singapore	2015	Lineage5	Human
MW510443	Singapore	2015	Lineage5	Human
MW510444	Singapore	2015	Lineage5	Human
MW510445	Singapore	2015	Lineage5	Human

MW510446	Singapore	2015	Lineage5	Human
MW510447	Singapore	2015	Lineage5	Human
MW510448	Singapore	2015	Lineage5	Human
MW510449	Singapore	2015	Lineage5	Human
MW510450	Singapore	2015	Lineage5	Human
MW510451	Singapore	2015	Lineage5	Human
MW510452	Singapore	2015	Lineage5	Human
MW510453	Singapore	2015	Lineage5	Human
MW510454	Singapore	2015	Lineage5	Human
MW510455	Singapore	2015	Lineage5	Human
MW510456	Singapore	2015	Lineage5	Human
MW510457	Singapore	2015	Lineage5	Human
MW510458	Singapore	2015	Lineage5	Human
MW510459	Singapore	2015	Lineage5	Human
MW510460	Singapore	2015	Lineage5	Human
MW510461	Singapore	2015	Lineage5	Human
MW510462	Singapore	2015	Lineage5	Human
MW510463	Singapore	2015	Lineage5	Human
MW510464	Singapore	2015	Lineage5	Human
MW510465	Singapore	2015	Lineage5	Human
MW510466	Singapore	2015	Lineage5	Human
MW510467	Singapore	2015	Lineage5	Human
MW510468	Singapore	2015	Lineage5	Human

MW510469	Singapore	2015	Lineage5	Human
MW510470	Singapore	2015	Lineage5	Human
MW510471	Singapore	2015	Lineage5	Human
MW510472	Singapore	2015	Lineage5	Human
MW510473	Singapore	2015	Lineage5	Human
MW510474	Singapore	2015	Lineage5	Human
MW510475	Singapore	2015	Lineage5	Human
MW510476	Singapore	2015	Lineage5	Human
MW510477	Singapore	2015	Lineage5	Human
MW510478	Singapore	2015	Lineage5	Human
MW510479	Singapore	2015	Lineage5	Human
MW510480	Singapore	2015	Lineage5	Human
MW510481	Singapore	2015	Lineage5	Human
MW510482	Singapore	2015	Lineage5	Human
MW510483	Singapore	2015	Lineage5	Human
MW510484	Singapore	2015	Lineage5	Human
MW510485	Singapore	2015	Lineage5	Human
MW510486	Singapore	2015	Lineage5	Human
MW510487	Singapore	2015	Lineage5	Human
MW510488	Singapore	2015	Lineage5	Human
MW510489	Singapore	2015	Lineage5	Human
MW510490	Singapore	2015	Lineage5	Human
MW510491	Singapore	2015	Lineage5	Human

MW510492	Singapore	2015	Lineage5	Human
MW510493	Singapore	2015	Lineage5	Human
MW510494	Singapore	2015	Lineage5	Human
MW510495	Singapore	2015	Lineage5	Human
MW510496	Singapore	2015	Lineage5	Human
MW510497	Singapore	2015	Lineage5	Human
MW510498	Singapore	2015	Lineage5	Human
MW510499	Singapore	2015	Lineage5	Human
MW510500	Singapore	2015	Lineage5	Human
MW510501	Singapore	2015	Lineage5	Human
MW510502	Singapore	2015	Lineage5	Human
MW510503	Singapore	2015	Lineage5	Human
MW510504	Singapore	2015	Lineage5	Human
MW510505	Singapore	2015	Lineage5	Human
MW510506	Singapore	2015	Lineage5	Human
MW510507	Singapore	2015	Lineage5	Human
MW510508	Singapore	2015	Lineage5	Human
MW510509	Singapore	2015	Lineage5	Human
MW510510	Singapore	2015	Lineage5	Human
MW510511	Singapore	2015	Lineage5	Human
MW510512	Singapore	2015	Lineage5	Human
MW510513	Singapore	2015	Lineage5	Human
MW510514	Singapore	2015	Lineage5	Human

MW510515	Singapore	2015	Lineage5	Human
MW510516	Singapore	2015	Lineage5	Human
MW510517	Singapore	2015	Lineage5	Human
MW510518	Singapore	2015	Lineage5	Human
MW510519	Singapore	2015	Lineage5	Human
MW510520	Singapore	2015	Lineage5	Human
MW510521	Singapore	2015	Lineage5	Human
MW510522	Singapore	2015	Lineage5	Human
MW510523	Singapore	2015	Lineage5	Human
MW510524	Singapore	2015	Lineage5	Human
MW510525	Singapore	2015	Lineage5	Human
MW510526	Singapore	2015	Lineage5	Human
MW510527	Singapore	2015	Lineage5	Human
MW510528	Singapore	2015	Lineage5	Human
MW510529	Singapore	2015	Lineage5	Human
MW510530	Singapore	2015	Lineage5	Human
MW510531	Singapore	2015	Lineage5	Human
MW510532	Singapore	2015	Lineage5	Human
MW510533	Singapore	2015	Lineage5	Human
MW510534	Singapore	2015	Lineage5	Human
MW510535	Singapore	2015	Lineage5	Human
MW510536	Singapore	2015	Lineage5	Human
MW510537	Singapore	2015	Lineage5	Human

MW510538	Singapore	2016	Lineage5	Human
MW510539	Singapore	2016	Lineage5	Human
MW510540	Singapore	2016	Lineage5	Human
MW510541	Singapore	2016	Lineage5	Human
MW510542	Singapore	2016	Lineage5	Human
MW510543	Singapore	2016	Lineage5	Human
MW510544	Singapore	2016	Lineage5	Human
MW510545	Singapore	2016	Lineage5	Human
MW510546	Singapore	2016	Lineage5	Human
MW510547	Singapore	2016	Lineage5	Human
MW510548	Singapore	2016	Lineage5	Human
MW510549	Singapore	2016	Lineage5	Human
MW510550	Singapore	2016	Lineage5	Human
MW510551	Singapore	2016	Lineage5	Human
MW510552	Singapore	2016	Lineage5	Human
MW510553	Singapore	2016	Lineage5	Human
MW510554	Singapore	2016	Lineage5	Human
MW510555	Singapore	2016	Lineage5	Human
MW510556	Singapore	2016	Lineage5	Human
MW510557	Singapore	2016	Lineage5	Human
MW510558	Singapore	2016	Lineage5	Human
MW510559	Singapore	2016	Lineage5	Human
MW510560	Singapore	2016	Lineage5	Human

MW510561	Singapore	2016	Lineage5	Human
MW510562	Singapore	2016	Lineage5	Human
MW510563	Singapore	2016	Lineage5	Human
MW510564	Singapore	2016	Lineage5	Human
MW510565	Singapore	2016	Lineage5	Human
MW510566	Singapore	2016	Lineage5	Human
MW510567	Singapore	2016	Lineage5	Human
MW510568	Singapore	2016	Lineage5	Human
MW510569	Singapore	2016	Lineage5	Human
MW510570	Singapore	2016	Lineage5	Human
MW510571	Singapore	2016	Lineage5	Human
MW510572	Singapore	2016	Lineage5	Human
MW510573	Singapore	2016	Lineage5	Human
MW510574	Singapore	2016	Lineage5	Human
MW510575	Singapore	2016	Lineage5	Human
MW510576	Singapore	2016	Lineage5	Human
MW510577	Singapore	2016	Lineage5	Human
MW510578	Singapore	2016	Lineage5	Human
MW510579	Singapore	2016	Lineage5	Human
MW510580	Singapore	2016	Lineage5	Human
MW510581	Singapore	2016	Lineage5	Human
MW510582	Singapore	2016	Lineage5	Human
MW510583	Singapore	2016	Lineage5	Human

MW510584	Singapore	2016	Lineage5	Human
MW510585	Singapore	2016	Lineage5	Human
MW510586	Singapore	2016	Lineage5	Human
MW510587	Singapore	2016	Lineage5	Human
MW510588	Singapore	2016	Lineage5	Human
MW510589	Singapore	2016	Lineage5	Human
MW510590	Singapore	2016	Lineage5	Human
MW510591	Singapore	2016	Lineage5	Human
MW510592	Singapore	2016	Lineage5	Human
MW510593	Singapore	2016	Lineage5	Human
MW510594	Singapore	2016	Lineage5	Human
MW510595	Singapore	2016	Lineage5	Human
MW510596	Singapore	2016	Lineage5	Human
MW510597	Singapore	2016	Lineage5	Human
MW510598	Singapore	2016	Lineage5	Human
MW510599	Singapore	2016	Lineage5	Human
MW510600	Singapore	2016	Lineage5	Human
MW510601	Singapore	2016	Lineage5	Human
MW510602	Singapore	2016	Lineage5	Human
MW510603	Singapore	2016	Lineage5	Human
MW510604	Singapore	2016	Lineage5	Human
MW510605	Singapore	2016	Lineage5	Human
MW510606	Singapore	2016	Lineage5	Human

MW510607	Singapore	2016	Lineage5	Human
MW510608	Singapore	2016	Lineage5	Human
MW510609	Singapore	2016	Lineage5	Human
MW510610	Singapore	2016	Lineage5	Human
MW510611	Singapore	2016	Lineage5	Human
MW510612	Singapore	2016	Lineage5	Human
MW510613	Singapore	2016	Lineage5	Human
MW510614	Singapore	2016	Lineage5	Human
MW510615	Singapore	2016	Lineage5	Human
MW510616	Singapore	2016	Lineage5	Human
MW510617	Singapore	2016	Lineage5	Human
MW510618	Singapore	2016	Lineage5	Human
MW510619	Singapore	2016	Lineage5	Human
MW510620	Singapore	2016	Lineage5	Human
MW510621	Singapore	2016	Lineage5	Human
MW510622	Singapore	2016	Lineage5	Human
MW510623	Singapore	2016	Lineage5	Human
MW510624	Singapore	2016	Lineage5	Human
MW510625	Singapore	2016	Lineage5	Human
MW510626	Singapore	2016	Lineage5	Human
MW510627	Singapore	2016	Lineage5	Human
MW510628	Singapore	2016	Lineage5	Human
MW510629	Singapore	2016	Lineage5	Human

MW510630	Singapore	2016	Lineage5	Human
MW510631	Singapore	2016	Lineage5	Human
MW510632	Singapore	2016	Lineage5	Human
MW510633	Singapore	2016	Lineage5	Human
MW510634	Singapore	2016	Lineage5	Human
MW510635	Singapore	2016	Lineage5	Human
MW510636	Singapore	2016	Lineage5	Human
MW510637	Singapore	2016	Lineage5	Human
MW510638	Singapore	2016	Lineage5	Human
MW510639	Singapore	2016	Lineage5	Human
MW510640	Singapore	2016	Lineage5	Human
MW510641	Singapore	2016	Lineage5	Human
MW510642	Singapore	2016	Lineage5	Human
MW510643	Singapore	2016	Lineage5	Human
MW510644	Singapore	2016	Lineage5	Human
MW510645	Singapore	2016	Lineage5	Human
MW510646	Singapore	2016	Lineage5	Human
MW510647	Singapore	2016	Lineage5	Human
MW510648	Singapore	2016	Lineage5	Human
MW510649	Singapore	2016	Lineage5	Human
MW510650	Singapore	2016	Lineage5	Human
MW510651	Singapore	2016	Lineage5	Human
MW510652	Singapore	2016	Lineage5	Human

MW510653	Singapore	2016	Lineage5	Human
MW510654	Singapore	2016	Lineage5	Human
MW510655	Singapore	2016	Lineage5	Human
MW510656	Singapore	2016	Lineage5	Human
MW510657	Singapore	2016	Lineage5	Human
MW510658	Singapore	2016	Lineage5	Human
MW510659	Singapore	2016	Lineage5	Human
MW510660	Singapore	2016	Lineage5	Human
MW510661	Singapore	2016	Lineage5	Human
MW510662	Singapore	2016	Lineage5	Human
MW510663	Singapore	2016	Lineage5	Human
MW510664	Singapore	2016	Lineage5	Human
MW510665	Singapore	2016	Lineage5	Human
MW510666	Singapore	2016	Lineage5	Human
MW510667	Singapore	2016	Lineage5	Human
MW510668	Singapore	2016	Lineage5	Human
MW510669	Singapore	2016	Lineage5	Human
MW510670	Singapore	2016	Lineage5	Human
MW510671	Singapore	2016	Lineage5	Human
MW510672	Singapore	2016	Lineage5	Human
MW510673	Singapore	2016	Lineage5	Human
MW510674	Singapore	2016	Lineage5	Human
MW510675	Singapore	2016	Lineage5	Human

MW510676	Singapore	2016	Lineage5	Human
MW510677	Singapore	2016	Lineage5	Human
MW510678	Singapore	2016	Lineage5	Human
MW510679	Singapore	2016	Lineage5	Human
MW510680	Singapore	2016	Lineage5	Human
MW510681	Singapore	2016	Lineage5	Human
MW510682	Singapore	2016	Lineage5	Human
MW510683	Singapore	2016	Lineage5	Human
MW510684	Singapore	2016	Lineage5	Human
MW510685	Singapore	2016	Lineage5	Human
MW510686	Singapore	2016	Lineage5	Human
MW510687	Singapore	2016	Lineage5	Human
MW510688	Singapore	2016	Lineage5	Human
MW510689	Singapore	2016	Lineage5	Human
MW510690	Singapore	2016	Lineage5	Human
MW510691	Singapore	2016	Lineage5	Human
MW510692	Singapore	2016	Lineage5	Human
MW510693	Singapore	2016	Lineage5	Human
MW510694	Singapore	2016	Lineage5	Human
MW510695	Singapore	2016	Lineage5	Human
MW510696	Singapore	2016	Lineage5	Human
MW510697	Singapore	2016	Lineage5	Human
MW510698	Singapore	2016	Lineage5	Human

MW510699	Singapore	2016	Lineage5	Human
MW510700	Singapore	2016	Lineage5	Human
MW510701	Singapore	2016	Lineage5	Human
MW510702	Singapore	2016	Lineage5	Human
MW510703	Singapore	2016	Lineage5	Human
MW510704	Singapore	2016	Lineage5	Human
MW510705	Singapore	2016	Lineage5	Human
MW510706	Singapore	2016	Lineage5	Human
MW510707	Singapore	2016	Lineage5	Human
MW510708	Singapore	2016	Lineage5	Human
MW510709	Singapore	2016	Lineage5	Human
MW510710	Singapore	2016	Lineage5	Human
MW510711	Singapore	2016	Lineage5	Human
MW510712	Singapore	2016	Lineage5	Human
MW510713	Singapore	2016	Lineage5	Human
MW510714	Singapore	2017	Lineage5	Human
MW510715	Singapore	2017	Lineage5	Human
MW510716	Singapore	2017	Lineage5	Human
MW510717	Singapore	2017	Lineage5	Human
MW510718	Singapore	2017	Lineage5	Human
MW510719	Singapore	2017	Lineage5	Human
MW510720	Singapore	2017	Lineage5	Human
MW512341	Singapore	2004	Lineage5	Human

MW512365	Singapore	2011	Lineage5	Human
MW512366	Singapore	2011	Lineage5	Human
MW512367	Singapore	2011	Lineage5	Human
MW512368	Singapore	2011	Lineage5	Human
MW512387	Singapore	2013	Lineage5	Human
MW512388	Singapore	2013	Lineage5	Human
MW512389	Singapore	2013	Lineage5	Human
MW512390	Singapore	2013	Lineage5	Human
MW512392	Singapore	2013	Lineage5	Human
MW512393	Singapore	2013	Lineage5	Human
MW512394	Singapore	2013	Lineage5	Human
MW512395	Singapore	2013	Lineage5	Human
MW512396	Singapore	2013	Lineage5	Human
MW512397	Singapore	2013	Lineage5	Human
MW512398	Singapore	2013	Lineage5	Human
MW512399	Singapore	2013	Lineage5	Human
MW512400	Singapore	2013	Lineage5	Human
MW512401	Singapore	2013	Lineage5	Human
MW512402	Singapore	2013	Lineage5	Human
MW512403	Singapore	2013	Lineage5	Human
MW512416	Singapore	2014	Lineage5	Human
MW512417	Singapore	2014	Lineage5	Human
MW512418	Singapore	2014	Lineage5	Human

MW512419	Singapore	2014	Lineage5	Human
MW512420	Singapore	2014	Lineage5	Human
MW512421	Singapore	2014	Lineage5	Human
MW512422	Singapore	2014	Lineage5	Human
MW512423	Singapore	2014	Lineage5	Human
MW512424	Singapore	2014	Lineage5	Human
MW512425	Singapore	2014	Lineage5	Human
MW512426	Singapore	2014	Lineage5	Human
MW512427	Singapore	2014	Lineage5	Human
MW512435	Singapore	2015	Lineage5	Human
MW512436	Singapore	2015	Lineage5	Human
MW512437	Singapore	2015	Lineage5	Human
MW512438	Singapore	2015	Lineage5	Human
MW512439	Singapore	2015	Lineage5	Human
MW512440	Singapore	2015	Lineage5	Human
MW512441	Singapore	2015	Lineage5	Human
MW512442	Singapore	2015	Lineage5	Human
MW512443	Singapore	2015	Lineage5	Human
MW512444	Singapore	2015	Lineage5	Human
MW512445	Singapore	2015	Lineage5	Human
MW512446	Singapore	2015	Lineage5	Human
MW512447	Singapore	2015	Lineage5	Human
MW512451	Singapore	2016	Lineage5	Human

MW512452	Singapore	2016	Lineage5	Human
MW512453	Singapore	2016	Lineage5	Human
MW512454	Singapore	2016	Lineage5	Human
MW512455	Singapore	2016	Lineage5	Human
MW512456	Singapore	2016	Lineage5	Human
MW512457	Singapore	2016	Lineage5	Human
MW512458	Singapore	2016	Lineage5	Human
MW512459	Singapore	2016	Lineage5	Human
MW512460	Singapore	2016	Lineage5	Human
MW512461	Singapore	2016	Lineage5	Human
MW512462	Singapore	2016	Lineage5	Human
MW512463	Singapore	2016	Lineage5	Human
MW512471	Singapore	2017	Lineage5	Human
MW512472	Singapore	2017	Lineage5	Human
MW512473	Singapore	2017	Lineage5	Human
MW512474	Singapore	2017	Lineage5	Human
MW512475	Singapore	2017	Lineage5	Human
MW512476	Singapore	2017	Lineage5	Human
MW512477	Singapore	2017	Lineage5	Human
MW512478	Singapore	2017	Lineage5	Human
MW512481	Singapore	2018	Lineage5	Human
MW512482	Singapore	2018	Lineage5	Human
MW512483	Singapore	2018	Lineage5	Human

MW512484	Singapore	2018	Lineage5	Human
MW512485	Singapore	2018	Lineage5	Human
MW512486	Singapore	2018	Lineage5	Human
MW512487	Singapore	2018	Lineage5	Human
MW512488	Singapore	2018	Lineage5	Human
MW512491	Singapore	2019	Lineage5	Human
MW512492	Singapore	2019	Lineage5	Human
MW512493	Singapore	2019	Lineage5	Human
MW512494	Singapore	2019	Lineage5	Human
MW512495	Singapore	2019	Lineage5	Human
MW512496	Singapore	2019	Lineage5	Human
MW512497	Singapore	2019	Lineage5	Human
MW512498	Singapore	2019	Lineage5	Human
MW510037	Singapore	2018	Lineage5	Mosquito
MW510038	Singapore	2018	Lineage5	Mosquito
MW510117	Singapore	2017	Lineage5	Mosquito
MW510118	Singapore	2017	Lineage5	Mosquito
MW510120	Singapore	2017	Lineage5	Mosquito
MW510992	Singapore	2015	Unclassified (Outgroup)	Human
MW510993	Singapore	2015	Unclassified (Outgroup)	Human
MW510989	Singapore	2012	Unclassified (Outgroup)	Human
MW510988	Singapore	2011	Unclassified (Outgroup)	Human
MW510991	Singapore	2015	Unclassified (Outgroup)	Human

MW510994	Singapore	2016	Unclassified (Outgroup)	Human
MW510990	Singapore	2014	Unclassified (Outgroup)	Human

Supplementary Table S2: Oligonucleotides used for the amplification and sequencing of DENV-2 genomes

Fragment	Primer name	Sequence (5' - 3')
Fragment 1	Den2 16F*	TGGACCGACAAAGACAGATTCTTTG
	Den2 348F	CAGGAAAGAGATTGGAAGGATGC
	Den2 738F	AATGGGACTGGAGACACGAACTG
	Den2 934R	TCATTGAAGGAGCGACAGC
	Den2 1169R*	CCTTGTGTTGGGCAGCGAGAT
Fragment 2	Den2 771F*	ACATGGATGTCRTCAGAAGG
	Den2 1353F	GTGATAACACCTCACTCAGGG
	Den2 1596R	CCATGGTAACGGCAGGTC
	Den2 2540R*	GAAGGGGATTCTGGTTGG
Fragment 3	Den2 2146F*	ACAACAATGAGAGGAGCGAAGAG
	Den2 2796F	CACAGAACTTCATAACCACACCT
	Den2 3222R	GGGTCCTGCCGTTTGTGTATGAT
	Den2 3865R	CTAAAGCCAACGCGTCAGTCAGT
	Den2 3545F	TCAGGACCCGAGTAGGAACGAA
	Den2 4590R*	ATAGGCTCCATCTTCCAGTTCAG
Fragment 4	Den2 4158F*	GGCAGTCGGGATGGTGAGC
	Den2 4539F	TGTCCCTTCACCCCCACCTGT
	Den2 4590R	ATAGGCTCCATCTTCCAGTTCAG

	Den2 4773R	CTTCCAGCCTCCTCCATACGATA
	Den2 5403R	TATGCTTGCTGGGTCTGTGAAAT
	Den2 5784R*	CCTCTCGGCCTTGAAGTTAGCAC
Fragment 5	Den2 5443F*	GAAGCAGCTGGGATTTTTATGAC
	Den2 6072R	GTCCACCTTTTCACGCTCTG
	Den2 6359F	TCGCAGCTGGAAGAAAGTC
	Den2 6776R	AATTGGTTATCTTGGGGTGTTC
	Den2 7161R	GAGAAGGGCTGCTGTGAGAGTTA
	Den2 7733R*	ACAGCGTGATGGTCCGTTTCT
Fragment 6	Den2 7416F*	AACCGGGCCCATCTCTACACTG
	Den2 7926F	CGGTTGGAATCTGGTGCGTCTT
	Den2 8181R	AGCTCCTCCGTATTTCCTTTGT
	Den2 8327F	AGCCAGATGTTGACCTCGGAAGT
	Den2 8847R	AACAGCCTCACGTGCCGACTT
	Den2 9154R*	CTCCTCCTTCTTTCTTACTCACG
Fragment 7	Den2 8662F*	GAACCGAAAGAAGGCACGAAAAA
	Den2 9599R	GCGCTTGCAAACCTGTCATCTAA
	Den2 8967F	CAAAGCAAAAGGCAGCAGAGC
	Den2 9821F	CAGCCTGTTTGGGGAAGTCT
	Den2 10321R*	GGCTTAATCCGACCTGACTTCTG

Amplification primers are denoted by asterisks. The remaining primers, together with amplification primers were used for sequencing.

Amplification protocol

**PCR conditions - Fragments 1 - 7**

**Temperature °C**	**Time**	**Cycle**
98 °C	10 sec	1
98 °C	5 sec	35
62 °C (F2 & F6)<br><br>65 °C (F1, F3, F4, F5 & F7)	10 sec	
72 °C	45 sec	
72 °C	2 min	1
20 °C	infinity	1

Each fragment was PCR amplified by using 0.5 µM of fragment-specific primers and 1X Phusion^{TM} Flash High-Fidelity PCR Master Mix (Thermo Fisher Scientific, MA, USA).

Supplementary Table S3: List of countries and the number of *E* gene sequences of each genotype of DENV-2 used in the analyses

Country Name	latitude	longitude	Number of sequences
American Samoa	-14.2756	-170.702	10
Australia	-35.3438	149.083	26
Bangladesh	23.77718	90.39945	12
Bhutan	26.87056	90.48556	2
Borneo	1.0343	114.33	2
Brunei	4.94029	114.9481	3
Burkina Faso	12.36566	-1.53388	6

China	39.91382	116.3636	518
Ireland	53.35014	-6.26615	2
Ivory Coast	5.345317	-4.02443	1
Fiji	-17.7134	178.065	14
France	48.86472	2.349014	1
French Polynesia	-16.7613	-151.444	12
Ghana	7.952771	-1.03071	1
Guam	13.4443	144.7937	1
India	28.4512	77.1646	137
Indonesia	-6.21462	106.8451	267
Japan	35.65283	139.8395	3
Kenya	-1.28042	36.81631	21
Maldives	1.924992	73.39966	3
Malaysia	3.140853	101.6932	180
Micronesia	6.887481	158.2151	3
New Caledonia	-21.2107	165.8517	13
Palau	7.51498	134.5825	1
Pakistan	33.73805	73.08449	52
Papua New Guinea	-6.47838	145.2417	25
Philippines	14.59951	120.9842	138
Saudi Arabia	24.77427	46.73859	11
Singapore	1.29027	103.852	1,691
Solomon Islands	-9.42635	160.1874	1

Somalia	2.046934	45.31816	5
South Korea	37.53260	127.0246	3
Sri Lanka	6.927079	79.86124	13
Taiwan	25.1055	121.5974	73
Tanzania	-6.16118	35.74543	3
Thailand	13.73672	100.5232	19
Timor Leste	-8.55686	125.5603	48
Tuvalu	-8.52115	179.1962	4
Uganda	0.347596	32.58252	1
Vietnam	10.76262	106.6602	36
Wallis	29.63414	-96.0639	5

Supplementary Table S4: Unique amino acid signature of Indian sub-continent lineage as compared to other lineages of cosmopolitan genotype and other non-sylvatic genotypes of DENV-2

Polyprotein position	Gene	Gene position	REF¶	Other genotypes	Indian lineage	Cosmo lineages (Excluding Indian lineage)
112	C	112	V	V/A	A (97%)	V (100%)
143	preM	29	D	D	D (100%)	N (100%)

332	E	52	Q	Q/E	Q (100%)	H (97%)
906	NS1	131	Q	Q	Q (100%)	H (98%)
1190	NS2A	63	T	T	A (97%)	T (97%)
1260	NS2A	133	V	V/A	I (96%)	V (98%)
1342	NS2A	215	S	S	N (99%)	S (99%)
1408	NS2B	63	D	D	D (100%)	E (98%)
1548	NS3	73	K	K	R (97%)	K (100%)
1748	NS3	273	V	V	V (100%)	I (98%)
1936	NS3	461	I	I	I (98%)	V (97%)
2487	NS4B	244	T	T	T (95%)	A (100%)
2589	NS5	98	R	R	R (98%)	K (98%)
3096	NS5	605	G	G	G (100%)	V (98%)
3107	NS5	616	E	E	G (98%)	E (100%)
3136	NS5	645	N	N	D (96%)	N (100%)

[¶]REF=NCBI refseq accession number NC_001474.2

Supplementary Figures

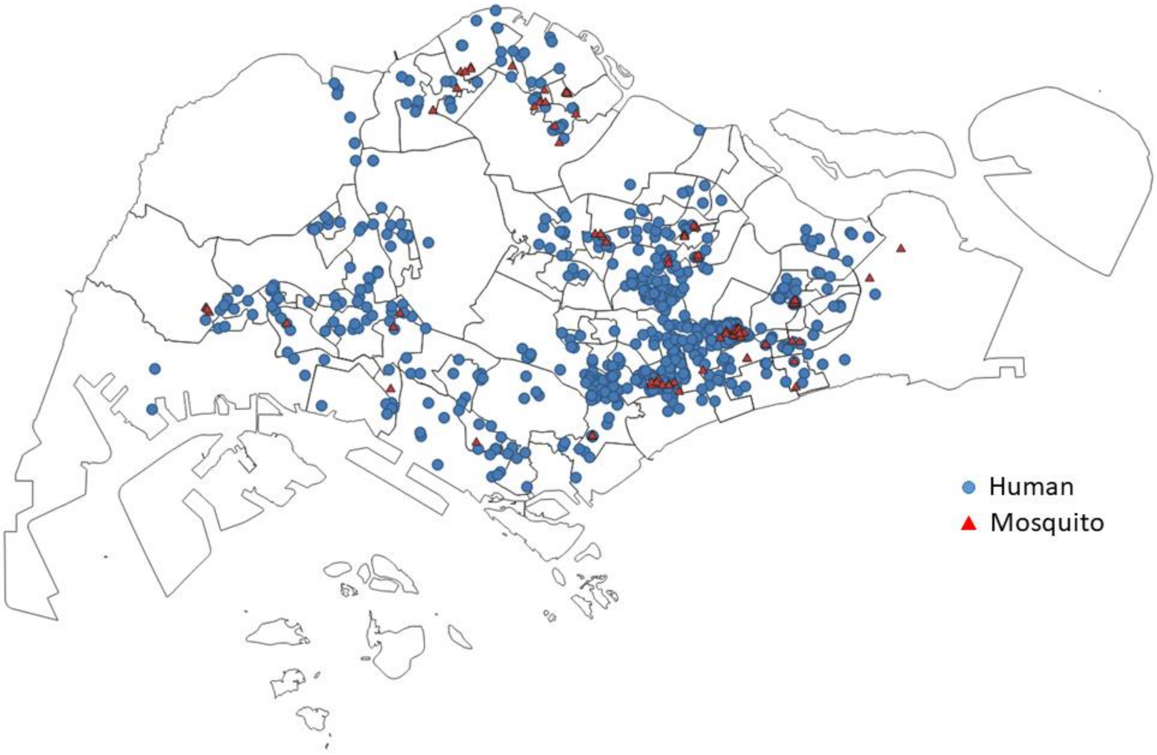

Supplementary Figure S1. Distribution of newly generated sequences obtained from human sera and field-caught mosquitoes. The map was generated by using the QGIS 3.16 open source (www.qgis.org) software and the layer obtained from the OneMap open source portal (www.datascoutonemap.com). Each sequence was mapped based on the postal codes of locations where respective human cases were detected and mosquitoes were captured.

Supplementary Figure S2. Bayesian phylogeny of DENV-2 complete coding sequences. This is an expansion view of Figure 3. The maximum clade credibility (MCC) tree was constructed in BEAST 1.10.4 version, using general time reversible (GTR) substitution model, relaxed random clock model and Bayesian skyline prior. The analysis included 380 complete coding sequences of the cosmopolitan genotype and a relatively small reference dataset to represent Asian I (n=10),

Asian II (n=10), Asian-American (n=8), American (n=8) and sylvatic (n=3) genotypes. Each genotype is colour coded as shown in the legend. The numbers at the nodes represent the posterior probability values. The time scale (in years) and scale bar (substitutions/site/year) are given at the bottom of the figure.

Supplementary Figure S3. Phylogenetic analysis of *E* gene sequences of cosmopolitan genotype. This is an expansion view of Figure 4. The neighbour joining tree was constructed by using 3,367 complete *E* gene sequences. The alignment included 2,319 sequences retrieved from NCBI database and 1,048 sequences generated during the present study. The taxa names of newly generated sequences are colour coded; those obtained from human sera in light blue and those derived from mosquitoes in red. Distinct lineages are shown in different colours. Sequences that were not classified into any distinct lineage are shown in black branches.
