# Supplementary figures and images for: Evolution, heterogeneity and global dispersal of cosmopolitan genotype of Dengue virus type 2
Source: Sci Rep. 2021 Jun 29;11:13496. doi: 10.1038/s41598-021-92783-y (PMC8241877; doi:10.1038/s41598-021-92783-y)

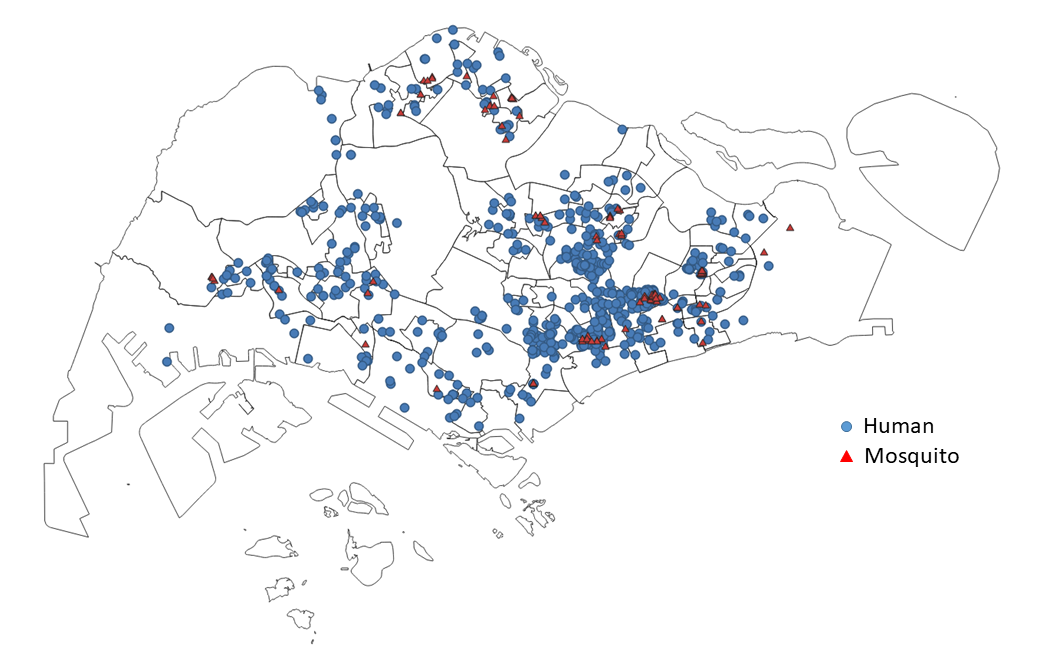

Supplement: Supplementary file 2 — Supplementary Figure S1. [file 41598_2021_92783_MOESM2_ESM.tif]

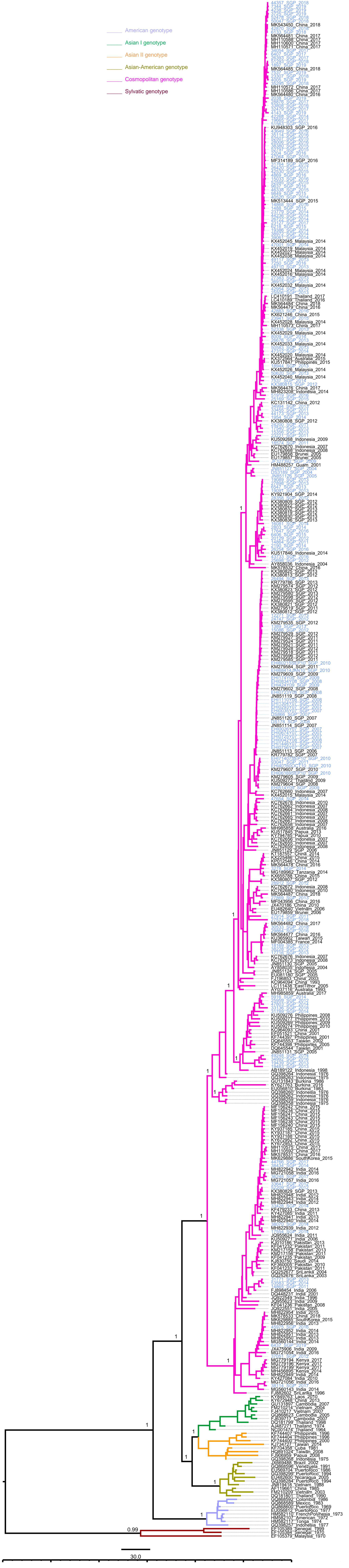

Supplement: Supplementary file 3 — Supplementary Figure S2. [file 41598_2021_92783_MOESM3_ESM.pdf]
