# Supplementary material for: Evolution, heterogeneity and global dispersal of cosmopolitan genotype of Dengue virus type 2
Source: Sci Rep. 2021 Jun 29;11:13496. doi: 10.1038/s41598-021-92783-y (PMC8241877; doi:10.1038/s41598-021-92783-y)
Supplement: Supplementary file 4 — Supplementary Figure S3. [file 41598_2021_92783_MOESM4_ESM.pdf]

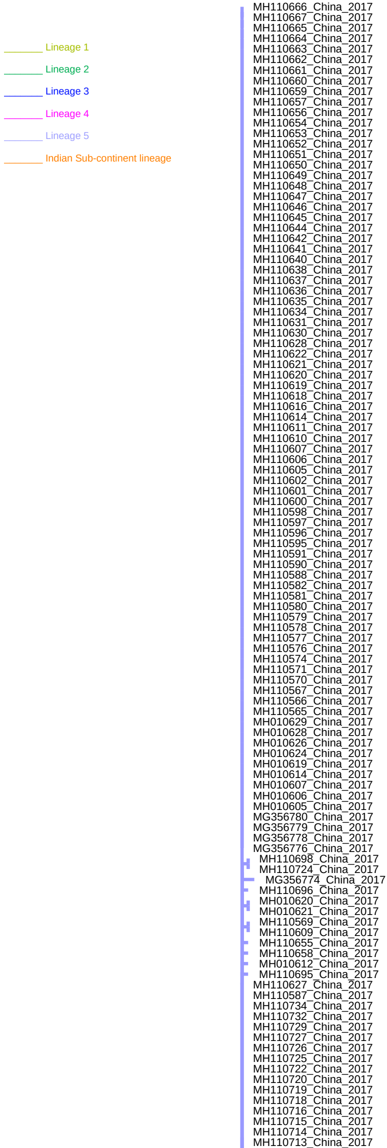
Lineage 1
Lineage 2
Lineage 3
Lineage 4
Lineage 5
Indian Sub-continent lineage
MH110666_China_2017
MH110667_China_2017
MH110665_China_2017
MH110664_China_2017
MH110663_China_2017
MH110662_China_2017
MH110661_China_2017
MH110660_China_2017
MH110659_China_2017
MH110657_China_2017
MH110656_China_2017
MH110654_China_2017
MH110653_China_2017
MH110652_China_2017
MH110651_China_2017
MH110650_China_2017
MH110649_China_2017
MH110648_China_2017
MH110647_China_2017
MH110646_China_2017
MH110645_China_2017
MH110644_China_2017
MH110642_China_2017
MH110641_China_2017
MH110640_China_2017
MH110638_China_2017
MH110637_China_2017
MH110636_China_2017
MH110635_China_2017
MH110634_China_2017
MH110631_China_2017
MH110630_China_2017
MH110628_China_2017
MH110622_China_2017
MH110621_China_2017
MH110620_China_2017
MH110619_China_2017
MH110618_China_2017
MH110616_China_2017
MH110614_China_2017
MH110611_China_2017
MH110610_China_2017
MH110607_China_2017
MH110606_China_2017
MH110605_China_2017
MH110602_China_2017
MH110601_China_2017
MH110600_China_2017
MH110598_China_2017
MH110597_China_2017
MH110596_China_2017
MH110595_China_2017
MH110591_China_2017
MH110590_China_2017
MH110588_China_2017
MH110582_China_2017
MH110581_China_2017
MH110580_China_2017
MH110579_China_2017
MH110578_China_2017
MH110577_China_2017
MH110576_China_2017
MH110574_China_2017
MH110571_China_2017
MH110570_China_2017
MH110567_China_2017
MH110566_China_2017
MH110565_China_2017
MH010629_China_2017
MH010628_China_2017
MH010626_China_2017
MH010624_China_2017
MH010619_China_2017
MH010614_China_2017
MH010607_China_2017
MH010606_China_2017
MH010605_China_2017
MG356780_China_2017
MG356779_China_2017
MG356778_China_2017
MG356776_China_2017
MH110698_China_2017
MH110724_China_2017
MG356774_China_2017
MH110696_China_2017
MH010620_China_2017
MH010621_China_2017
MH110569_China_2017
MH110609_China_2017
MH110655_China_2017
MH110658_China_2017
MH010612_China_2017
MH110695_China_2017
MH110627_China_2017
MH110587_China_2017
MH110734_China_2017
MH110732_China_2017
MH110729_China_2017
MH110727_China_2017
MH110726_China_2017
MH110725_China_2017
MH110722_China_2017
MH110720_China_2017
MH110719_China_2017
MH110718_China_2017
MH110716_China_2017
MH110715_China_2017
MH110714_China_2017
MH110713_China_2017

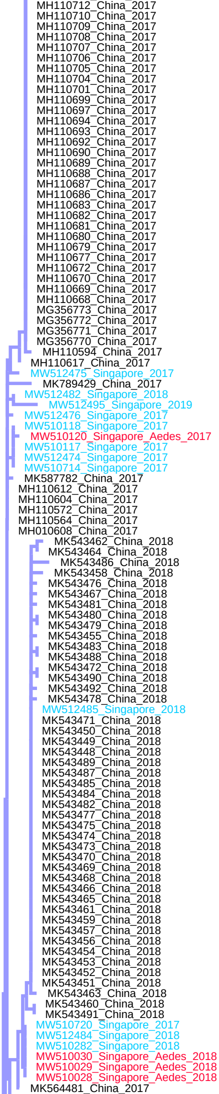
MH110712_China_2017
MH110710_China_2017
MH110709_China_2017
MH110708_China_2017
MH110707_China_2017
MH110706_China_2017
MH110705_China_2017
MH110704_China_2017
MH110701_China_2017
MH110699_China_2017
MH110697_China_2017
MH110694_China_2017
MH110693_China_2017
MH110692_China_2017
MH110690_China_2017
MH110689_China_2017
MH110688_China_2017
MH110687_China_2017
MH110686_China_2017
MH110683_China_2017
MH110682_China_2017
MH110681_China_2017
MH110680_China_2017
MH110679_China_2017
MH110677_China_2017
MH110672_China_2017
MH110670_China_2017
MH110669_China_2017
MH110668_China_2017
MG356773_China_2017
MG356772_China_2017
MG356771_China_2017
MG356770_China_2017
MH110594_China_2017
MH110617_China_2017
MW512475_Singapore_2017
MK789429_China_2017
MW512482_Singapore_2018
MW512495_Singapore_2019
MW512476_Singapore_2017
MW510118_Singapore_2017
MW510120_Singapore_Aedes_2017
MW510117_Singapore_2017
MW512474_Singapore_2017
MW510714_Singapore_2017
MK587782_China_2017
MH110612_China_2017
MH110604_China_2017
MH110572_China_2017
MH110564_China_2017
MH010608_China_2017
MK543462_China_2018
MK543464_China_2018
MK543486_China_2018
MK543458_China_2018
MK543476_China_2018
MK543467_China_2018
MK543481_China_2018
MK543480_China_2018
MK543479_China_2018
MK543455_China_2018
MK543483_China_2018
MK543488_China_2018
MK543472_China_2018
MK543490_China_2018
MK543492_China_2018
MK543478_China_2018
MW512485_Singapore_2018
MK543471_China_2018
MK543450_China_2018
MK543449_China_2018
MK543448_China_2018
MK543489_China_2018
MK543487_China_2018
MK543485_China_2018
MK543484_China_2018
MK543482_China_2018
MK543477_China_2018
MK543475_China_2018
MK543474_China_2018
MK543473_China_2018
MK543470_China_2018
MK543469_China_2018
MK543468_China_2018
MK543466_China_2018
MK543465_China_2018
MK543461_China_2018
MK543459_China_2018
MK543457_China_2018
MK543456_China_2018
MK543454_China_2018
MK543453_China_2018
MK543452_China_2018
MK543451_China_2018
MK543463_China_2018
MK543460_China_2018
MK543491_China_2018
MW510720_Singapore_2017
MW512484_Singapore_2018
MW510282_Singapore_2018
MW510030_Singapore_Aedes_2018
MW510029_Singapore_Aedes_2018
MW510028_Singapore_Aedes_2018
MK564481_China_2017

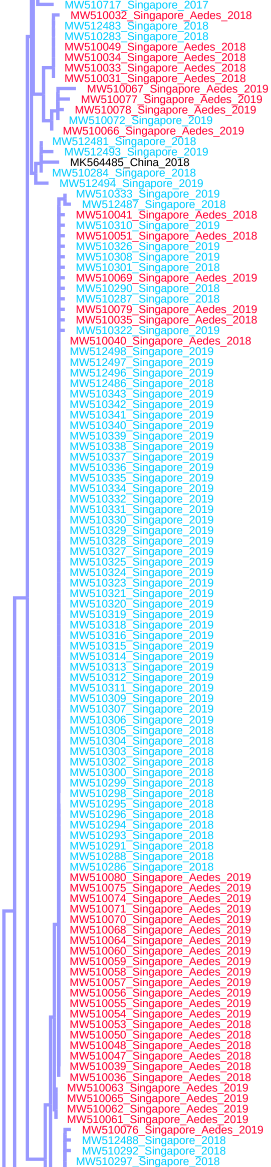
MW510717_Singapore_2017
MW510032_Singapore_Aedes_2018
MW512483_Singapore_2018
MW510283_Singapore_2018
MW510049_Singapore_Aedes_2018
MW510034_Singapore_Aedes_2018
MW510033_Singapore_Aedes_2018
MW510031_Singapore_Aedes_2018
MW510067_Singapore_Aedes_2019
MW510077_Singapore_Aedes_2019
MW510078_Singapore_Aedes_2019
MW510072_Singapore_2019
MW510066_Singapore_Aedes_2019
MW512481_Singapore_2018
MW512493_Singapore_2019
MK564485_China_2018
MW510284_Singapore_2018
MW512494_Singapore_2019
MW510333_Singapore_2019
MW512487_Singapore_2018
MW510041_Singapore_Aedes_2018
MW510310_Singapore_2019
MW510051_Singapore_Aedes_2018
MW510326_Singapore_2019
MW510308_Singapore_2019
MW510301_Singapore_2018
MW510069_Singapore_Aedes_2019
MW510290_Singapore_2018
MW510287_Singapore_2018
MW510079_Singapore_Aedes_2019
MW510035_Singapore_Aedes_2018
MW510322_Singapore_2019
MW510040_Singapore_Aedes_2018
MW512498_Singapore_2019
MW512497_Singapore_2019
MW512496_Singapore_2019
MW512486_Singapore_2018
MW510343_Singapore_2019
MW510342_Singapore_2019
MW510341_Singapore_2019
MW510340_Singapore_2019
MW510339_Singapore_2019
MW510338_Singapore_2019
MW510337_Singapore_2019
MW510336_Singapore_2019
MW510335_Singapore_2019
MW510334_Singapore_2019
MW510332_Singapore_2019
MW510331_Singapore_2019
MW510330_Singapore_2019
MW510329_Singapore_2019
MW510328_Singapore_2019
MW510327_Singapore_2019
MW510325_Singapore_2019
MW510324_Singapore_2019
MW510323_Singapore_2019
MW510321_Singapore_2019
MW510320_Singapore_2019
MW510319_Singapore_2019
MW510318_Singapore_2019
MW510316_Singapore_2019
MW510315_Singapore_2019
MW510314_Singapore_2019
MW510313_Singapore_2019
MW510312_Singapore_2019
MW510311_Singapore_2019
MW510309_Singapore_2019
MW510307_Singapore_2019
MW510306_Singapore_2019
MW510305_Singapore_2018
MW510304_Singapore_2018
MW510303_Singapore_2018
MW510302_Singapore_2018
MW510300_Singapore_2018
MW510299_Singapore_2018
MW510298_Singapore_2018
MW510295_Singapore_2018
MW510296_Singapore_2018
MW510294_Singapore_2018
MW510293_Singapore_2018
MW510291_Singapore_2018
MW510288_Singapore_2018
MW510286_Singapore_2018
MW510080_Singapore_Aedes_2019
MW510075_Singapore_Aedes_2019
MW510074_Singapore_Aedes_2019
MW510071_Singapore_Aedes_2019
MW510070_Singapore_Aedes_2019
MW510068_Singapore_Aedes_2019
MW510064_Singapore_Aedes_2019
MW510060_Singapore_Aedes_2019
MW510059_Singapore_Aedes_2019
MW510058_Singapore_Aedes_2019
MW510057_Singapore_Aedes_2019
MW510056_Singapore_Aedes_2019
MW510055_Singapore_Aedes_2019
MW510054_Singapore_Aedes_2019
MW510053_Singapore_Aedes_2018
MW510050_Singapore_Aedes_2018
MW510048_Singapore_Aedes_2018
MW510047_Singapore_Aedes_2018
MW510039_Singapore_Aedes_2018
MW510036_Singapore_Aedes_2018
MW510063_Singapore_Aedes_2019
MW510065_Singapore_Aedes_2019
MW510062_Singapore_Aedes_2019
MW510061_Singapore_Aedes_2019
MW510076_Singapore_Aedes_2019
MW512488_Singapore_2018
MW510292_Singapore_2018
MW510297_Singapore_2018

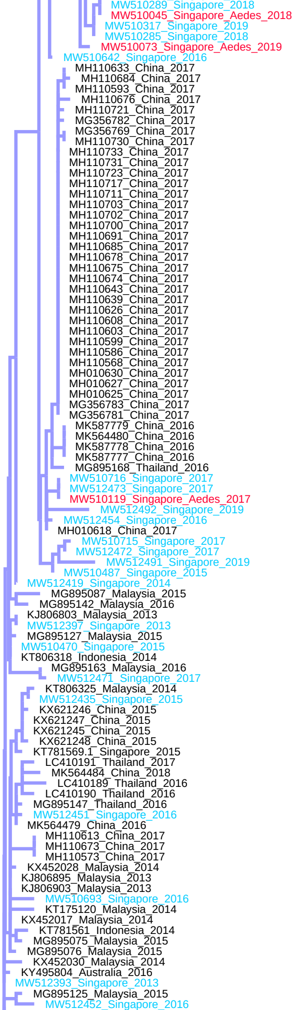
MW510289_Singapore_2018
MW510045_Singapore_Aedes_2018
MW510317_Singapore_2019
MW510285_Singapore_2018
MW510073_Singapore_Aedes_2019
MW510642_Singapore_2016
MH110633_China_2017
MH110684_China_2017
MH110593_China_2017
MH110676_China_2017
MH110721_China_2017
MG356782_China_2017
MG356769_China_2017
MH110730_China_2017
MH110733_China_2017
MH110731_China_2017
MH110723_China_2017
MH110717_China_2017
MH110711_China_2017
MH110703_China_2017
MH110702_China_2017
MH110700_China_2017
MH110691_China_2017
MH110685_China_2017
MH110678_China_2017
MH110675_China_2017
MH110674_China_2017
MH110643_China_2017
MH110639_China_2017
MH110626_China_2017
MH110608_China_2017
MH110603_China_2017
MH110599_China_2017
MH110586_China_2017
MH110568_China_2017
MH010630_China_2017
MH010627_China_2017
MH010625_China_2017
MG356783_China_2017
MG356781_China_2017
MK587779_China_2016
MK564480_China_2016
MK587778_China_2016
MK587777_China_2016
MG895168_Thailand_2016
MW510716_Singapore_2017
MW512473_Singapore_2017
MW510119_Singapore_Aedes_2017
MW512492_Singapore_2019
MW512454_Singapore_2016
MH010618_China_2017
MW510715_Singapore_2017
MW512472_Singapore_2017
MW512491_Singapore_2019
MW510487_Singapore_2015
MW512419_Singapore_2014
MG895087_Malaysia_2015
MG895142_Malaysia_2016
KJ806803_Malaysia_2013
MW512397_Singapore_2013
MG895127_Malaysia_2015
MW510470_Singapore_2015
KT806318_Indonesia_2014
MG895163_Malaysia_2016
MW512471_Singapore_2017
KT806325_Malaysia_2014
MW512435_Singapore_2015
KX621246_China_2015
KX621247_China_2015
KX621245_China_2015
KX621248_China_2015
KT781569.1_Singapore_2015
LC410191_Thailand_2017
MK564484_China_2018
LC410189_Thailand_2016
LC410190_Thailand_2016
MG895147_Thailand_2016
MW512451_Singapore_2016
MK564479_China_2016
MH110613_China_2017
MH110673_China_2017
MH110573_China_2017
KX452028_Malaysia_2014
KJ806895_Malaysia_2013
KJ806903_Malaysia_2013
MW510693_Singapore_2016
KT175120_Malaysia_2014
KX452017_Malaysia_2014
KT781561_Indonesia_2014
MG895075_Malaysia_2015
MG895076_Malaysia_2015
KX452030_Malaysia_2014
KY495804_Australia_2016
MW512393_Singapore_2013
MG895125_Malaysia_2015
MW512452_Singapore_2016

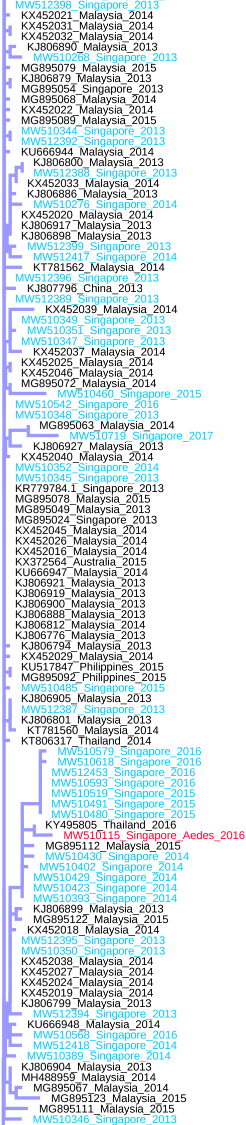
MW512398_Singapore_2013
KX452021_Malaysia_2014
KX452031_Malaysia_2014
KX452032_Malaysia_2014
KJ806890_Malaysia_2013
MW510268_Singapore_2013
MG895079_Malaysia_2015
KJ806879_Malaysia_2013
MG895054_Singapore_2013
MG895068_Malaysia_2014
KX452022_Malaysia_2014
MG895089_Malaysia_2015
MW510344_Singapore_2013
MW512392_Singapore_2013
KU666944_Malaysia_2014
KJ806800_Malaysia_2013
MW512388_Singapore_2013
KX452033_Malaysia_2014
KJ806886_Malaysia_2013
MW510276_Singapore_2014
KX452020_Malaysia_2014
KJ806917_Malaysia_2013
KJ806898_Malaysia_2013
MW512399_Singapore_2013
MW512417_Singapore_2014
KT781562_Malaysia_2014
MW512396_Singapore_2013
KJ807796_China_2013
MW512389_Singapore_2013
KX452039_Malaysia_2014
MW510349_Singapore_2013
MW510351_Singapore_2013
MW510347_Singapore_2013
KX452037_Malaysia_2014
KX452025_Malaysia_2014
KX452046_Malaysia_2014
MG895072_Malaysia_2014
MW510460_Singapore_2015
MW510542_Singapore_2016
MW510348_Singapore_2013
MG895063_Malaysia_2014
MW510719_Singapore_2017
KJ806927_Malaysia_2013
KX452040_Malaysia_2014
MW510352_Singapore_2014
MW510345_Singapore_2013
KR779784.1_Singapore_2013
MG895078_Malaysia_2015
MG895049_Malaysia_2013
MG895024_Singapore_2013
KX452045_Malaysia_2014
KX452026_Malaysia_2014
KX452016_Malaysia_2014
KX372564_Australia_2015
KU666947_Malaysia_2014
KJ806921_Malaysia_2013
KJ806919_Malaysia_2013
KJ806900_Malaysia_2013
KJ806888_Malaysia_2013
KJ806812_Malaysia_2014
KJ806776_Malaysia_2013
KJ806794_Malaysia_2013
KX452029_Malaysia_2014
KU517847_Philippines_2015
MG895092_Philippines_2015
MW510485_Singapore_2015
KJ806905_Malaysia_2013
MW512387_Singapore_2013
KJ806801_Malaysia_2013
KT781560_Malaysia_2014
KT806317_Thailand_2014
MW510579_Singapore_2016
MW510618_Singapore_2016
MW512453_Singapore_2016
MW510593_Singapore_2016
MW510519_Singapore_2015
MW510491_Singapore_2015
MW510480_Singapore_2015
KY495805_Thailand_2016
MW510115_Singapore_Aedes_2016
MG895112_Malaysia_2015
MW510430_Singapore_2014
MW510402_Singapore_2014
MW510429_Singapore_2014
MW510423_Singapore_2014
MW510393_Singapore_2014
KJ806899_Malaysia_2013
MG895122_Malaysia_2015
KX452018_Malaysia_2014
MW512395_Singapore_2013
MW510350_Singapore_2013
KX452038_Malaysia_2014
KX452027_Malaysia_2014
KX452024_Malaysia_2014
KX452019_Malaysia_2014
KJ806799_Malaysia_2013
MW512394_Singapore_2013
KU666948_Malaysia_2014
MW510568_Singapore_2016
MW512418_Singapore_2014
MW510389_Singapore_2014
KJ806904_Malaysia_2013
MH488959_Malaysia_2014
MG895067_Malaysia_2014
MG895123_Malaysia_2015
MG895111_Malaysia_2015
MW510346_Singapore_2013

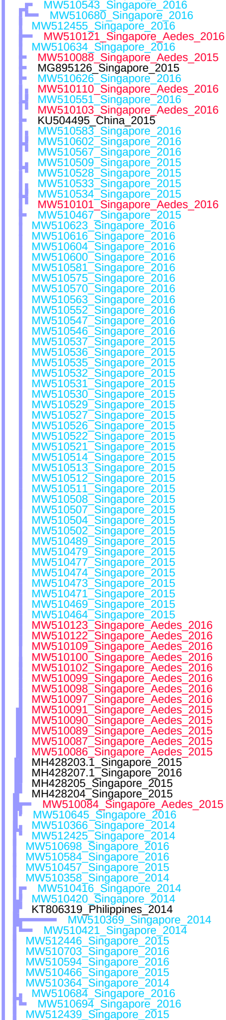
MW510543_Singapore_2016
MW510680_Singapore_2016
MW512455_Singapore_2016
MW510121_Singapore_Aedes_2016
MW510634_Singapore_2016
MW510088_Singapore_Aedes_2015
MG895126_Singapore_2015
MW510626_Singapore_2016
MW510110_Singapore_Aedes_2016
MW510551_Singapore_2016
MW510103_Singapore_Aedes_2016
KU504495_China_2015
MW510583_Singapore_2016
MW510602_Singapore_2016
MW510567_Singapore_2016
MW510509_Singapore_2015
MW510528_Singapore_2015
MW510533_Singapore_2015
MW510534_Singapore_2015
MW510101_Singapore_Aedes_2016
MW510467_Singapore_2015
MW510623_Singapore_2016
MW510616_Singapore_2016
MW510604_Singapore_2016
MW510600_Singapore_2016
MW510581_Singapore_2016
MW510575_Singapore_2016
MW510570_Singapore_2016
MW510563_Singapore_2016
MW510552_Singapore_2016
MW510547_Singapore_2016
MW510546_Singapore_2016
MW510537_Singapore_2015
MW510536_Singapore_2015
MW510535_Singapore_2015
MW510532_Singapore_2015
MW510531_Singapore_2015
MW510530_Singapore_2015
MW510529_Singapore_2015
MW510527_Singapore_2015
MW510526_Singapore_2015
MW510522_Singapore_2015
MW510521_Singapore_2015
MW510514_Singapore_2015
MW510513_Singapore_2015
MW510512_Singapore_2015
MW510511_Singapore_2015
MW510508_Singapore_2015
MW510507_Singapore_2015
MW510504_Singapore_2015
MW510502_Singapore_2015
MW510489_Singapore_2015
MW510479_Singapore_2015
MW510477_Singapore_2015
MW510474_Singapore_2015
MW510473_Singapore_2015
MW510471_Singapore_2015
MW510469_Singapore_2015
MW510464_Singapore_2015
MW510123_Singapore_Aedes_2016
MW510122_Singapore_Aedes_2016
MW510109_Singapore_Aedes_2016
MW510100_Singapore_Aedes_2016
MW510102_Singapore_Aedes_2016
MW510099_Singapore_Aedes_2016
MW510098_Singapore_Aedes_2016
MW510097_Singapore_Aedes_2016
MW510091_Singapore_Aedes_2015
MW510090_Singapore_Aedes_2015
MW510089_Singapore_Aedes_2015
MW510087_Singapore_Aedes_2015
MW510086_Singapore_Aedes_2015
MH428203.1_Singapore_2015
MH428207.1_Singapore_2016
MH428205_Singapore_2015
MH428204_Singapore_2015
MW510084_Singapore_Aedes_2015
MW510645_Singapore_2016
MW510366_Singapore_2014
MW512425_Singapore_2014
MW510698_Singapore_2016
MW510584_Singapore_2016
MW510457_Singapore_2015
MW510358_Singapore_2014
MW510416_Singapore_2014
MW510420_Singapore_2014
KT806319_Philippines_2014
MW510369_Singapore_2014
MW510421_Singapore_2014
MW512446_Singapore_2015
MW510703_Singapore_2016
MW510594_Singapore_2016
MW510466_Singapore_2015
MW510364_Singapore_2014
MW510684_Singapore_2016
MW510694_Singapore_2016
MW512439_Singapore_2015

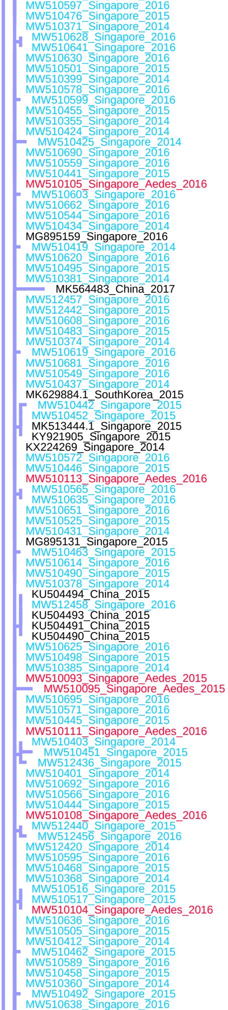

MW510597_Singapore_2016
MW510476_Singapore_2015
MW510371_Singapore_2014
MW510628_Singapore_2016
MW510641_Singapore_2016
MW510630_Singapore_2016
MW510501_Singapore_2015
MW510399_Singapore_2014
MW510578_Singapore_2016
MW510599_Singapore_2016
MW510455_Singapore_2015
MW510355_Singapore_2014
MW510424_Singapore_2014
MW510425_Singapore_2014
MW510690_Singapore_2016
MW510559_Singapore_2016
MW510441_Singapore_2015
MW510105_Singapore_Aedes_2016
MW510603_Singapore_2016
MW510662_Singapore_2016
MW510544_Singapore_2016
MW510434_Singapore_2014
MG895159_Singapore_2016
MW510419_Singapore_2014
MW510620_Singapore_2016
MW510495_Singapore_2015
MW510381_Singapore_2014
MK564483_China_2017
MW512457_Singapore_2016
MW512442_Singapore_2015
MW510608_Singapore_2016
MW510483_Singapore_2015
MW510374_Singapore_2014
MW510619_Singapore_2016
MW510681_Singapore_2016
MW510549_Singapore_2016
MW510437_Singapore_2014
MK629884.1_SouthKorea_2015
MW510442_Singapore_2015
MW510452_Singapore_2015
MK513444.1_Singapore_2015
KY921905_Singapore_2015
KX224269_Singapore_2014
MW510572_Singapore_2016
MW510446_Singapore_2015
MW510113_Singapore_Aedes_2016
MW510565_Singapore_2016
MW510635_Singapore_2016
MW510651_Singapore_2016
MW510525_Singapore_2015
MW510431_Singapore_2014
MG895131_Singapore_2015
MW510463_Singapore_2015
MW510614_Singapore_2016
MW510490_Singapore_2015
MW510378_Singapore_2014
KU504494_China_2015
MW512458_Singapore_2016
KU504493_China_2015
KU504491_China_2015
KU504490_China_2015
MW510625_Singapore_2016
MW510498_Singapore_2015
MW510385_Singapore_2014
MW510093_Singapore_Aedes_2015
MW510095_Singapore_Aedes_2015
MW510695_Singapore_2016
MW510571_Singapore_2016
MW510445_Singapore_2015
MW510111_Singapore_Aedes_2016
MW510403_Singapore_2014
MW510451_Singapore_2015
MW512436_Singapore_2015
MW510401_Singapore_2014
MW510692_Singapore_2016
MW510566_Singapore_2016
MW510444_Singapore_2015
MW510108_Singapore_Aedes_2016
MW512440_Singapore_2015
MW512456_Singapore_2016
MW512420_Singapore_2014
MW510595_Singapore_2016
MW510468_Singapore_2015
MW510368_Singapore_2014
MW510516_Singapore_2015
MW510517_Singapore_2015
MW510104_Singapore_Aedes_2016
MW510636_Singapore_2016
MW510505_Singapore_2015
MW510412_Singapore_2014
MW510462_Singapore_2015
MW510589_Singapore_2016
MW510458_Singapore_2015
MW510360_Singapore_2014
MW510492_Singapore_2015
MW510638_Singapore_2016

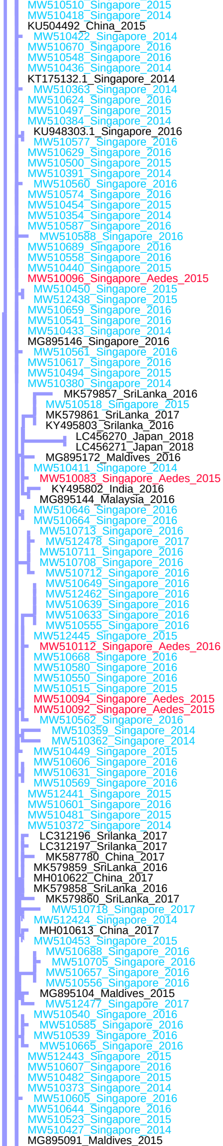
MW510510_Singapore_2015
MW510418_Singapore_2014
KU504492_China_2015
MW510422_Singapore_2014
MW510670_Singapore_2016
MW510548_Singapore_2016
MW510436_Singapore_2014
KT175132.1_Singapore_2014
MW510363_Singapore_2014
MW510624_Singapore_2016
MW510497_Singapore_2015
MW510384_Singapore_2014
KU948303.1_Singapore_2016
MW510577_Singapore_2016
MW510629_Singapore_2016
MW510500_Singapore_2015
MW510391_Singapore_2014
MW510560_Singapore_2016
MW510574_Singapore_2016
MW510454_Singapore_2015
MW510354_Singapore_2014
MW510587_Singapore_2016
MW510588_Singapore_2016
MW510689_Singapore_2016
MW510558_Singapore_2016
MW510440_Singapore_2015
MW510096_Singapore_Aedes_2015
MW510450_Singapore_2015
MW512438_Singapore_2015
MW510659_Singapore_2016
MW510541_Singapore_2016
MW510433_Singapore_2014
MG895146_Singapore_2016
MW510561_Singapore_2016
MW510617_Singapore_2016
MW510494_Singapore_2015
MW510380_Singapore_2014
MK579857_SriLanka_2016
MW510518_Singapore_2015
MK579861_SriLanka_2017
KY495803_Srilanka_2016
LC456270_Japan_2018
LC456271_Japan_2018
MG895172_Maldives_2016
MW510411_Singapore_2014
MW510083_Singapore_Aedes_2015
KY495802_India_2016
MG895144_Malaysia_2016
MW510646_Singapore_2016
MW510664_Singapore_2016
MW510713_Singapore_2016
MW512478_Singapore_2017
MW510711_Singapore_2016
MW510708_Singapore_2016
MW510712_Singapore_2016
MW510649_Singapore_2016
MW512462_Singapore_2016
MW510639_Singapore_2016
MW510633_Singapore_2016
MW510555_Singapore_2016
MW512445_Singapore_2015
MW510112_Singapore_Aedes_2016
MW510668_Singapore_2016
MW510580_Singapore_2016
MW510550_Singapore_2016
MW510515_Singapore_2015
MW510094_Singapore_Aedes_2015
MW510092_Singapore_Aedes_2015
MW510562_Singapore_2016
MW510359_Singapore_2014
MW510362_Singapore_2014
MW510449_Singapore_2015
MW510606_Singapore_2016
MW510631_Singapore_2016
MW510569_Singapore_2016
MW512441_Singapore_2015
MW510601_Singapore_2016
MW510481_Singapore_2015
MW510372_Singapore_2014
LC312196_Srilanka_2017
LC312197_Srilanka_2017
MK587780_China_2017
MK579859_SriLanka_2016
MH010622_China_2017
MK579858_SriLanka_2016
MK579860_SriLanka_2017
MW510718_Singapore_2017
MW512424_Singapore_2014
MH010613_China_2017
MW510453_Singapore_2015
MW510688_Singapore_2016
MW510705_Singapore_2016
MW510657_Singapore_2016
MW510556_Singapore_2016
MG895104_Maldives_2015
MW512477_Singapore_2017
MW510540_Singapore_2016
MW510585_Singapore_2016
MW510539_Singapore_2016
MW510665_Singapore_2016
MW512443_Singapore_2015
MW510607_Singapore_2016
MW510482_Singapore_2015
MW510373_Singapore_2014
MW510605_Singapore_2016
MW510644_Singapore_2016
MW510523_Singapore_2015
MW510427_Singapore_2014
MG895091_Maldives_2015

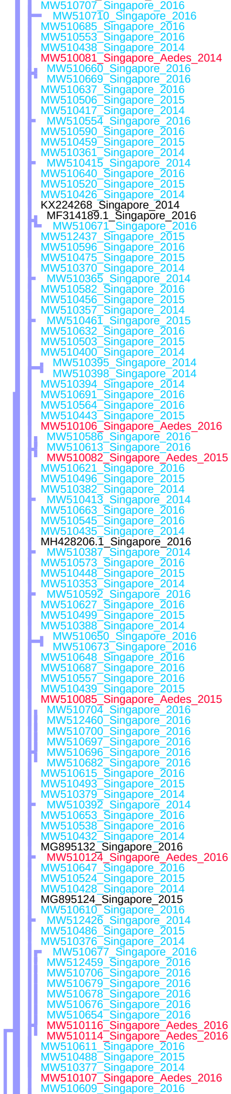
MW510707_Singapore_2016
MW510710_Singapore_2016
MW510685_Singapore_2016
MW510553_Singapore_2016
MW510438_Singapore_2014
MW510081_Singapore_Aedes_2014
MW510660_Singapore_2016
MW510669_Singapore_2016
MW510637_Singapore_2016
MW510506_Singapore_2015
MW510417_Singapore_2014
MW510554_Singapore_2016
MW510590_Singapore_2016
MW510459_Singapore_2015
MW510361_Singapore_2014
MW510415_Singapore_2014
MW510640_Singapore_2016
MW510520_Singapore_2015
MW510426_Singapore_2014
KX224268_Singapore_2014
MF314189.1_Singapore_2016
MW510671_Singapore_2016
MW512437_Singapore_2015
MW510596_Singapore_2016
MW510475_Singapore_2015
MW510370_Singapore_2014
MW510365_Singapore_2014
MW510582_Singapore_2016
MW510456_Singapore_2015
MW510357_Singapore_2014
MW510461_Singapore_2015
MW510632_Singapore_2016
MW510503_Singapore_2015
MW510400_Singapore_2014
MW510395_Singapore_2014
MW510398_Singapore_2014
MW510394_Singapore_2014
MW510691_Singapore_2016
MW510564_Singapore_2016
MW510443_Singapore_2015
MW510106_Singapore_Aedes_2016
MW510586_Singapore_2016
MW510613_Singapore_2016
MW510082_Singapore_Aedes_2015
MW510621_Singapore_2016
MW510496_Singapore_2015
MW510382_Singapore_2014
MW510413_Singapore_2014
MW510663_Singapore_2016
MW510545_Singapore_2016
MW510435_Singapore_2014
MH428206.1_Singapore_2016
MW510387_Singapore_2014
MW510573_Singapore_2016
MW510448_Singapore_2015
MW510353_Singapore_2014
MW510592_Singapore_2016
MW510627_Singapore_2016
MW510499_Singapore_2015
MW510388_Singapore_2014
MW510650_Singapore_2016
MW510673_Singapore_2016
MW510648_Singapore_2016
MW510687_Singapore_2016
MW510557_Singapore_2016
MW510439_Singapore_2015
MW510085_Singapore_Aedes_2015
MW510704_Singapore_2016
MW512460_Singapore_2016
MW510700_Singapore_2016
MW510697_Singapore_2016
MW510696_Singapore_2016
MW510682_Singapore_2016
MW510615_Singapore_2016
MW510493_Singapore_2015
MW510379_Singapore_2014
MW510392_Singapore_2014
MW510653_Singapore_2016
MW510538_Singapore_2016
MW510432_Singapore_2014
MG895132_Singapore_2016
MW510124_Singapore_Aedes_2016
MW510647_Singapore_2016
MW510524_Singapore_2015
MW510428_Singapore_2014
MG895124_Singapore_2015
MW510610_Singapore_2016
MW512426_Singapore_2014
MW510486_Singapore_2015
MW510376_Singapore_2014
MW510677_Singapore_2016
MW512459_Singapore_2016
MW510706_Singapore_2016
MW510679_Singapore_2016
MW510678_Singapore_2016
MW510676_Singapore_2016
MW510654_Singapore_2016
MW510116_Singapore_Aedes_2016
MW510114_Singapore_Aedes_2016
MW510611_Singapore_2016
MW510488_Singapore_2015
MW510377_Singapore_2014
MW510107_Singapore_Aedes_2016
MW510609_Singapore_2016

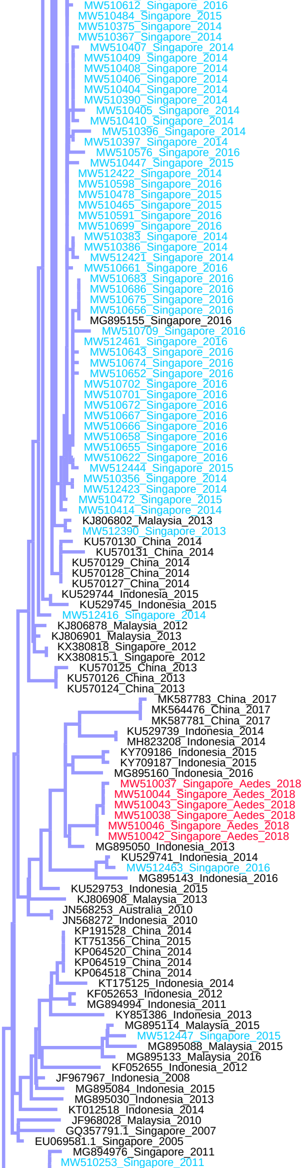
MW510612_Singapore_2016
MW510484_Singapore_2015
MW510375_Singapore_2014
MW510367_Singapore_2014
MW510407_Singapore_2014
MW510409_Singapore_2014
MW510408_Singapore_2014
MW510406_Singapore_2014
MW510404_Singapore_2014
MW510390_Singapore_2014
MW510405_Singapore_2014
MW510410_Singapore_2014
MW510396_Singapore_2014
MW510397_Singapore_2014
MW510576_Singapore_2016
MW510447_Singapore_2015
MW512422_Singapore_2014
MW510598_Singapore_2016
MW510478_Singapore_2015
MW510465_Singapore_2015
MW510591_Singapore_2016
MW510699_Singapore_2016
MW510383_Singapore_2014
MW510386_Singapore_2014
MW512421_Singapore_2014
MW510661_Singapore_2016
MW510683_Singapore_2016
MW510686_Singapore_2016
MW510675_Singapore_2016
MW510656_Singapore_2016
MG895155_Singapore_2016
MW510709_Singapore_2016
MW512461_Singapore_2016
MW510643_Singapore_2016
MW510674_Singapore_2016
MW510652_Singapore_2016
MW510702_Singapore_2016
MW510701_Singapore_2016
MW510672_Singapore_2016
MW510667_Singapore_2016
MW510666_Singapore_2016
MW510658_Singapore_2016
MW510655_Singapore_2016
MW510622_Singapore_2016
MW512444_Singapore_2015
MW510356_Singapore_2014
MW512423_Singapore_2014
MW510472_Singapore_2015
MW510414_Singapore_2014
KJ806802_Malaysia_2013
MW512390_Singapore_2013
KU570130_China_2014
KU570131_China_2014
KU570129_China_2014
KU570128_China_2014
KU570127_China_2014
KU529744_Indonesia_2015
KU529745_Indonesia_2015
MW512416_Singapore_2014
KJ806878_Malaysia_2012
KJ806901_Malaysia_2013
KX380818_Singapore_2012
KX380815.1_Singapore_2012
KU570125_China_2013
KU570126_China_2013
KU570124_China_2013
MK587783_China_2017
MK564476_China_2017
MK587781_China_2017
KU529739_Indonesia_2014
MH823208_Indonesia_2014
KY709186_Indonesia_2015
KY709187_Indonesia_2015
MG895160_Indonesia_2016
MW510037_Singapore_Aedes_2018
MW510044_Singapore_Aedes_2018
MW510043_Singapore_Aedes_2018
MW510038_Singapore_Aedes_2018
MW510046_Singapore_Aedes_2018
MW510042_Singapore_Aedes_2018
MG895050_Indonesia_2013
KU529741_Indonesia_2014
MW512463_Singapore_2016
MG895143_Indonesia_2016
KU529753_Indonesia_2015
KJ806908_Malaysia_2013
JN568253_Australia_2010
JN568272_Indonesia_2010
KP191528_China_2014
KT751356_China_2015
KP064520_China_2014
KP064519_China_2014
KP064518_China_2014
KT175125_Indonesia_2014
KF052653_Indonesia_2012
MG894994_Indonesia_2011
KY851386_Indonesia_2013
MG895114_Malaysia_2015
MW512447_Singapore_2015
MG895088_Malaysia_2015
MG895133_Malaysia_2016
KF052655_Indonesia_2012
JF967967_Indonesia_2008
MG895084_Indonesia_2015
MG895030_Indonesia_2013
KT012518_Indonesia_2014
JF968028_Malaysia_2010
GQ357791.1_Singapore_2007
EU069581.1_Singapore_2005
MG894976_Singapore_2011
MW510253_Singapore_2011

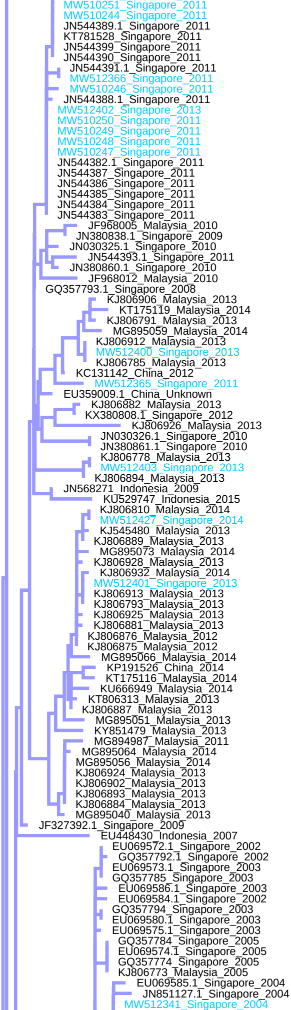
MW510251_Singapore_2011
MW510244_Singapore_2011
JN544389.1_Singapore_2011
KT781528_Singapore_2011
JN544399_Singapore_2011
JN544390_Singapore_2011
JN544391.1_Singapore_2011
MW512366_Singapore_2011
MW510246_Singapore_2011
JN544388.1_Singapore_2011
MW512402_Singapore_2013
MW510250_Singapore_2011
MW510249_Singapore_2011
MW510248_Singapore_2011
MW510247_Singapore_2011
JN544382.1_Singapore_2011
JN544387_Singapore_2011
JN544386_Singapore_2011
JN544385_Singapore_2011
JN544384_Singapore_2011
JN544383_Singapore_2011
JF968005_Malaysia_2010
JN380838.1_Singapore_2009
JN030325.1_Singapore_2010
JN544393.1_Singapore_2011
JN380860.1_Singapore_2010
JF968012_Malaysia_2010
GQ357793.1_Singapore_2008
KJ806906_Malaysia_2013
KT175119_Malaysia_2014
KJ806791_Malaysia_2013
MG895059_Malaysia_2014
KJ806912_Malaysia_2013
MW512400_Singapore_2013
KJ806785_Malaysia_2013
KC131142_China_2012
MW512365_Singapore_2011
EU359009.1_China_Unknown
KJ806882_Malaysia_2013
KX380808.1_Singapore_2012
KJ806926_Malaysia_2013
JN030326.1_Singapore_2010
JN380861.1_Singapore_2010
KJ806778_Malaysia_2013
MW512403_Singapore_2013
KJ806894_Malaysia_2013
JN568271_Indonesia_2009
KU529747_Indonesia_2015
KJ806810_Malaysia_2014
MW512427_Singapore_2014
KJ545480_Malaysia_2013
KJ806889_Malaysia_2013
MG895073_Malaysia_2014
KJ806928_Malaysia_2013
KJ806932_Malaysia_2014
MW512401_Singapore_2013
KJ806913_Malaysia_2013
KJ806793_Malaysia_2013
KJ806925_Malaysia_2013
KJ806881_Malaysia_2013
KJ806876_Malaysia_2012
KJ806875_Malaysia_2012
MG895066_Malaysia_2014
KP191526_China_2014
KT175116_Malaysia_2014
KU666949_Malaysia_2014
KT806313_Malaysia_2013
KJ806887_Malaysia_2013
MG895051_Malaysia_2013
KY851479_Malaysia_2013
MG894987_Malaysia_2011
MG895064_Malaysia_2014
MG895056_Malaysia_2014
KJ806924_Malaysia_2013
KJ806902_Malaysia_2013
KJ806893_Malaysia_2013
KJ806884_Malaysia_2013
MG895040_Malaysia_2013
JF327392.1_Singapore_2009
EU448430_Indonesia_2007
EU069572.1_Singapore_2002
GQ357792.1_Singapore_2002
EU069573.1_Singapore_2003
GQ357785_Singapore_2003
EU069586.1_Singapore_2003
EU069584.1_Singapore_2002
GQ357794_Singapore_2003
EU069580.1_Singapore_2003
EU069575.1_Singapore_2003
GQ357784_Singapore_2005
EU069574.1_Singapore_2005
GQ357774_Singapore_2005
KJ806773_Malaysia_2005
EU069585.1_Singapore_2004
JN851127.1_Singapore_2004
MW512341_Singapore_2004

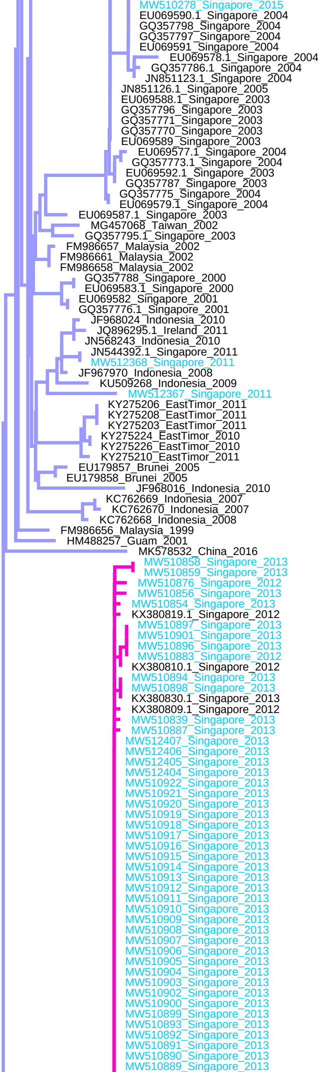
MW510278_Singapore_2015
EU069590.1_Singapore_2004
GQ357798_Singapore_2004
GQ357797_Singapore_2004
EU069591_Singapore_2004
EU069578.1_Singapore_2004
GQ357786.1_Singapore_2004
JN851123.1_Singapore_2004
JN851126.1_Singapore_2005
EU069588.1_Singapore_2003
GQ357796_Singapore_2003
GQ357771_Singapore_2003
GQ357770_Singapore_2003
EU069589_Singapore_2003
EU069577.1_Singapore_2004
GQ357773.1_Singapore_2004
EU069592.1_Singapore_2003
GQ357787_Singapore_2003
GQ357775_Singapore_2004
EU069579.1_Singapore_2004
EU069587.1_Singapore_2003
MG457068_Taiwan_2002
GQ357795.1_Singapore_2003
FM986657_Malaysia_2002
FM986661_Malaysia_2002
FM986658_Malaysia_2002
GQ357788_Singapore_2000
EU069583.1_Singapore_2000
EU069582_Singapore_2001
GQ357776.1_Singapore_2001
JF968024_Indonesia_2010
JQ896295.1_Ireland_2011
JN568243_Indonesia_2010
JN544392.1_Singapore_2011
MW512368_Singapore_2011
JF967970_Indonesia_2008
KU509268_Indonesia_2009
MW512367_Singapore_2011
KY275206_EastTimor_2011
KY275208_EastTimor_2011
KY275203_EastTimor_2011
KY275224_EastTimor_2010
KY275226_EastTimor_2010
KY275210_EastTimor_2011
EU179857_Brunei_2005
EU179858_Brunei_2005
JF968016_Indonesia_2010
KC762669_Indonesia_2007
KC762670_Indonesia_2007
KC762668_Indonesia_2008
FM986656_Malaysia_1999
HM488257_Guam_2001
MK578532_China_2016
MW510858_Singapore_2013
MW510859_Singapore_2013
MW510876_Singapore_2012
MW510856_Singapore_2013
MW510854_Singapore_2013
KX380819.1_Singapore_2012
MW510897_Singapore_2013
MW510901_Singapore_2013
MW510896_Singapore_2013
MW510883_Singapore_2012
KX380810.1_Singapore_2012
MW510894_Singapore_2013
MW510898_Singapore_2013
KX380830.1_Singapore_2013
KX380809.1_Singapore_2012
MW510839_Singapore_2013
MW510887_Singapore_2013
MW512407_Singapore_2013
MW512406_Singapore_2013
MW512405_Singapore_2013
MW512404_Singapore_2013
MW510922_Singapore_2013
MW510921_Singapore_2013
MW510920_Singapore_2013
MW510919_Singapore_2013
MW510918_Singapore_2013
MW510917_Singapore_2013
MW510916_Singapore_2013
MW510915_Singapore_2013
MW510914_Singapore_2013
MW510913_Singapore_2013
MW510912_Singapore_2013
MW510911_Singapore_2013
MW510910_Singapore_2013
MW510909_Singapore_2013
MW510908_Singapore_2013
MW510907_Singapore_2013
MW510906_Singapore_2013
MW510905_Singapore_2013
MW510904_Singapore_2013
MW510903_Singapore_2013
MW510902_Singapore_2013
MW510900_Singapore_2013
MW510899_Singapore_2013
MW510893_Singapore_2013
MW510892_Singapore_2013
MW510891_Singapore_2013
MW510890_Singapore_2013
MW510889_Singapore_2013

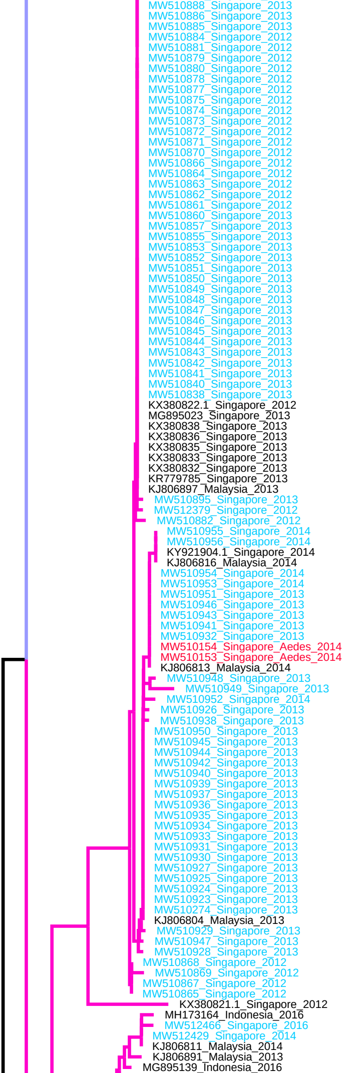
MW510888_Singapore_2013
MW510886_Singapore_2013
MW510885_Singapore_2013
MW510884_Singapore_2012
MW510881_Singapore_2012
MW510879_Singapore_2012
MW510880_Singapore_2012
MW510878_Singapore_2012
MW510877_Singapore_2012
MW510875_Singapore_2012
MW510874_Singapore_2012
MW510873_Singapore_2012
MW510872_Singapore_2012
MW510871_Singapore_2012
MW510870_Singapore_2012
MW510866_Singapore_2012
MW510864_Singapore_2012
MW510863_Singapore_2012
MW510862_Singapore_2012
MW510861_Singapore_2012
MW510860_Singapore_2013
MW510857_Singapore_2013
MW510855_Singapore_2013
MW510853_Singapore_2013
MW510852_Singapore_2013
MW510851_Singapore_2013
MW510850_Singapore_2013
MW510849_Singapore_2013
MW510848_Singapore_2013
MW510847_Singapore_2013
MW510846_Singapore_2013
MW510845_Singapore_2013
MW510844_Singapore_2013
MW510843_Singapore_2013
MW510842_Singapore_2013
MW510841_Singapore_2013
MW510840_Singapore_2013
MW510838_Singapore_2013
KX380822.1_Singapore_2012
MG895023_Singapore_2013
KX380838_Singapore_2013
KX380836_Singapore_2013
KX380835_Singapore_2013
KX380833_Singapore_2013
KX380832_Singapore_2013
KR779785_Singapore_2013
KJ806897_Malaysia_2013
MW510895_Singapore_2013
MW512379_Singapore_2012
MW510882_Singapore_2012
MW510955_Singapore_2014
MW510956_Singapore_2014
KY921904.1_Singapore_2014
KJ806816_Malaysia_2014
MW510954_Singapore_2014
MW510953_Singapore_2014
MW510951_Singapore_2013
MW510946_Singapore_2013
MW510943_Singapore_2013
MW510941_Singapore_2013
MW510932_Singapore_2013
MW510154_Singapore_Aedes_2014
MW510153_Singapore_Aedes_2014
KJ806813_Malaysia_2014
MW510948_Singapore_2013
MW510949_Singapore_2013
MW510952_Singapore_2014
MW510926_Singapore_2013
MW510938_Singapore_2013
MW510950_Singapore_2013
MW510945_Singapore_2013
MW510944_Singapore_2013
MW510942_Singapore_2013
MW510940_Singapore_2013
MW510939_Singapore_2013
MW510937_Singapore_2013
MW510936_Singapore_2013
MW510935_Singapore_2013
MW510934_Singapore_2013
MW510933_Singapore_2013
MW510931_Singapore_2013
MW510930_Singapore_2013
MW510927_Singapore_2013
MW510925_Singapore_2013
MW510924_Singapore_2013
MW510923_Singapore_2013
MW510274_Singapore_2013
KJ806804_Malaysia_2013
MW510929_Singapore_2013
MW510947_Singapore_2013
MW510928_Singapore_2013
MW510868_Singapore_2012
MW510869_Singapore_2012
MW510867_Singapore_2012
MW510865_Singapore_2012
KX380821.1_Singapore_2012
MH173164_Indonesia_2016
MW512466_Singapore_2016
MW512429_Singapore_2014
KJ806811_Malaysia_2014
KJ806891_Malaysia_2013
MG895139_Indonesia_2016

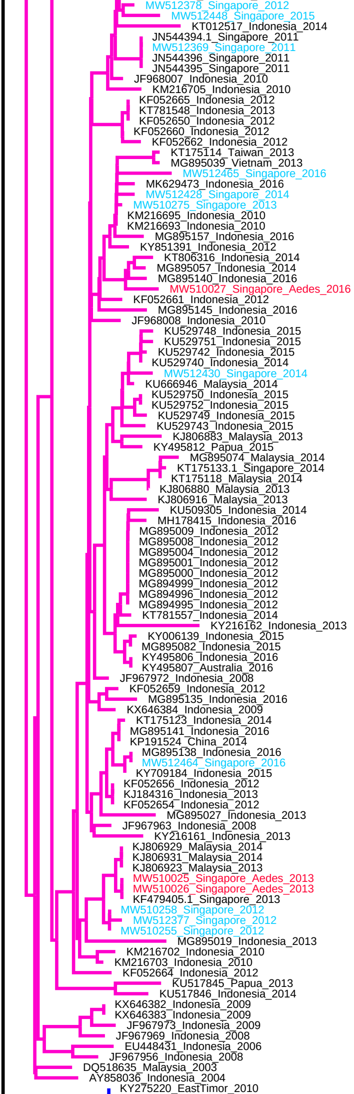
MW512378_Singapore_2012
MW512448_Singapore_2015
KT012517_Indonesia_2014
JN544394.1_Singapore_2011
MW512369_Singapore_2011
JN544396_Singapore_2011
JN544395_Singapore_2011
JF968007_Indonesia_2010
KM216705_Indonesia_2010
KF052665_Indonesia_2012
KT781548_Indonesia_2013
KF052650_Indonesia_2012
KF052660_Indonesia_2012
KF052662_Indonesia_2012
KT175114_Taiwan_2013
MG895039_Vietnam_2013
MW512465_Singapore_2016
MK629473_Indonesia_2016
MW512428_Singapore_2014
MW510275_Singapore_2013
KM216695_Indonesia_2010
KM216693_Indonesia_2010
MG895157_Indonesia_2016
KY851391_Indonesia_2012
KT806316_Indonesia_2014
MG895057_Indonesia_2014
MG895140_Indonesia_2016
MW510027_Singapore_Aedes_2016
KF052661_Indonesia_2012
MG895145_Indonesia_2016
JF968008_Indonesia_2010
KU529748_Indonesia_2015
KU529751_Indonesia_2015
KU529742_Indonesia_2015
KU529740_Indonesia_2014
MW512430_Singapore_2014
KU666946_Malaysia_2014
KU529750_Indonesia_2015
KU529752_Indonesia_2015
KU529749_Indonesia_2015
KU529743_Indonesia_2015
KJ806883_Malaysia_2013
KY495812_Papua_2015
MG895074_Malaysia_2014
KT175133.1_Singapore_2014
KT175118_Malaysia_2014
KJ806880_Malaysia_2013
KJ806916_Malaysia_2013
KU509305_Indonesia_2014
MH178415_Indonesia_2016
MG895009_Indonesia_2012
MG895008_Indonesia_2012
MG895004_Indonesia_2012
MG895001_Indonesia_2012
MG895000_Indonesia_2012
MG894999_Indonesia_2012
MG894996_Indonesia_2012
MG894995_Indonesia_2012
KT781557_Indonesia_2014
KY216162_Indonesia_2013
KY006139_Indonesia_2015
MG895082_Indonesia_2015
KY495806_Indonesia_2016
KY495807_Australia_2016
JF967972_Indonesia_2008
KF052659_Indonesia_2012
MG895135_Indonesia_2016
KX646384_Indonesia_2009
KT175123_Indonesia_2014
MG895141_Indonesia_2016
KP191524_China_2014
MG895138_Indonesia_2016
MW512464_Singapore_2016
KY709184_Indonesia_2015
KF052656_Indonesia_2012
KJ184316_Indonesia_2013
KF052654_Indonesia_2012
MG895027_Indonesia_2013
JF967963_Indonesia_2008
KY216161_Indonesia_2013
KJ806929_Malaysia_2014
KJ806931_Malaysia_2014
KJ806923_Malaysia_2013
MW510025_Singapore_Aedes_2013
MW510026_Singapore_Aedes_2013
KF479405.1_Singapore_2013
MW510258_Singapore_2012
MW512377_Singapore_2012
MW510255_Singapore_2012
MG895019_Indonesia_2013
KM216702_Indonesia_2010
KM216703_Indonesia_2010
KF052664_Indonesia_2012
KU517845_Papua_2013
KU517846_Indonesia_2014
KX646382_Indonesia_2009
KX646383_Indonesia_2009
JF967973_Indonesia_2009
JF967969_Indonesia_2008
EU448431_Indonesia_2006
JF967956_Indonesia_2008
DQ518635_Malaysia_2003
AY858036_Indonesia_2004
KY275220_EastTimor_2010

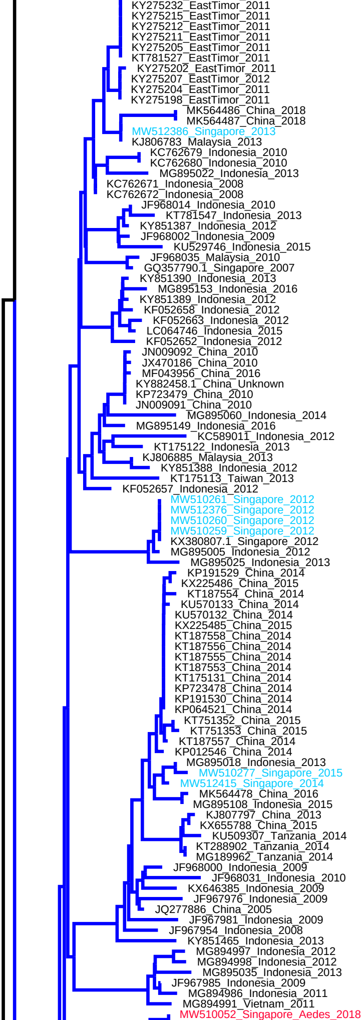
KY275232_EastTimor_2011
KY275215_EastTimor_2011
KY275212_EastTimor_2011
KY275211_EastTimor_2011
KY275205_EastTimor_2011
KT781527_EastTimor_2011
KY275202_EastTimor_2011
KY275207_EastTimor_2012
KY275204_EastTimor_2011
KY275198_EastTimor_2011
MK564486_China_2018
MK564487_China_2018
MW512386_Singapore_2013
KJ806783_Malaysia_2013
KC762679_Indonesia_2010
KC762680_Indonesia_2010
MG895022_Indonesia_2013
KC762671_Indonesia_2008
KC762672_Indonesia_2008
JF968014_Indonesia_2010
KT781547_Indonesia_2013
KY851387_Indonesia_2012
JF968002_Indonesia_2009
KU529746_Indonesia_2015
JF968035_Malaysia_2010
GQ357790.1_Singapore_2007
KY851390_Indonesia_2013
MG895153_Indonesia_2016
KY851389_Indonesia_2012
KF052658_Indonesia_2012
KF052663_Indonesia_2012
LC064746_Indonesia_2015
KF052652_Indonesia_2012
JN009092_China_2010
JX470186_China_2010
MF043956_China_2016
KY882458.1_China_Unknown
KP723479_China_2010
JN009091_China_2010
MG895060_Indonesia_2014
MG895149_Indonesia_2016
KC589011_Indonesia_2012
KT175122_Indonesia_2013
KJ806885_Malaysia_2013
KY851388_Indonesia_2012
KT175113_Taiwan_2013
KF052657_Indonesia_2012
MW510261_Singapore_2012
MW512376_Singapore_2012
MW510260_Singapore_2012
MW510259_Singapore_2012
KX380807.1_Singapore_2012
MG895005_Indonesia_2012
MG895025_Indonesia_2013
KP191529_China_2014
KX225486_China_2015
KT187554_China_2014
KU570133_China_2014
KU570132_China_2014
KX225485_China_2015
KT187558_China_2014
KT187556_China_2014
KT187555_China_2014
KT187553_China_2014
KT175131_China_2014
KP723478_China_2014
KP191530_China_2014
KP064521_China_2014
KT751352_China_2015
KT751353_China_2015
KT187557_China_2014
KP012546_China_2014
MG895018_Indonesia_2013
MW510277_Singapore_2015
MW512415_Singapore_2014
MK564478_China_2016
MG895108_Indonesia_2015
KJ807797_China_2013
KX655788_China_2015
KU509307_Tanzania_2014
KT288902_Tanzania_2014
MG189962_Tanzania_2014
JF968000_Indonesia_2009
JF968031_Indonesia_2010
KX646385_Indonesia_2009
JF967976_Indonesia_2009
JQ277886_China_2005
JF967981_Indonesia_2009
JF967954_Indonesia_2008
KY851465_Indonesia_2013
MG894997_Indonesia_2012
MG894998_Indonesia_2012
MG895035_Indonesia_2013
JF967985_Indonesia_2009
MG894986_Indonesia_2011
MG894991_Vietnam_2011
MW510052_Singapore_Aedes_2018

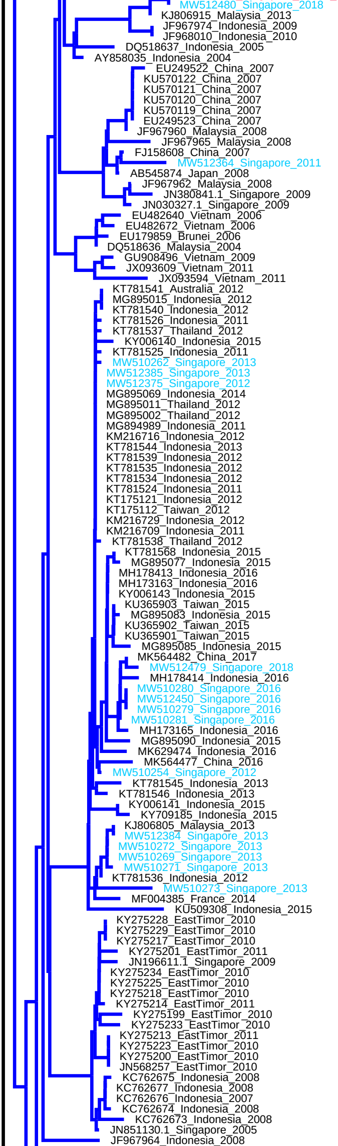

MW512480_Singapore_2018
KJ806915_Malaysia_2013
JF967974_Indonesia_2009
JF968010_Indonesia_2010
DQ518637_Indonesia_2005
AY858035_Indonesia_2004
EU249522_China_2007
KU570122_China_2007
KU570121_China_2007
KU570120_China_2007
KU570119_China_2007
EU249523_China_2007
JF967960_Malaysia_2008
JF967965_Malaysia_2008
FJ158608_China_2007
MW512364_Singapore_2011
AB545874_Japan_2008
JF967962_Malaysia_2008
JN380841.1_Singapore_2009
JN030327.1_Singapore_2009
EU482640_Vietnam_2006
EU482672_Vietnam_2006
EU179859_Brunei_2006
DQ518636_Malaysia_2004
GU908496_Vietnam_2009
JX093609_Vietnam_2011
JX093594_Vietnam_2011
KT781541_Australia_2012
MG895015_Indonesia_2012
KT781540_Indonesia_2012
KT781526_Indonesia_2011
KT781537_Thailand_2012
KY006140_Indonesia_2015
KT781525_Indonesia_2011
MW510262_Singapore_2013
MW512385_Singapore_2013
MW512375_Singapore_2012
MG895069_Indonesia_2014
MG895011_Thailand_2012
MG895002_Thailand_2012
MG894989_Indonesia_2011
KM216716_Indonesia_2012
KT781544_Indonesia_2013
KT781539_Indonesia_2012
KT781535_Indonesia_2012
KT781534_Indonesia_2012
KT781524_Indonesia_2011
KT175121_Indonesia_2012
KT175112_Taiwan_2012
KM216729_Indonesia_2012
KM216709_Indonesia_2011
KT781538_Thailand_2012
KT781568_Indonesia_2015
MG895077_Indonesia_2015
MH178413_Indonesia_2016
MH173163_Indonesia_2016
KY006143_Indonesia_2015
KU365903_Taiwan_2015
MG895083_Indonesia_2015
KU365902_Taiwan_2015
KU365901_Taiwan_2015
MG895085_Indonesia_2015
MK564482_China_2017
MW512479_Singapore_2018
MH178414_Indonesia_2016
MW510280_Singapore_2016
MW512450_Singapore_2016
MW510279_Singapore_2016
MW510281_Singapore_2016
MH173165_Indonesia_2016
MG895090_Indonesia_2015
MK629474_Indonesia_2016
MK564477_China_2016
MW510254_Singapore_2012
KT781545_Indonesia_2013
KT781546_Indonesia_2013
KY006141_Indonesia_2015
KY709185_Indonesia_2015
KJ806805_Malaysia_2013
MW512384_Singapore_2013
MW510272_Singapore_2013
MW510269_Singapore_2013
MW510271_Singapore_2013
KT781536_Indonesia_2012
MW510273_Singapore_2013
MF004385_France_2014
KU509308_Indonesia_2015
KY275228_EastTimor_2010
KY275229_EastTimor_2010
KY275217_EastTimor_2010
KY275201_EastTimor_2011
JN196611.1_Singapore_2009
KY275234_EastTimor_2010
KY275225_EastTimor_2010
KY275218_EastTimor_2010
KY275214_EastTimor_2011
KY275199_EastTimor_2010
KY275233_EastTimor_2010
KY275213_EastTimor_2011
KY275223_EastTimor_2010
KY275200_EastTimor_2010
JN568257_EastTimor_2010
KC762675_Indonesia_2008
KC762677_Indonesia_2008
KC762676_Indonesia_2007
KC762674_Indonesia_2008
KC762673_Indonesia_2008
JN851130.1_Singapore_2005
JF967964_Indonesia_2008

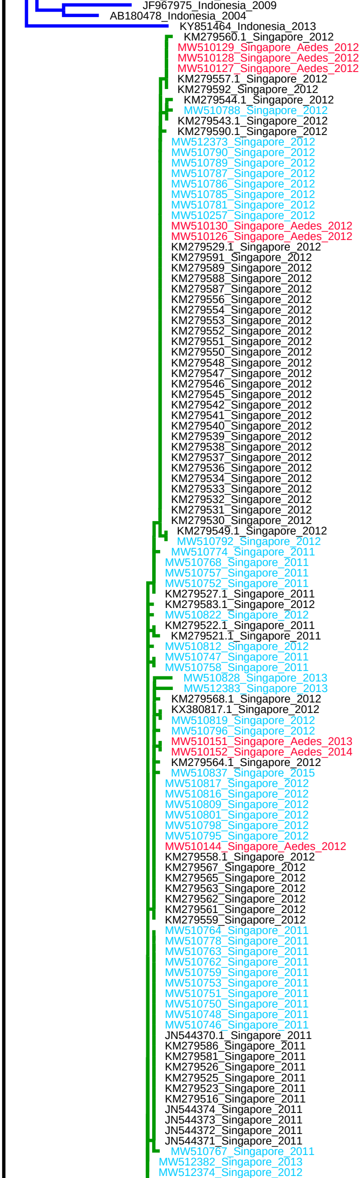
JF967975_Indonesia_2009
AB180478_Indonesia_2004
KY851464_Indonesia_2013
KM279560.1_Singapore_2012
MW510129_Singapore_Aedes_2012
MW510128_Singapore_Aedes_2012
MW510127_Singapore_Aedes_2012
KM279557.1_Singapore_2012
KM279592_Singapore_2012
KM279544.1_Singapore_2012
MW510788_Singapore_2012
KM279543.1_Singapore_2012
KM279590.1_Singapore_2012
MW512373_Singapore_2012
MW510790_Singapore_2012
MW510789_Singapore_2012
MW510787_Singapore_2012
MW510786_Singapore_2012
MW510785_Singapore_2012
MW510781_Singapore_2012
MW510257_Singapore_2012
MW510130_Singapore_Aedes_2012
MW510126_Singapore_Aedes_2012
KM279529.1_Singapore_2012
KM279591_Singapore_2012
KM279589_Singapore_2012
KM279588_Singapore_2012
KM279587_Singapore_2012
KM279556_Singapore_2012
KM279554_Singapore_2012
KM279553_Singapore_2012
KM279552_Singapore_2012
KM279551_Singapore_2012
KM279550_Singapore_2012
KM279548_Singapore_2012
KM279547_Singapore_2012
KM279546_Singapore_2012
KM279545_Singapore_2012
KM279542_Singapore_2012
KM279541_Singapore_2012
KM279540_Singapore_2012
KM279539_Singapore_2012
KM279538_Singapore_2012
KM279537_Singapore_2012
KM279536_Singapore_2012
KM279534_Singapore_2012
KM279533_Singapore_2012
KM279532_Singapore_2012
KM279531_Singapore_2012
KM279530_Singapore_2012
KM279549.1_Singapore_2012
MW510792_Singapore_2012
MW510774_Singapore_2011
MW510768_Singapore_2011
MW510757_Singapore_2011
MW510752_Singapore_2011
KM279527.1_Singapore_2011
KM279583.1_Singapore_2012
MW510822_Singapore_2012
KM279522.1_Singapore_2011
KM279521.1_Singapore_2011
MW510812_Singapore_2012
MW510747_Singapore_2011
MW510758_Singapore_2011
MW510828_Singapore_2013
MW512383_Singapore_2013
KM279568.1_Singapore_2012
KX380817.1_Singapore_2012
MW510819_Singapore_2012
MW510796_Singapore_2012
MW510151_Singapore_Aedes_2013
MW510152_Singapore_Aedes_2014
KM279564.1_Singapore_2012
MW510837_Singapore_2015
MW510817_Singapore_2012
MW510816_Singapore_2012
MW510809_Singapore_2012
MW510801_Singapore_2012
MW510798_Singapore_2012
MW510795_Singapore_2012
MW510144_Singapore_Aedes_2012
KM279558.1_Singapore_2012
KM279567_Singapore_2012
KM279565_Singapore_2012
KM279563_Singapore_2012
KM279562_Singapore_2012
KM279561_Singapore_2012
KM279559_Singapore_2012
MW510764_Singapore_2011
MW510778_Singapore_2011
MW510763_Singapore_2011
MW510762_Singapore_2011
MW510759_Singapore_2011
MW510753_Singapore_2011
MW510751_Singapore_2011
MW510750_Singapore_2011
MW510748_Singapore_2011
MW510746_Singapore_2011
JN544370.1_Singapore_2011
KM279586_Singapore_2011
KM279581_Singapore_2011
KM279526_Singapore_2011
KM279525_Singapore_2011
KM279523_Singapore_2011
KM279516_Singapore_2011
JN544374_Singapore_2011
JN544373_Singapore_2011
JN544372_Singapore_2011
JN544371_Singapore_2011
MW510767_Singapore_2011
MW512382_Singapore_2013
MW512374_Singapore_2012

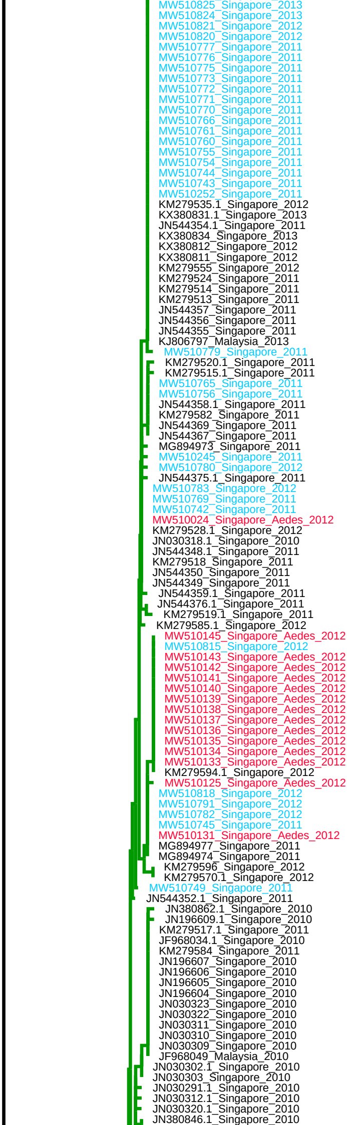
MW510825_Singapore_2013
MW510824_Singapore_2013
MW510821_Singapore_2012
MW510820_Singapore_2012
MW510777_Singapore_2011
MW510776_Singapore_2011
MW510775_Singapore_2011
MW510773_Singapore_2011
MW510772_Singapore_2011
MW510771_Singapore_2011
MW510770_Singapore_2011
MW510766_Singapore_2011
MW510761_Singapore_2011
MW510760_Singapore_2011
MW510755_Singapore_2011
MW510754_Singapore_2011
MW510744_Singapore_2011
MW510743_Singapore_2011
MW510252_Singapore_2011
KM279535.1_Singapore_2012
KX380831.1_Singapore_2013
JN544354.1_Singapore_2011
KX380834_Singapore_2013
KX380812_Singapore_2012
KX380811_Singapore_2012
KM279555_Singapore_2012
KM279524_Singapore_2011
KM279514_Singapore_2011
KM279513_Singapore_2011
JN544357_Singapore_2011
JN544356_Singapore_2011
JN544355_Singapore_2011
KJ806797_Malaysia_2013
MW510779_Singapore_2011
KM279520.1_Singapore_2011
KM279515.1_Singapore_2011
MW510765_Singapore_2011
MW510756_Singapore_2011
JN544358.1_Singapore_2011
KM279582_Singapore_2011
JN544369_Singapore_2011
JN544367_Singapore_2011
MG894973_Singapore_2011
MW510245_Singapore_2011
MW510780_Singapore_2012
JN544375.1_Singapore_2011
MW510783_Singapore_2012
MW510769_Singapore_2011
MW510742_Singapore_2011
MW510024_Singapore_Aedes_2012
KM279528.1_Singapore_2012
JN030318.1_Singapore_2010
JN544348.1_Singapore_2011
KM279518_Singapore_2011
JN544350_Singapore_2011
JN544349_Singapore_2011
JN544359.1_Singapore_2011
JN544376.1_Singapore_2011
KM279519.1_Singapore_2011
KM279585.1_Singapore_2012
MW510145_Singapore_Aedes_2012
MW510815_Singapore_2012
MW510143_Singapore_Aedes_2012
MW510142_Singapore_Aedes_2012
MW510141_Singapore_Aedes_2012
MW510140_Singapore_Aedes_2012
MW510139_Singapore_Aedes_2012
MW510138_Singapore_Aedes_2012
MW510137_Singapore_Aedes_2012
MW510136_Singapore_Aedes_2012
MW510135_Singapore_Aedes_2012
MW510134_Singapore_Aedes_2012
MW510133_Singapore_Aedes_2012
KM279594.1_Singapore_2012
MW510125_Singapore_Aedes_2012
MW510818_Singapore_2012
MW510791_Singapore_2012
MW510782_Singapore_2012
MW510745_Singapore_2011
MW510131_Singapore_Aedes_2012
MG894977_Singapore_2011
MG894974_Singapore_2011
KM279596_Singapore_2012
KM279570.1_Singapore_2012
MW510749_Singapore_2011
JN544352.1_Singapore_2011
JN380862.1_Singapore_2010
JN196609.1_Singapore_2010
KM279517.1_Singapore_2011
JF968034.1_Singapore_2010
KM279584_Singapore_2011
JN196607_Singapore_2010
JN196606_Singapore_2010
JN196605_Singapore_2010
JN196604_Singapore_2010
JN030323_Singapore_2010
JN030322_Singapore_2010
JN030311_Singapore_2010
JN030310_Singapore_2010
JN030309_Singapore_2010
JF968049_Malaysia_2010
JN030302.1_Singapore_2010
JN030303_Singapore_2010
JN030291.1_Singapore_2010
JN030312.1_Singapore_2010
JN030320.1_Singapore_2010
JN380846.1_Singapore_2010

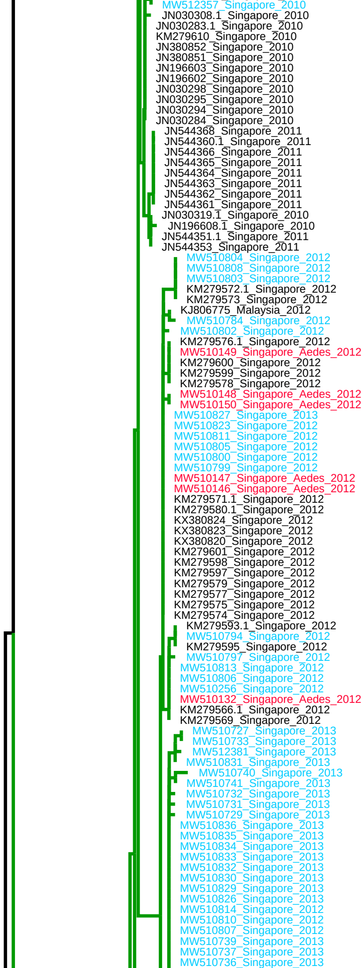
MW512357_Singapore_2010
JN030308.1_Singapore_2010
JN030283.1_Singapore_2010
KM279610_Singapore_2010
JN380852_Singapore_2010
JN380851_Singapore_2010
JN196603_Singapore_2010
JN196602_Singapore_2010
JN030298_Singapore_2010
JN030295_Singapore_2010
JN030294_Singapore_2010
JN030284_Singapore_2010
JN544368_Singapore_2011
JN544360.1_Singapore_2011
JN544366_Singapore_2011
JN544365_Singapore_2011
JN544364_Singapore_2011
JN544363_Singapore_2011
JN544362_Singapore_2011
JN544361_Singapore_2011
JN030319.1_Singapore_2010
JN196608.1_Singapore_2010
JN544351.1_Singapore_2011
JN544353_Singapore_2011
MW510804_Singapore_2012
MW510808_Singapore_2012
MW510803_Singapore_2012
KM279572.1_Singapore_2012
KM279573_Singapore_2012
KJ806775_Malaysia_2012
MW510784_Singapore_2012
MW510802_Singapore_2012
KM279576.1_Singapore_2012
MW510149_Singapore_Aedes_2012
KM279600_Singapore_2012
KM279599_Singapore_2012
KM279578_Singapore_2012
MW510148_Singapore_Aedes_2012
MW510150_Singapore_Aedes_2012
MW510827_Singapore_2013
MW510823_Singapore_2012
MW510811_Singapore_2012
MW510805_Singapore_2012
MW510800_Singapore_2012
MW510799_Singapore_2012
MW510147_Singapore_Aedes_2012
MW510146_Singapore_Aedes_2012
KM279571.1_Singapore_2012
KM279580.1_Singapore_2012
KX380824_Singapore_2012
KX380823_Singapore_2012
KX380820_Singapore_2012
KM279601_Singapore_2012
KM279598_Singapore_2012
KM279597_Singapore_2012
KM279579_Singapore_2012
KM279577_Singapore_2012
KM279575_Singapore_2012
KM279574_Singapore_2012
KM279593.1_Singapore_2012
MW510794_Singapore_2012
KM279595_Singapore_2012
MW510797_Singapore_2012
MW510813_Singapore_2012
MW510806_Singapore_2012
MW510256_Singapore_2012
MW510132_Singapore_Aedes_2012
KM279566.1_Singapore_2012
KM279569_Singapore_2012
MW510727_Singapore_2013
MW510733_Singapore_2013
MW512381_Singapore_2013
MW510831_Singapore_2013
MW510740_Singapore_2013
MW510741_Singapore_2013
MW510732_Singapore_2013
MW510731_Singapore_2013
MW510729_Singapore_2013
MW510836_Singapore_2013
MW510835_Singapore_2013
MW510834_Singapore_2013
MW510833_Singapore_2013
MW510832_Singapore_2013
MW510830_Singapore_2013
MW510829_Singapore_2013
MW510826_Singapore_2013
MW510814_Singapore_2012
MW510810_Singapore_2012
MW510807_Singapore_2012
MW510739_Singapore_2013
MW510737_Singapore_2013
MW510736_Singapore_2013

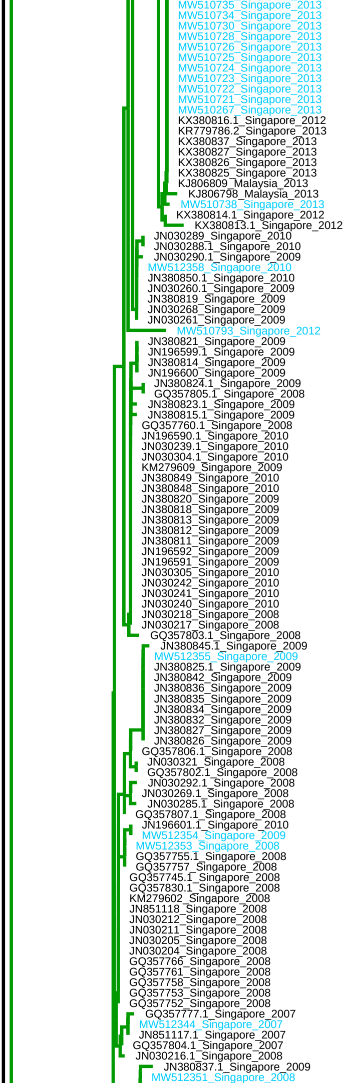
MW510735_Singapore_2013
MW510734_Singapore_2013
MW510730_Singapore_2013
MW510728_Singapore_2013
MW510726_Singapore_2013
MW510725_Singapore_2013
MW510724_Singapore_2013
MW510723_Singapore_2013
MW510722_Singapore_2013
MW510721_Singapore_2013
MW510267_Singapore_2013
KX380816.1_Singapore_2012
KR779786.2_Singapore_2013
KX380837_Singapore_2013
KX380827_Singapore_2013
KX380826_Singapore_2013
KX380825_Singapore_2013
KJ806809_Malaysia_2013
KJ806798_Malaysia_2013
MW510738_Singapore_2013
KX380814.1_Singapore_2012
KX380813.1_Singapore_2012
JN030289_Singapore_2010
JN030288.1_Singapore_2010
JN030290.1_Singapore_2009
MW512358_Singapore_2010
JN380850.1_Singapore_2010
JN030260.1_Singapore_2009
JN380819_Singapore_2009
JN030268_Singapore_2009
JN030261_Singapore_2009
MW510793_Singapore_2012
JN380821_Singapore_2009
JN196599.1_Singapore_2009
JN380814_Singapore_2009
JN196600_Singapore_2009
JN380824.1_Singapore_2009
GQ357805.1_Singapore_2008
JN380823.1_Singapore_2009
JN380815.1_Singapore_2009
GQ357760.1_Singapore_2008
JN196590.1_Singapore_2010
JN030239.1_Singapore_2010
JN030304.1_Singapore_2010
KM279609_Singapore_2009
JN380849_Singapore_2010
JN380848_Singapore_2010
JN380820_Singapore_2009
JN380818_Singapore_2009
JN380813_Singapore_2009
JN380812_Singapore_2009
JN380811_Singapore_2009
JN196592_Singapore_2009
JN196591_Singapore_2009
JN030305_Singapore_2010
JN030242_Singapore_2010
JN030241_Singapore_2010
JN030240_Singapore_2010
JN030218_Singapore_2008
JN030217_Singapore_2008
GQ357803.1_Singapore_2008
JN380845.1_Singapore_2009
MW512355_Singapore_2009
JN380825.1_Singapore_2009
JN380842_Singapore_2009
JN380836_Singapore_2009
JN380835_Singapore_2009
JN380834_Singapore_2009
JN380832_Singapore_2009
JN380827_Singapore_2009
JN380826_Singapore_2009
GQ357806.1_Singapore_2008
JN030321_Singapore_2008
GQ357802.1_Singapore_2008
JN030292.1_Singapore_2008
JN030269.1_Singapore_2008
JN030285.1_Singapore_2008
GQ357807.1_Singapore_2008
JN196601.1_Singapore_2010
MW512354_Singapore_2009
MW512353_Singapore_2008
GQ357755.1_Singapore_2008
GQ357757_Singapore_2008
GQ357745.1_Singapore_2008
GQ357830.1_Singapore_2008
KM279602_Singapore_2008
JN851118_Singapore_2008
JN030212_Singapore_2008
JN030211_Singapore_2008
JN030205_Singapore_2008
JN030204_Singapore_2008
GQ357766_Singapore_2008
GQ357761_Singapore_2008
GQ357758_Singapore_2008
GQ357753_Singapore_2008
GQ357752_Singapore_2008
GQ357777.1_Singapore_2007
MW512344_Singapore_2007
JN851117.1_Singapore_2007
GQ357804.1_Singapore_2007
JN030216.1_Singapore_2008
JN380837.1_Singapore_2009
MW512351_Singapore_2008

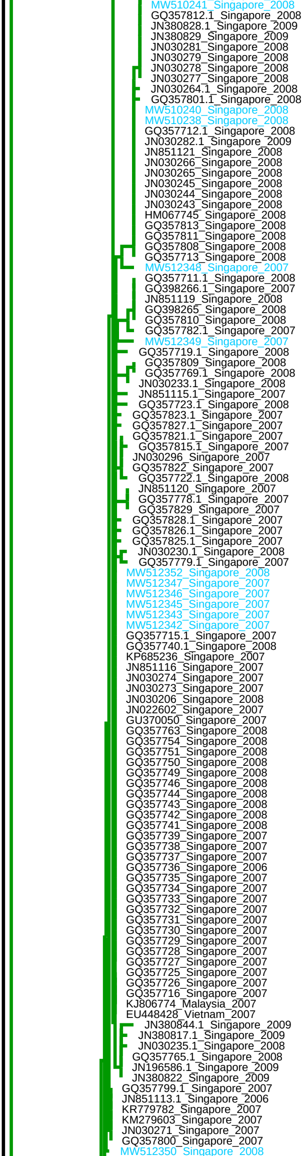
MW510241_Singapore_2008
GQ357812.1_Singapore_2008
JN380828.1_Singapore_2009
JN380829_Singapore_2009
JN030281_Singapore_2008
JN030279_Singapore_2008
JN030278_Singapore_2008
JN030277_Singapore_2008
JN030264.1_Singapore_2008
GQ357801.1_Singapore_2008
MW510240_Singapore_2008
MW510238_Singapore_2008
GQ357712.1_Singapore_2008
JN030282.1_Singapore_2009
JN851121_Singapore_2008
JN030266_Singapore_2008
JN030265_Singapore_2008
JN030245_Singapore_2008
JN030244_Singapore_2008
JN030243_Singapore_2008
HM067745_Singapore_2008
GQ357813_Singapore_2008
GQ357811_Singapore_2008
GQ357808_Singapore_2008
GQ357713_Singapore_2008
MW512348_Singapore_2007
GQ357711.1_Singapore_2008
GQ398266.1_Singapore_2007
JN851119_Singapore_2008
GQ398265_Singapore_2008
GQ357810_Singapore_2008
GQ357782.1_Singapore_2007
MW512349_Singapore_2007
GQ357719.1_Singapore_2008
GQ357809_Singapore_2008
GQ357769.1_Singapore_2008
JN030233.1_Singapore_2008
JN851115.1_Singapore_2007
GQ357723.1_Singapore_2008
GQ357823.1_Singapore_2007
GQ357827.1_Singapore_2007
GQ357821.1_Singapore_2007
GQ357815.1_Singapore_2007
JN030296_Singapore_2007
GQ357822_Singapore_2007
GQ357722.1_Singapore_2008
JN851120_Singapore_2007
GQ357778.1_Singapore_2007
GQ357829_Singapore_2007
GQ357828.1_Singapore_2007
GQ357826.1_Singapore_2007
GQ357825.1_Singapore_2007
JN030230.1_Singapore_2008
GQ357779.1_Singapore_2007
MW512352_Singapore_2008
MW512347_Singapore_2007
MW512346_Singapore_2007
MW512345_Singapore_2007
MW512343_Singapore_2007
MW512342_Singapore_2007
GQ357715.1_Singapore_2007
GQ357740.1_Singapore_2008
KP685236_Singapore_2007
JN851116_Singapore_2007
JN030274_Singapore_2007
JN030273_Singapore_2007
JN030206_Singapore_2008
JN022602_Singapore_2007
GU370050_Singapore_2007
GQ357763_Singapore_2008
GQ357754_Singapore_2008
GQ357751_Singapore_2008
GQ357750_Singapore_2008
GQ357749_Singapore_2008
GQ357746_Singapore_2008
GQ357744_Singapore_2008
GQ357743_Singapore_2008
GQ357742_Singapore_2008
GQ357741_Singapore_2008
GQ357739_Singapore_2007
GQ357738_Singapore_2007
GQ357737_Singapore_2007
GQ357736_Singapore_2006
GQ357735_Singapore_2007
GQ357734_Singapore_2007
GQ357733_Singapore_2007
GQ357732_Singapore_2007
GQ357731_Singapore_2007
GQ357730_Singapore_2007
GQ357729_Singapore_2007
GQ357728_Singapore_2007
GQ357727_Singapore_2007
GQ357725_Singapore_2007
GQ357726_Singapore_2007
GQ357716_Singapore_2007
KJ806774_Malaysia_2007
EU448428_Vietnam_2007
JN380844.1_Singapore_2009
JN380817.1_Singapore_2009
JN030235.1_Singapore_2008
GQ357765.1_Singapore_2008
JN196586.1_Singapore_2009
JN380822_Singapore_2009
GQ357799.1_Singapore_2007
JN851113.1_Singapore_2006
KR779782_Singapore_2007
KM279603_Singapore_2007
JN030271_Singapore_2007
GQ357800_Singapore_2007
MW512350_Singapore_2008

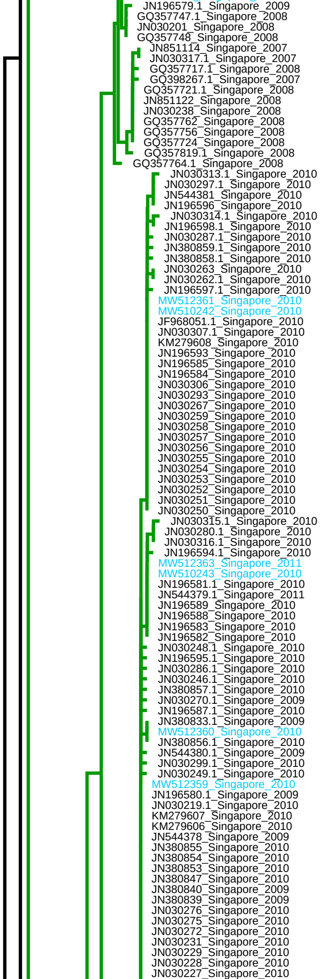
JN196579.1_Singapore_2009
GQ357747.1_Singapore_2008
JN030201_Singapore_2008
GQ357748_Singapore_2008
JN851114_Singapore_2007
JN030317.1_Singapore_2007
GQ357717.1_Singapore_2008
GQ398267.1_Singapore_2007
GQ357721.1_Singapore_2008
JN851122_Singapore_2008
JN030238_Singapore_2008
GQ357762_Singapore_2008
GQ357756_Singapore_2008
GQ357724_Singapore_2008
GQ357819.1_Singapore_2008
GQ357764.1_Singapore_2008
JN030313.1_Singapore_2010
JN030297.1_Singapore_2010
JN544381_Singapore_2010
JN196596_Singapore_2010
JN030314.1_Singapore_2010
JN196598.1_Singapore_2010
JN030287.1_Singapore_2010
JN380859.1_Singapore_2010
JN380858.1_Singapore_2010
JN030263_Singapore_2010
JN030262.1_Singapore_2010
JN196597.1_Singapore_2010
MW512361_Singapore_2010
MW510242_Singapore_2010
JF968051.1_Singapore_2010
JN030307.1_Singapore_2010
KM279608_Singapore_2010
JN196593_Singapore_2010
JN196585_Singapore_2010
JN196584_Singapore_2010
JN030306_Singapore_2010
JN030293_Singapore_2010
JN030267_Singapore_2010
JN030259_Singapore_2010
JN030258_Singapore_2010
JN030257_Singapore_2010
JN030256_Singapore_2010
JN030255_Singapore_2010
JN030254_Singapore_2010
JN030253_Singapore_2010
JN030252_Singapore_2010
JN030251_Singapore_2010
JN030250_Singapore_2010
JN030315.1_Singapore_2010
JN030280.1_Singapore_2010
JN030316.1_Singapore_2010
JN196594.1_Singapore_2010
MW512363_Singapore_2011
MW510243_Singapore_2010
JN196581.1_Singapore_2010
JN544379.1_Singapore_2011
JN196589_Singapore_2010
JN196588_Singapore_2010
JN196583_Singapore_2010
JN196582_Singapore_2010
JN030248.1_Singapore_2010
JN196595.1_Singapore_2010
JN030286.1_Singapore_2010
JN030246.1_Singapore_2010
JN380857.1_Singapore_2010
JN030270.1_Singapore_2009
JN196587.1_Singapore_2010
JN380833.1_Singapore_2009
MW512360_Singapore_2010
JN380856.1_Singapore_2010
JN544380.1_Singapore_2009
JN030299.1_Singapore_2010
JN030249.1_Singapore_2010
MW512359_Singapore_2010
JN196580.1_Singapore_2009
JN030219.1_Singapore_2010
KM279607_Singapore_2010
KM279606_Singapore_2010
JN544378_Singapore_2009
JN380855_Singapore_2010
JN380854_Singapore_2010
JN380853_Singapore_2010
JN380847_Singapore_2010
JN380840_Singapore_2009
JN380839_Singapore_2009
JN030276_Singapore_2010
JN030275_Singapore_2010
JN030272_Singapore_2010
JN030231_Singapore_2010
JN030229_Singapore_2010
JN030228_Singapore_2010
JN030227_Singapore_2010

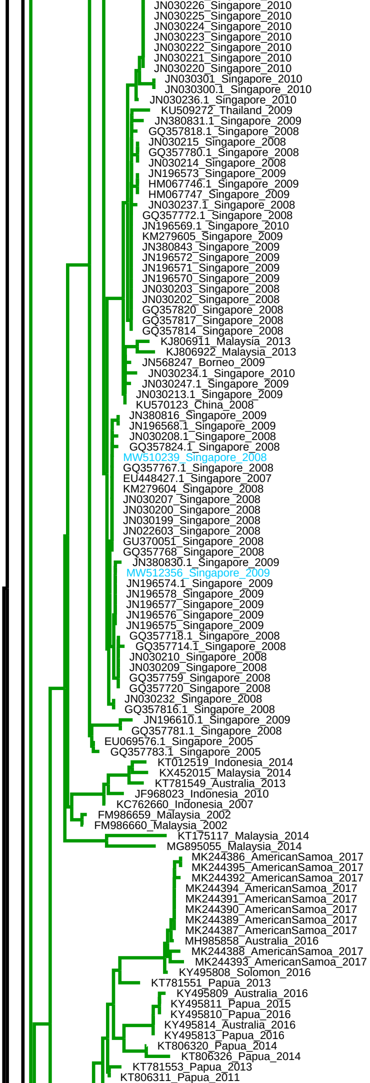

JN030226_Singapore_2010
JN030225_Singapore_2010
JN030224_Singapore_2010
JN030223_Singapore_2010
JN030222_Singapore_2010
JN030221_Singapore_2010
JN030220_Singapore_2010
JN030301_Singapore_2010
JN030300.1_Singapore_2010
JN030236.1_Singapore_2010
KU509272_Thailand_2009
JN380831.1_Singapore_2009
GQ357818.1_Singapore_2008
JN030215_Singapore_2008
GQ357780.1_Singapore_2008
JN030214_Singapore_2008
JN196573_Singapore_2009
HM067746.1_Singapore_2009
HM067747_Singapore_2009
JN030237.1_Singapore_2008
GQ357772.1_Singapore_2008
JN196569.1_Singapore_2010
KM279605_Singapore_2009
JN380843_Singapore_2009
JN196572_Singapore_2009
JN196571_Singapore_2009
JN196570_Singapore_2009
JN030203_Singapore_2008
JN030202_Singapore_2008
GQ357820_Singapore_2008
GQ357817_Singapore_2008
GQ357814_Singapore_2008
KJ806911_Malaysia_2013
KJ806922_Malaysia_2013
JN568247_Borneo_2009
JN030234.1_Singapore_2010
JN030247.1_Singapore_2009
JN030213.1_Singapore_2009
KU570123_China_2008
JN380816_Singapore_2009
JN196568.1_Singapore_2009
JN030208.1_Singapore_2008
GQ357824.1_Singapore_2008
MW510239_Singapore_2008
GQ357767.1_Singapore_2008
EU448427.1_Singapore_2007
KM279604_Singapore_2008
JN030207_Singapore_2008
JN030200_Singapore_2008
JN030199_Singapore_2008
JN022603_Singapore_2008
GU370051_Singapore_2008
GQ357768_Singapore_2008
JN380830.1_Singapore_2009
MW512356_Singapore_2009
JN196574.1_Singapore_2009
JN196578_Singapore_2009
JN196577_Singapore_2009
JN196576_Singapore_2009
JN196575_Singapore_2009
GQ357718.1_Singapore_2008
GQ357714.1_Singapore_2008
JN030210_Singapore_2008
JN030209_Singapore_2008
GQ357759_Singapore_2008
GQ357720_Singapore_2008
JN030232_Singapore_2008
GQ357816.1_Singapore_2008
JN196610.1_Singapore_2009
GQ357781.1_Singapore_2008
EU069576.1_Singapore_2005
GQ357783.1_Singapore_2005
KT012519_Indonesia_2014
KX452015_Malaysia_2014
KT781549_Australia_2013
JF968023_Indonesia_2010
KC762660_Indonesia_2007
FM986659_Malaysia_2002
FM986660_Malaysia_2002
KT175117_Malaysia_2014
MG895055_Malaysia_2014
MK244386_AmericanSamoa_2017
MK244395_AmericanSamoa_2017
MK244392_AmericanSamoa_2017
MK244394_AmericanSamoa_2017
MK244391_AmericanSamoa_2017
MK244390_AmericanSamoa_2017
MK244389_AmericanSamoa_2017
MK244387_AmericanSamoa_2017
MH985858_Australia_2016
MK244388_AmericanSamoa_2017
MK244393_AmericanSamoa_2017
KY495808_Solomon_2016
KT781551_Papua_2013
KY495809_Australia_2016
KY495811_Papua_2015
KY495810_Papua_2016
KY495814_Australia_2016
KY495813_Papua_2016
KT806320_Papua_2014
KT806326_Papua_2014
KT781553_Papua_2013
KT806311_Papua_2011

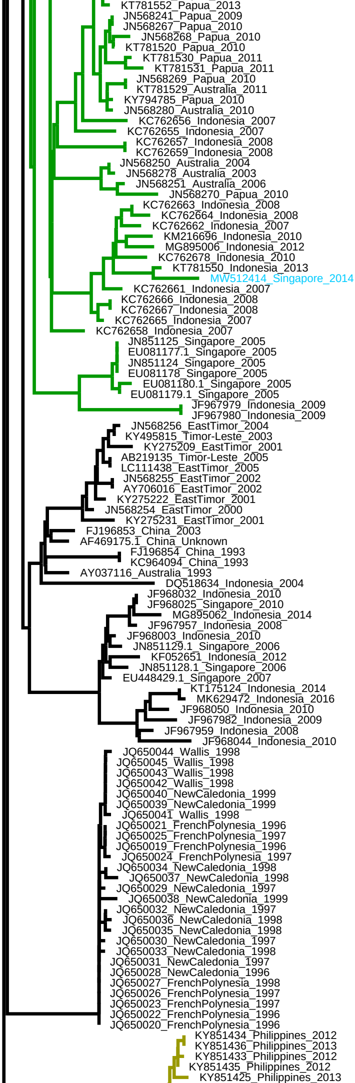
KT781552_Papua_2013
JN568241_Papua_2009
JN568267_Papua_2010
JN568268_Papua_2010
KT781520_Papua_2010
KT781530_Papua_2011
KT781531_Papua_2011
JN568269_Papua_2010
KT781529_Australia_2011
KY794785_Papua_2010
JN568280_Australia_2010
KC762656_Indonesia_2007
KC762655_Indonesia_2007
KC762657_Indonesia_2008
KC762659_Indonesia_2008
JN568250_Australia_2004
JN568278_Australia_2003
JN568251_Australia_2006
JN568270_Papua_2010
KC762663_Indonesia_2008
KC762664_Indonesia_2008
KC762662_Indonesia_2007
KM216696_Indonesia_2010
MG895006_Indonesia_2012
KC762678_Indonesia_2010
KT781550_Indonesia_2013
MW512414_Singapore_2014
KC762661_Indonesia_2007
KC762666_Indonesia_2008
KC762667_Indonesia_2008
KC762665_Indonesia_2007
KC762658_Indonesia_2007
JN851125_Singapore_2005
EU081177.1_Singapore_2005
JN851124_Singapore_2005
EU081178_Singapore_2005
EU081180.1_Singapore_2005
EU081179.1_Singapore_2005
JF967979_Indonesia_2009
JF967980_Indonesia_2009
JN568256_EastTimor_2004
KY495815_Timor-Leste_2003
KY275209_EastTimor_2001
AB219135_Timor-Leste_2005
LC111438_EastTimor_2005
JN568255_EastTimor_2002
AY706016_EastTimor_2002
KY275222_EastTimor_2001
JN568254_EastTimor_2000
KY275231_EastTimor_2001
FJ196853_China_2003
AF469175.1_China_Unknown
FJ196854_China_1993
KC964094_China_1993
AY037116_Australia_1993
DQ518634_Indonesia_2004
JF968032_Indonesia_2010
JF968025_Singapore_2010
MG895062_Indonesia_2014
JF967957_Indonesia_2008
JF968003_Indonesia_2010
JN851129.1_Singapore_2006
KF052651_Indonesia_2012
JN851128.1_Singapore_2006
EU448429.1_Singapore_2007
KT175124_Indonesia_2014
MK629472_Indonesia_2016
JF968050_Indonesia_2010
JF967982_Indonesia_2009
JF967959_Indonesia_2008
JF968044_Indonesia_2010
JQ650044_Wallis_1998
JQ650045_Wallis_1998
JQ650043_Wallis_1998
JQ650042_Wallis_1998
JQ650040_NewCaledonia_1999
JQ650039_NewCaledonia_1999
JQ650041_Wallis_1998
JQ650021_FrenchPolynesia_1996
JQ650025_FrenchPolynesia_1997
JQ650019_FrenchPolynesia_1996
JQ650024_FrenchPolynesia_1997
JQ650034_NewCaledonia_1998
JQ650037_NewCaledonia_1998
JQ650029_NewCaledonia_1997
JQ650038_NewCaledonia_1999
JQ650032_NewCaledonia_1997
JQ650036_NewCaledonia_1998
JQ650035_NewCaledonia_1998
JQ650030_NewCaledonia_1997
JQ650033_NewCaledonia_1998
JQ650031_NewCaledonia_1997
JQ650028_NewCaledonia_1996
JQ650027_FrenchPolynesia_1998
JQ650026_FrenchPolynesia_1997
JQ650023_FrenchPolynesia_1997
JQ650022_FrenchPolynesia_1996
JQ650020_FrenchPolynesia_1996
KY851434_Philippines_2012
KY851436_Philippines_2013
KY851433_Philippines_2012
KY851435_Philippines_2012
KY851425_Philippines_2013

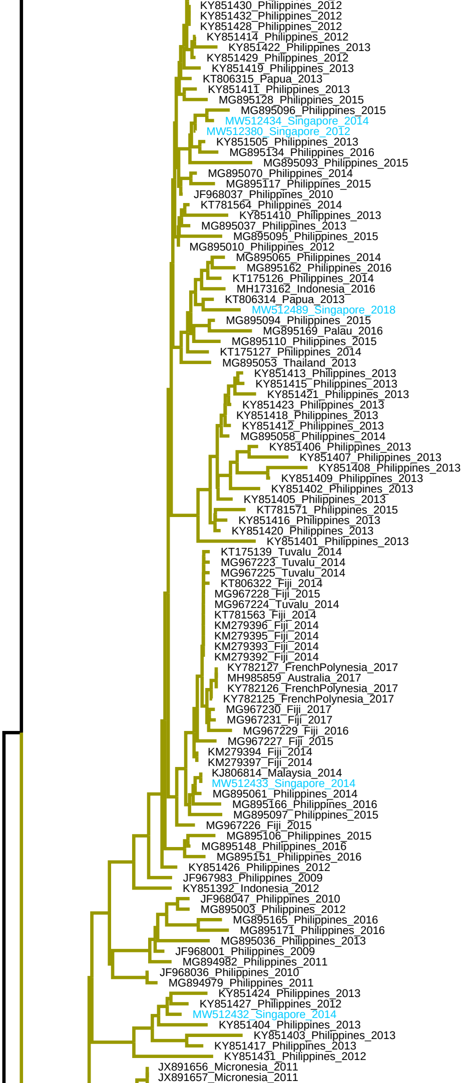

KY851430_Philippines_2012
KY851432_Philippines_2012
KY851428_Philippines_2012
KY851414_Philippines_2012
KY851422_Philippines_2013
KY851429_Philippines_2012
KY851419_Philippines_2013
KT806315_Papua_2013
KY851411_Philippines_2013
MG895128_Philippines_2015
MG895096_Philippines_2015
MW512434_Singapore_2014
MW512380_Singapore_2012
KY851505_Philippines_2013
MG895134_Philippines_2016
MG895093_Philippines_2015
MG895070_Philippines_2014
MG895117_Philippines_2015
JF968037_Philippines_2010
KT781564_Philippines_2014
KY851410_Philippines_2013
MG895037_Philippines_2013
MG895095_Philippines_2015
MG895010_Philippines_2012
MG895065_Philippines_2014
MG895162_Philippines_2016
KT175126_Philippines_2014
MH173162_Indonesia_2016
KT806314_Papua_2013
MW512489_Singapore_2018
MG895094_Philippines_2015
MG895169_Palau_2016
MG895110_Philippines_2015
KT175127_Philippines_2014
MG895053_Thailand_2013
KY851413_Philippines_2013
KY851415_Philippines_2013
KY851421_Philippines_2013
KY851423_Philippines_2013
KY851418_Philippines_2013
KY851412_Philippines_2013
MG895058_Philippines_2014
KY851406_Philippines_2013
KY851407_Philippines_2013
KY851408_Philippines_2013
KY851409_Philippines_2013
KY851402_Philippines_2013
KY851405_Philippines_2013
KT781571_Philippines_2015
KY851416_Philippines_2013
KY851420_Philippines_2013
KY851401_Philippines_2013
KT175139_Tuvalu_2014
MG967223_Tuvalu_2014
MG967225_Tuvalu_2014
KT806322_Fiji_2014
MG967228_Fiji_2015
MG967224_Tuvalu_2014
KT781563_Fiji_2014
KM279396_Fiji_2014
KM279395_Fiji_2014
KM279393_Fiji_2014
KM279392_Fiji_2014
KY782127_FrenchPolynesia_2017
MH985859_Australia_2017
KY782126_FrenchPolynesia_2017
KY782125_FrenchPolynesia_2017
MG967230_Fiji_2017
MG967231_Fiji_2017
MG967229_Fiji_2016
MG967227_Fiji_2015
KM279394_Fiji_2014
KM279397_Fiji_2014
KJ806814_Malaysia_2014
MW512433_Singapore_2014
MG895061_Philippines_2014
MG895166_Philippines_2016
MG895097_Philippines_2015
MG967226_Fiji_2015
MG895106_Philippines_2015
MG895148_Philippines_2016
MG895151_Philippines_2016
KY851426_Philippines_2012
JF967983_Philippines_2009
KY851392_Indonesia_2012
JF968047_Philippines_2010
MG895003_Philippines_2012
MG895165_Philippines_2016
MG895171_Philippines_2016
MG895036_Philippines_2013
JF968001_Philippines_2009
MG894982_Philippines_2011
JF968036_Philippines_2010
MG894979_Philippines_2011
KY851424_Philippines_2013
KY851427_Philippines_2012
MW512432_Singapore_2014
KY851404_Philippines_2013
KY851403_Philippines_2013
KY851417_Philippines_2013
KY851431_Philippines_2012
JX891656_Micronesia_2011
JX891657_Micronesia_2011

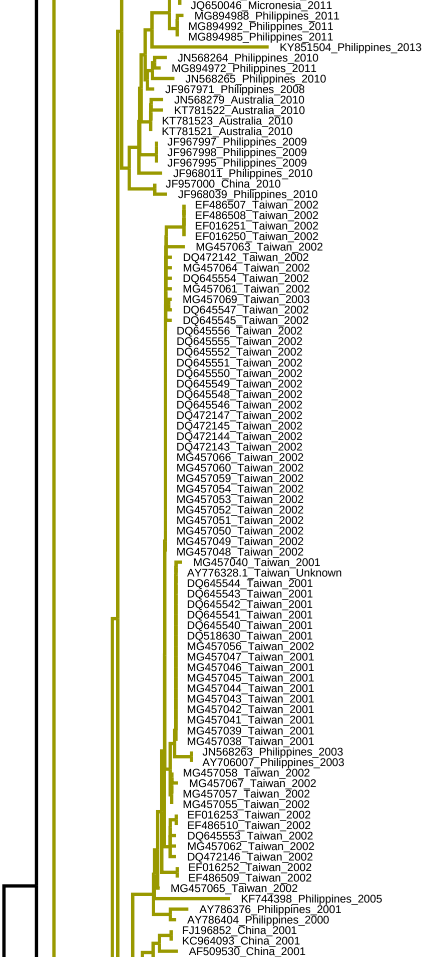
JQ650046_Micronesia_2011
MG894988_Philippines_2011
MG894992_Philippines_2011
MG894985_Philippines_2011
KY851504_Philippines_2013
JN568264_Philippines_2010
MG894972_Philippines_2011
JN568265_Philippines_2010
JF967971_Philippines_2008
JN568279_Australia_2010
KT781522_Australia_2010
KT781523_Australia_2010
KT781521_Australia_2010
JF967997_Philippines_2009
JF967998_Philippines_2009
JF967995_Philippines_2009
JF968011_Philippines_2010
JF957000_China_2010
JF968039_Philippines_2010
EF486507_Taiwan_2002
EF486508_Taiwan_2002
EF016251_Taiwan_2002
EF016250_Taiwan_2002
MG457063_Taiwan_2002
DQ472142_Taiwan_2002
MG457064_Taiwan_2002
DQ645554_Taiwan_2002
MG457061_Taiwan_2002
MG457069_Taiwan_2003
DQ645547_Taiwan_2002
DQ645545_Taiwan_2002
DQ645556_Taiwan_2002
DQ645555_Taiwan_2002
DQ645552_Taiwan_2002
DQ645551_Taiwan_2002
DQ645550_Taiwan_2002
DQ645549_Taiwan_2002
DQ645548_Taiwan_2002
DQ645546_Taiwan_2002
DQ472147_Taiwan_2002
DQ472145_Taiwan_2002
DQ472144_Taiwan_2002
DQ472143_Taiwan_2002
MG457066_Taiwan_2002
MG457060_Taiwan_2002
MG457059_Taiwan_2002
MG457054_Taiwan_2002
MG457053_Taiwan_2002
MG457052_Taiwan_2002
MG457051_Taiwan_2002
MG457050_Taiwan_2002
MG457049_Taiwan_2002
MG457048_Taiwan_2002
MG457040_Taiwan_2001
AY776328.1_Taiwan_Unknown
DQ645544_Taiwan_2001
DQ645543_Taiwan_2001
DQ645542_Taiwan_2001
DQ645541_Taiwan_2001
DQ645540_Taiwan_2001
DQ518630_Taiwan_2001
MG457056_Taiwan_2002
MG457047_Taiwan_2001
MG457046_Taiwan_2001
MG457045_Taiwan_2001
MG457044_Taiwan_2001
MG457043_Taiwan_2001
MG457042_Taiwan_2001
MG457041_Taiwan_2001
MG457039_Taiwan_2001
MG457038_Taiwan_2001
JN568263_Philippines_2003
AY706007_Philippines_2003
MG457058_Taiwan_2002
MG457067_Taiwan_2002
MG457057_Taiwan_2002
MG457055_Taiwan_2002
EF016253_Taiwan_2002
EF486510_Taiwan_2002
DQ645553_Taiwan_2002
MG457062_Taiwan_2002
DQ472146_Taiwan_2002
EF016252_Taiwan_2002
EF486509_Taiwan_2002
MG457065_Taiwan_2002
KF744398_Philippines_2005
AY786376_Philippines_2001
AY786404_Philippines_2000
FJ196852_China_2001
KC964093_China_2001
AF509530_China_2001

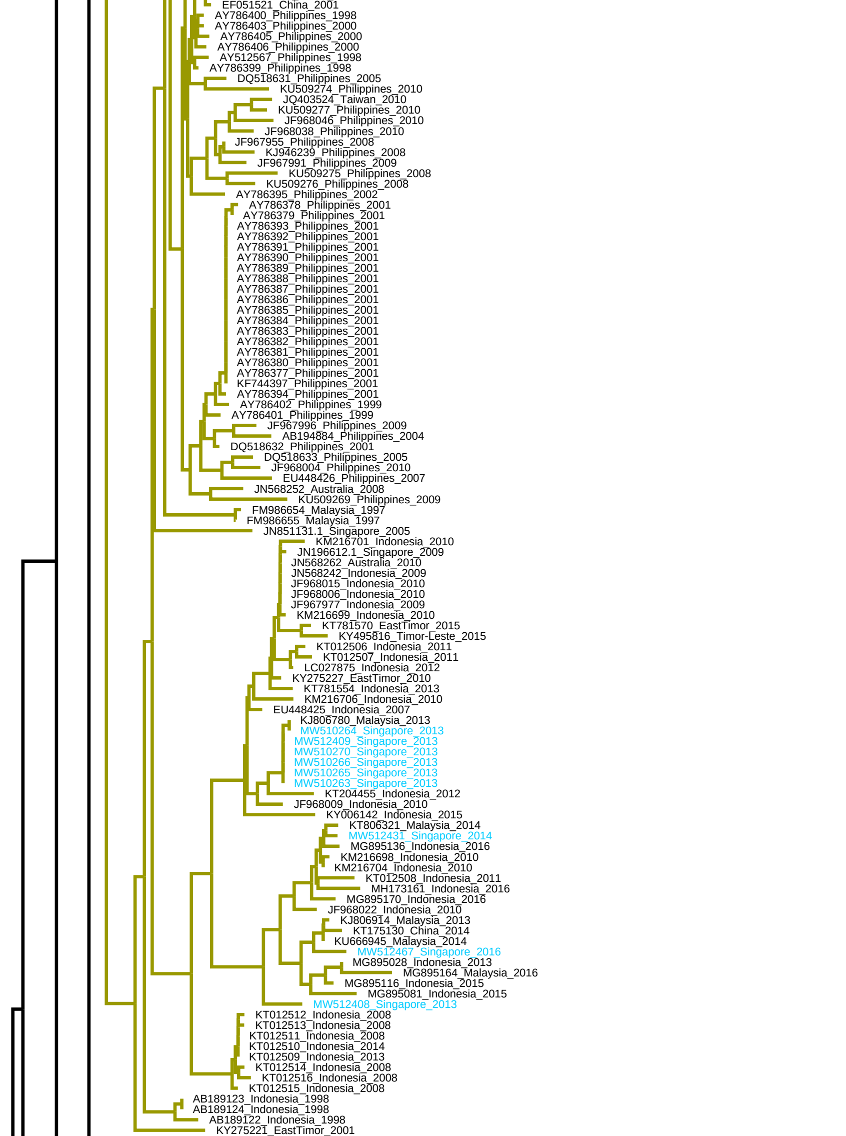
EF051521_China_2001
AY786400_Philippines_1998
AY786403_Philippines_2000
AY786405_Philippines_2000
AY786406_Philippines_2000
AY512567_Philippines_1998
AY786399_Philippines_1998
DQ518631_Philippines_2005
KU509274_Philippines_2010
JQ403524_Taiwan_2010
KU509277_Philippines_2010
JF968046_Philippines_2010
JF968038_Philippines_2010
JF967955_Philippines_2008
KJ946239_Philippines_2008
JF967991_Philippines_2009
KU509275_Philippines_2008
KU509276_Philippines_2008
AY786395_Philippines_2002
AY786378_Philippines_2001
AY786379_Philippines_2001
AY786393_Philippines_2001
AY786392_Philippines_2001
AY786391_Philippines_2001
AY786390_Philippines_2001
AY786389_Philippines_2001
AY786388_Philippines_2001
AY786387_Philippines_2001
AY786386_Philippines_2001
AY786385_Philippines_2001
AY786384_Philippines_2001
AY786383_Philippines_2001
AY786382_Philippines_2001
AY786381_Philippines_2001
AY786380_Philippines_2001
AY786377_Philippines_2001
KF744397_Philippines_2001
AY786394_Philippines_2001
AY786402_Philippines_1999
AY786401_Philippines_1999
JF967996_Philippines_2009
AB194884_Philippines_2004
DQ518632_Philippines_2001
DQ518633_Philippines_2005
JF968004_Philippines_2010
EU448426_Philippines_2007
JN568252_Australia_2008
KU509269_Philippines_2009
FM986654_Malaysia_1997
FM986655_Malaysia_1997
JN851131.1_Singapore_2005
KM216701_Indonesia_2010
JN196612.1_Singapore_2009
JN568262_Australia_2010
JN568242_Indonesia_2009
JF968015_Indonesia_2010
JF968006_Indonesia_2010
JF967977_Indonesia_2009
KM216699_Indonesia_2010
KT781570_EastTimor_2015
KY495816_Timor-Leste_2015
KT012506_Indonesia_2011
KT012507_Indonesia_2011
LC027875_Indonesia_2012
KY275227_EastTimor_2010
KT781554_Indonesia_2013
KM216706_Indonesia_2010
EU448425_Indonesia_2007
KJ806780_Malaysia_2013
MW510264_Singapore_2013
MW512409_Singapore_2013
MW510270_Singapore_2013
MW510266_Singapore_2013
MW510265_Singapore_2013
MW510263_Singapore_2013
KT204455_Indonesia_2012
JF968009_Indonesia_2010
KY006142_Indonesia_2015
KT806321_Malaysia_2014
MW512431_Singapore_2014
MG895136_Indonesia_2016
KM216698_Indonesia_2010
KM216704_Indonesia_2010
KT012508_Indonesia_2011
MH173161_Indonesia_2016
MG895170_Indonesia_2016
JF968022_Indonesia_2010
KJ806914_Malaysia_2013
KT175130_China_2014
KU666945_Malaysia_2014
MW512467_Singapore_2016
MG895028_Indonesia_2013
MG895164_Malaysia_2016
MG895116_Indonesia_2015
MG895081_Indonesia_2015
MW512408_Singapore_2013
KT012512_Indonesia_2008
KT012513_Indonesia_2008
KT012511_Indonesia_2008
KT012510_Indonesia_2014
KT012509_Indonesia_2013
KT012514_Indonesia_2008
KT012516_Indonesia_2008
KT012515_Indonesia_2008
AB189123_Indonesia_1998
AB189124_Indonesia_1998
AB189122_Indonesia_1998
KY275221_EastTimor_2001

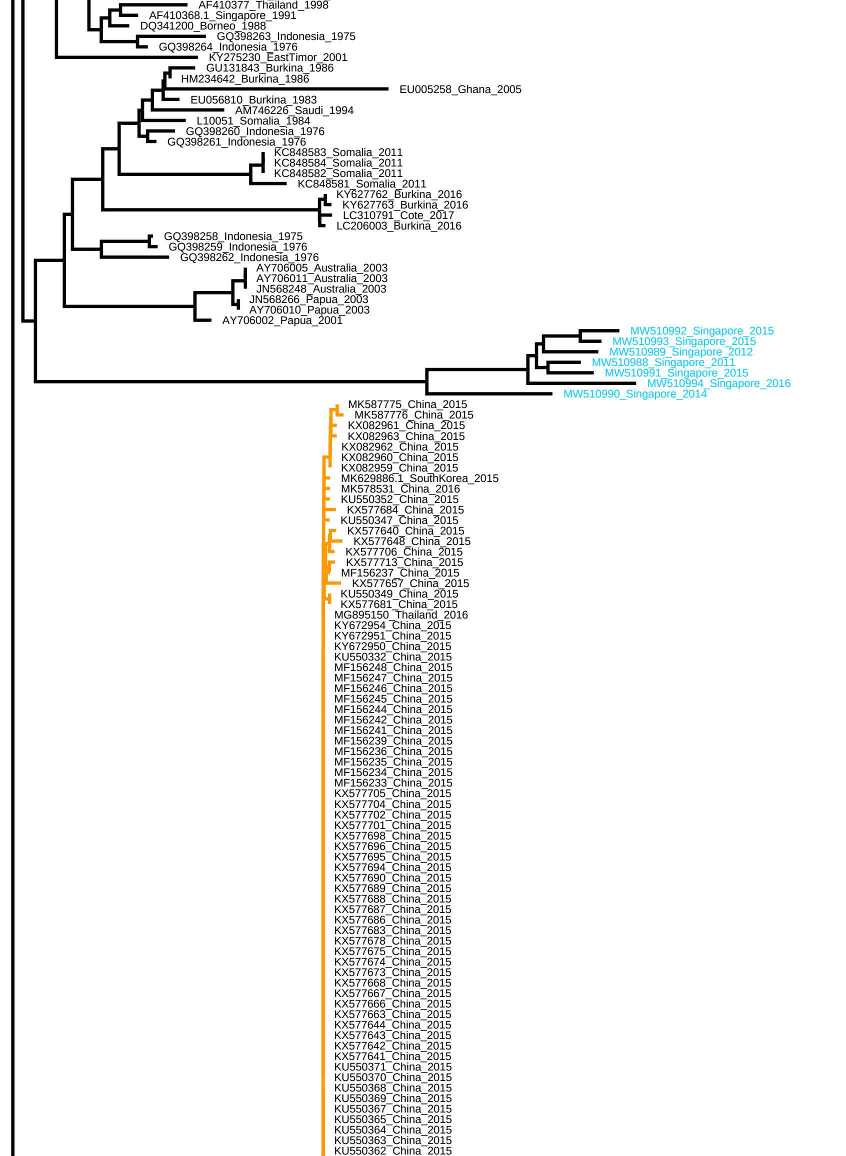
AF410377_Thailand_1998
AF410368.1_Singapore_1991
DQ341200_Borneo_1988
GQ398263_Indonesia_1975
GQ398264_Indonesia_1976
KY275230_EastTimor_2001
GU131843_Burkina_1986
HM234642_Burkina_1986
EU005258_Ghana_2005
EU056810_Burkina_1983
AM746226_Saudi_1994
L10051_Somalia_1984
GQ398260_Indonesia_1976
GQ398261_Indonesia_1976
KC848583_Somalia_2011
KC848584_Somalia_2011
KC848582_Somalia_2011
KC848581_Somalia_2011
KY627762_Burkina_2016
KY627763_Burkina_2016
LC310791_Cote_2017
LC206003_Burkina_2016
GQ398258_Indonesia_1975
GQ398259_Indonesia_1976
GQ398262_Indonesia_1976
AY706005_Australia_2003
AY706011_Australia_2003
JN568248_Australia_2003
JN568266_Papua_2003
AY706010_Papua_2003
AY706002_Papua_2001
MW510992_Singapore_2015
MW510993_Singapore_2015
MW510989_Singapore_2012
MW510988_Singapore_2011
MW510991_Singapore_2015
MW510994_Singapore_2016
MW510990_Singapore_2014
MK587775_China_2015
MK587776_China_2015
KX082961_China_2015
KX082963_China_2015
KX082962_China_2015
KX082960_China_2015
KX082959_China_2015
MK629886.1_SouthKorea_2015
MK578531_China_2016
KU550352_China_2015
KX577684_China_2015
KU550347_China_2015
KX577640_China_2015
KX577648_China_2015
KX577706_China_2015
KX577713_China_2015
MF156237_China_2015
KX577657_China_2015
KU550349_China_2015
KX577681_China_2015
MG895150_Thailand_2016
KY672954_China_2015
KY672951_China_2015
KY672950_China_2015
KU550332_China_2015
MF156248_China_2015
MF156247_China_2015
MF156246_China_2015
MF156245_China_2015
MF156244_China_2015
MF156242_China_2015
MF156241_China_2015
MF156239_China_2015
MF156236_China_2015
MF156235_China_2015
MF156234_China_2015
MF156233_China_2015
KX577705_China_2015
KX577704_China_2015
KX577702_China_2015
KX577701_China_2015
KX577698_China_2015
KX577696_China_2015
KX577695_China_2015
KX577694_China_2015
KX577690_China_2015
KX577689_China_2015
KX577688_China_2015
KX577687_China_2015
KX577686_China_2015
KX577683_China_2015
KX577678_China_2015
KX577675_China_2015
KX577674_China_2015
KX577673_China_2015
KX577668_China_2015
KX577667_China_2015
KX577666_China_2015
KX577663_China_2015
KX577644_China_2015
KX577643_China_2015
KX577642_China_2015
KX577641_China_2015
KU550371_China_2015
KU550370_China_2015
KU550368_China_2015
KU550369_China_2015
KU550367_China_2015
KU550365_China_2015
KU550364_China_2015
KU550363_China_2015
KU550362_China_2015

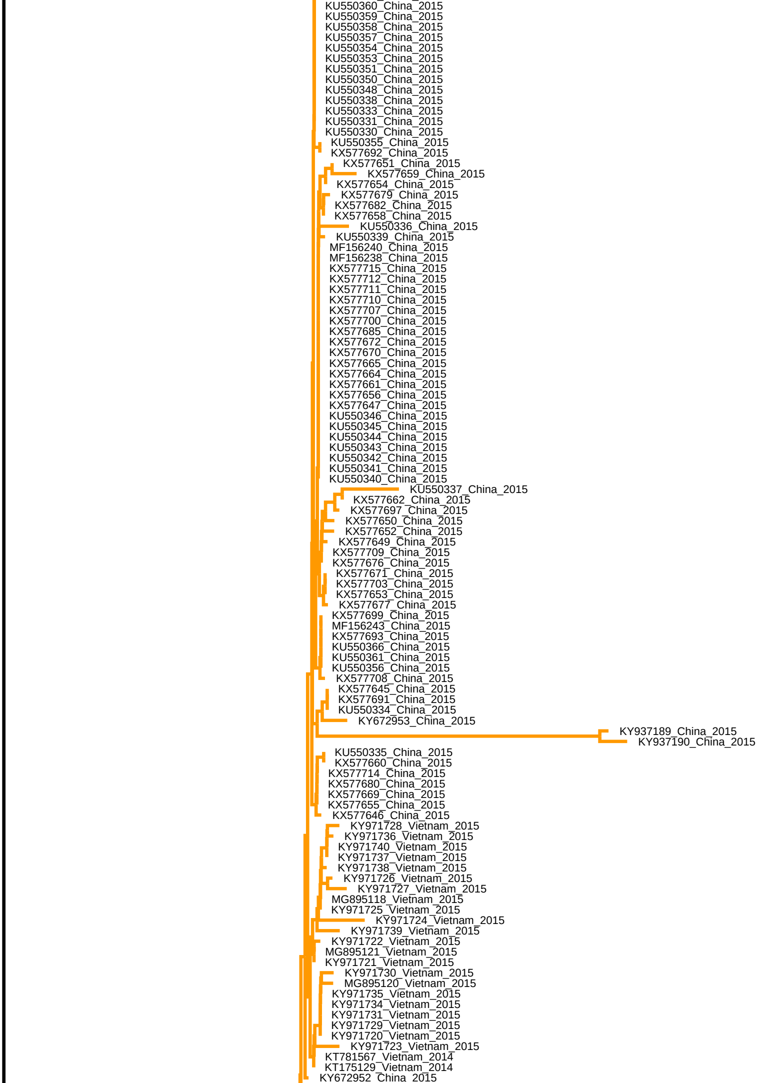
KU550360_China_2015
KU550359_China_2015
KU550358_China_2015
KU550357_China_2015
KU550354_China_2015
KU550353_China_2015
KU550351_China_2015
KU550350_China_2015
KU550348_China_2015
KU550338_China_2015
KU550333_China_2015
KU550331_China_2015
KU550330_China_2015
KU550355_China_2015
KX577692_China_2015
KX577651_China_2015
KX577659_China_2015
KX577654_China_2015
KX577679_China_2015
KX577682_China_2015
KX577658_China_2015
KU550336_China_2015
KU550339_China_2015
MF156240_China_2015
MF156238_China_2015
KX577715_China_2015
KX577712_China_2015
KX577711_China_2015
KX577710_China_2015
KX577707_China_2015
KX577700_China_2015
KX577685_China_2015
KX577672_China_2015
KX577670_China_2015
KX577665_China_2015
KX577664_China_2015
KX577661_China_2015
KX577656_China_2015
KX577647_China_2015
KU550346_China_2015
KU550345_China_2015
KU550344_China_2015
KU550343_China_2015
KU550342_China_2015
KU550341_China_2015
KU550340_China_2015
KU550337_China_2015
KX577662_China_2015
KX577697_China_2015
KX577650_China_2015
KX577652_China_2015
KX577649_China_2015
KX577709_China_2015
KX577676_China_2015
KX577671_China_2015
KX577703_China_2015
KX577653_China_2015
KX577677_China_2015
KX577699_China_2015
MF156243_China_2015
KX577693_China_2015
KU550366_China_2015
KU550361_China_2015
KU550356_China_2015
KX577708_China_2015
KX577645_China_2015
KX577691_China_2015
KU550334_China_2015
KY672953_China_2015
KY937189_China_2015
KY937190_China_2015
KU550335_China_2015
KX577660_China_2015
KX577714_China_2015
KX577680_China_2015
KX577669_China_2015
KX577655_China_2015
KX577646_China_2015
KY971728_Vietnam_2015
KY971736_Vietnam_2015
KY971740_Vietnam_2015
KY971737_Vietnam_2015
KY971738_Vietnam_2015
KY971726_Vietnam_2015
KY971727_Vietnam_2015
MG895118_Vietnam_2015
KY971725_Vietnam_2015
KY971724_Vietnam_2015
KY971739_Vietnam_2015
KY971722_Vietnam_2015
MG895121_Vietnam_2015
KY971721_Vietnam_2015
KY971730_Vietnam_2015
MG895120_Vietnam_2015
KY971735_Vietnam_2015
KY971734_Vietnam_2015
KY971731_Vietnam_2015
KY971729_Vietnam_2015
KY971720_Vietnam_2015
KY971723_Vietnam_2015
KT781567_Vietnam_2014
KT175129_Vietnam_2014
KY672952_China_2015

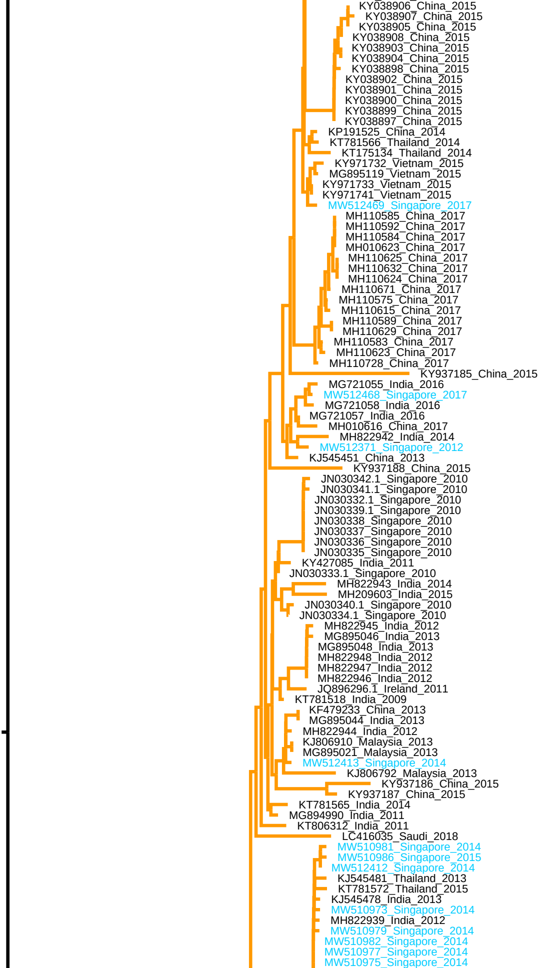

KY038906_China_2015
KY038907_China_2015
KY038905_China_2015
KY038908_China_2015
KY038903_China_2015
KY038904_China_2015
KY038898_China_2015
KY038902_China_2015
KY038901_China_2015
KY038900_China_2015
KY038899_China_2015
KY038897_China_2015
KP191525_China_2014
KT781566_Thailand_2014
KT175134_Thailand_2014
KY971732_Vietnam_2015
MG895119_Vietnam_2015
KY971733_Vietnam_2015
KY971741_Vietnam_2015
MW512469_Singapore_2017
MH110585_China_2017
MH110592_China_2017
MH110584_China_2017
MH010623_China_2017
MH110625_China_2017
MH110632_China_2017
MH110624_China_2017
MH110671_China_2017
MH110575_China_2017
MH110615_China_2017
MH110589_China_2017
MH110629_China_2017
MH110583_China_2017
MH110623_China_2017
MH110728_China_2017
KY937185_China_2015
MG721055_India_2016
MW512468_Singapore_2017
MG721058_India_2016
MG721057_India_2016
MH010616_China_2017
MH822942_India_2014
MW512371_Singapore_2012
KJ545451_China_2013
KY937188_China_2015
JN030342.1_Singapore_2010
JN030341.1_Singapore_2010
JN030332.1_Singapore_2010
JN030339.1_Singapore_2010
JN030338_Singapore_2010
JN030337_Singapore_2010
JN030336_Singapore_2010
JN030335_Singapore_2010
KY427085_India_2011
JN030333.1_Singapore_2010
MH822943_India_2014
MH209603_India_2015
JN030340.1_Singapore_2010
JN030334.1_Singapore_2010
MH822945_India_2012
MG895046_India_2013
MG895048_India_2013
MH822948_India_2012
MH822947_India_2012
MH822946_India_2012
JQ896296.1_Ireland_2011
KT781518_India_2009
KF479233_China_2013
MG895044_India_2013
MH822944_India_2012
KJ806910_Malaysia_2013
MG895021_Malaysia_2013
MW512413_Singapore_2014
KJ806792_Malaysia_2013
KY937186_China_2015
KY937187_China_2015
KT781565_India_2014
MG894990_India_2011
KT806312_India_2011
LC416035_Saudi_2018
MW510981_Singapore_2014
MW510986_Singapore_2015
MW512412_Singapore_2014
KJ545481_Thailand_2013
KT781572_Thailand_2015
KJ545478_India_2013
MW510973_Singapore_2014
MH822939_India_2012
MW510979_Singapore_2014
MW510982_Singapore_2014
MW510977_Singapore_2014
MW510975_Singapore_2014

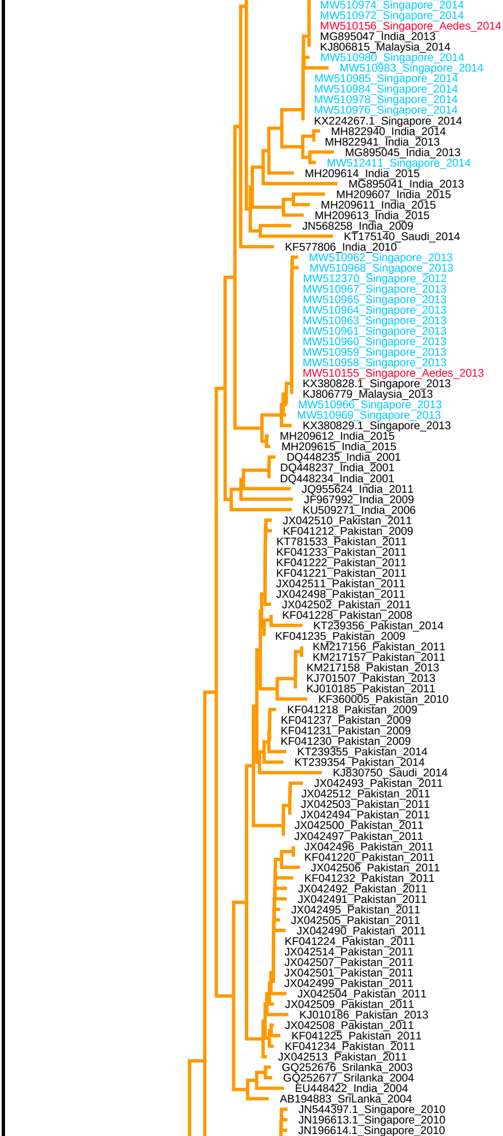
MW510974_Singapore_2014
MW510972_Singapore_2014
MW510156_Singapore_Aedes_2014
MG895047_India_2013
KJ806815_Malaysia_2014
MW510980_Singapore_2014
MW510983_Singapore_2014
MW510985_Singapore_2014
MW510984_Singapore_2014
MW510978_Singapore_2014
MW510976_Singapore_2014
KX224267.1_Singapore_2014
MH822940_India_2014
MH822941_India_2013
MG895045_India_2013
MW512411_Singapore_2014
MH209614_India_2015
MG895041_India_2013
MH209607_India_2015
MH209611_India_2015
MH209613_India_2015
JN568258_India_2009
KT175140_Saudi_2014
KF577806_India_2010
MW510962_Singapore_2013
MW510968_Singapore_2013
MW512370_Singapore_2012
MW510967_Singapore_2013
MW510965_Singapore_2013
MW510964_Singapore_2013
MW510963_Singapore_2013
MW510961_Singapore_2013
MW510960_Singapore_2013
MW510959_Singapore_2013
MW510958_Singapore_2013
MW510155_Singapore_Aedes_2013
KX380828.1_Singapore_2013
KJ806779_Malaysia_2013
MW510966_Singapore_2013
MW510969_Singapore_2013
KX380829.1_Singapore_2013
MH209612_India_2015
MH209615_India_2015
DQ448235_India_2001
DQ448237_India_2001
DQ448234_India_2001
JQ955624_India_2011
JF967992_India_2009
KU509271_India_2006
JX042510_Pakistan_2011
KF041212_Pakistan_2009
KT781533_Pakistan_2011
KF041233_Pakistan_2011
KF041222_Pakistan_2011
KF041221_Pakistan_2011
JX042511_Pakistan_2011
JX042498_Pakistan_2011
JX042502_Pakistan_2011
KF041228_Pakistan_2008
KT239356_Pakistan_2014
KF041235_Pakistan_2009
KM217156_Pakistan_2011
KM217157_Pakistan_2011
KM217158_Pakistan_2013
KJ701507_Pakistan_2013
KJ010185_Pakistan_2011
KF360005_Pakistan_2010
KF041218_Pakistan_2009
KF041237_Pakistan_2009
KF041231_Pakistan_2009
KF041230_Pakistan_2009
KT239355_Pakistan_2014
KT239354_Pakistan_2014
KJ830750_Saudi_2014
JX042493_Pakistan_2011
JX042512_Pakistan_2011
JX042503_Pakistan_2011
JX042494_Pakistan_2011
JX042500_Pakistan_2011
JX042497_Pakistan_2011
JX042496_Pakistan_2011
KF041220_Pakistan_2011
JX042506_Pakistan_2011
KF041232_Pakistan_2011
JX042492_Pakistan_2011
JX042491_Pakistan_2011
JX042495_Pakistan_2011
JX042505_Pakistan_2011
JX042490_Pakistan_2011
KF041224_Pakistan_2011
JX042514_Pakistan_2011
JX042507_Pakistan_2011
JX042501_Pakistan_2011
JX042499_Pakistan_2011
JX042504_Pakistan_2011
JX042509_Pakistan_2011
KJ010186_Pakistan_2013
JX042508_Pakistan_2011
KF041225_Pakistan_2011
KF041234_Pakistan_2011
JX042513_Pakistan_2011
GQ252676_Srilanka_2003
GQ252677_Srilanka_2004
EU448422_India_2004
AB194883_SriLanka_2004
JN544397.1_Singapore_2010
JN196613.1_Singapore_2010
JN196614.1_Singapore_2010

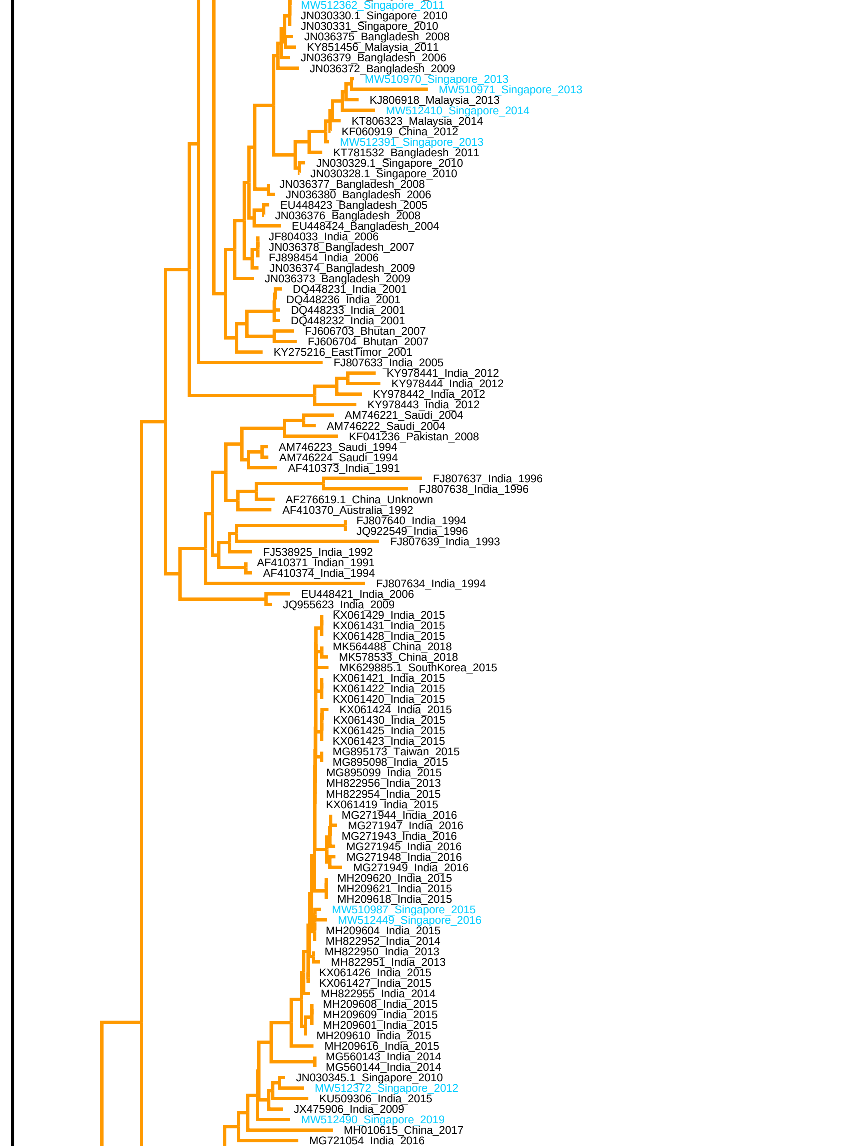

MW512362_Singapore_2011
JN030330.1_Singapore_2010
JN030331_Singapore_2010
JN036375_Bangladesh_2008
KY851456_Malaysia_2011
JN036379_Bangladesh_2006
JN036372_Bangladesh_2009
MW510970_Singapore_2013
MW510971_Singapore_2013
KJ806918_Malaysia_2013
MW512410_Singapore_2014
KT806323_Malaysia_2014
KF060919_China_2012
MW512391_Singapore_2013
KT781532_Bangladesh_2011
JN030329.1_Singapore_2010
JN030328.1_Singapore_2010
JN036377_Bangladesh_2008
JN036380_Bangladesh_2006
EU448423_Bangladesh_2005
JN036376_Bangladesh_2008
EU448424_Bangladesh_2004
JF804033_India_2006
JN036378_Bangladesh_2007
FJ898454_India_2006
JN036374_Bangladesh_2009
JN036373_Bangladesh_2009
DQ448231_India_2001
DQ448236_India_2001
DQ448233_India_2001
DQ448232_India_2001
FJ606703_Bhutan_2007
FJ606704_Bhutan_2007
KY275216_EastTimor_2001
FJ807633_India_2005
KY978441_India_2012
KY978444_India_2012
KY978442_India_2012
KY978443_India_2012
AM746221_Saudi_2004
AM746222_Saudi_2004
KF041236_Pakistan_2008
AM746223_Saudi_1994
AM746224_Saudi_1994
AF410373_India_1991
FJ807637_India_1996
FJ807638_India_1996
AF276619.1_China_Unknown
AF410370_Australia_1992
FJ807640_India_1994
JQ922549_India_1996
FJ807639_India_1993
FJ538925_India_1992
AF410371_Indian_1991
AF410374_India_1994
FJ807634_India_1994
EU448421_India_2006
JQ955623_India_2009
KX061429_India_2015
KX061431_India_2015
KX061428_India_2015
MK564488_China_2018
MK578533_China_2018
MK629885.1_SouthKorea_2015
KX061421_India_2015
KX061422_India_2015
KX061420_India_2015
KX061424_India_2015
KX061430_India_2015
KX061425_India_2015
KX061423_India_2015
MG895173_Taiwan_2015
MG895098_India_2015
MG895099_India_2015
MH822956_India_2013
MH822954_India_2015
KX061419_India_2015
MG271944_India_2016
MG271947_India_2016
MG271943_India_2016
MG271945_India_2016
MG271948_India_2016
MG271949_India_2016
MH209620_India_2015
MH209621_India_2015
MH209618_India_2015
MW510987_Singapore_2015
MW512449_Singapore_2016
MH209604_India_2015
MH822952_India_2014
MH822950_India_2013
MH822951_India_2013
KX061426_India_2015
KX061427_India_2015
MH822955_India_2014
MH209608_India_2015
MH209609_India_2015
MH209601_India_2015
MH209610_India_2015
MH209616_India_2015
MG560143_India_2014
MG560144_India_2014
JN030345.1_Singapore_2010
MW512372_Singapore_2012
KU509306_India_2015
JX475906_India_2009
MW512490_Singapore_2019
MH010615_China_2017
MG721054_India_2016

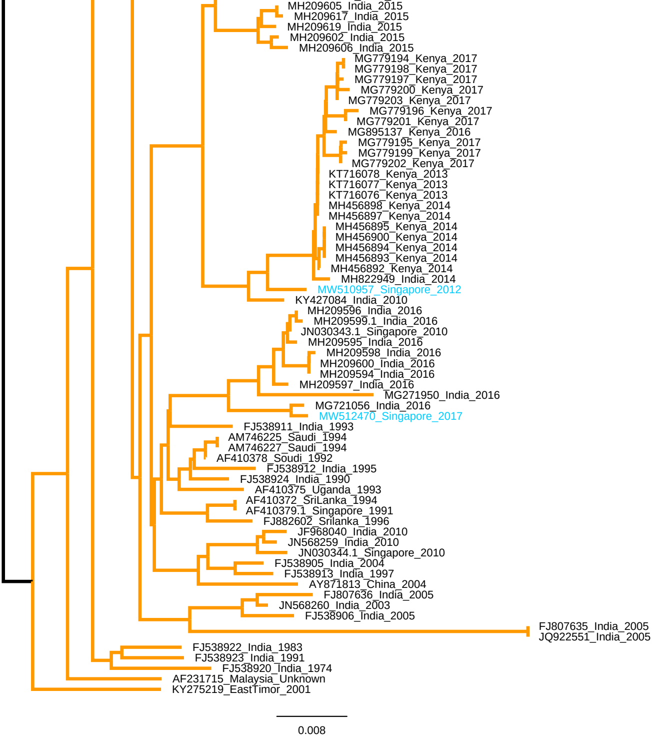
MH209605_India_2015
MH209617_India_2015
MH209619_India_2015
MH209602_India_2015
MH209606_India_2015
MG779194_Kenya_2017
MG779198_Kenya_2017
MG779197_Kenya_2017
MG779200_Kenya_2017
MG779203_Kenya_2017
MG779196_Kenya_2017
MG779201_Kenya_2017
MG895137_Kenya_2016
MG779195_Kenya_2017
MG779199_Kenya_2017
MG779202_Kenya_2017
KT716078_Kenya_2013
KT716077_Kenya_2013
KT716076_Kenya_2013
MH456898_Kenya_2014
MH456897_Kenya_2014
MH456895_Kenya_2014
MH456900_Kenya_2014
MH456894_Kenya_2014
MH456893_Kenya_2014
MH456892_Kenya_2014
MH822949_India_2014
MW510957_Singapore_2012
KY427084_India_2010
MH209596_India_2016
MH209599.1_India_2016
JN030343.1_Singapore_2010
MH209595_India_2016
MH209598_India_2016
MH209600_India_2016
MH209594_India_2016
MH209597_India_2016
MG271950_India_2016
MG721056_India_2016
MW512470_Singapore_2017
FJ538911_India_1993
AM746225_Saudi_1994
AM746227_Saudi_1994
AF410378_Soudi_1992
FJ538912_India_1995
FJ538924_India_1990
AF410375_Uganda_1993
AF410372_SriLanka_1994
AF410379.1_Singapore_1991
FJ882602_Srilanka_1996
JF968040_India_2010
JN568259_India_2010
JN030344.1_Singapore_2010
FJ538905_India_2004
FJ538913_India_1997
AY871813_China_2004
FJ807636_India_2005
JN568260_India_2003
FJ538906_India_2005
FJ807635_India_2005
JQ922551_India_2005
FJ538922_India_1983
FJ538923_India_1991
FJ538920_India_1974
AF231715_Malaysia_Unknown
KY275219_EastTimor_2001
0.008
